# What Causes the Onset of Psychosis in Individuals at Clinical High Risk? A Meta-analysis of Risk and Protective Factors

**DOI:** 10.1093/schbul/sbz039

**Published:** 2019-06-20

**Authors:** Dominic Oliver, Thomas J Reilly, Ottone Baccaredda Boy, Natalia Petros, Cathy Davies, Stefan Borgwardt, Philip McGuire, Paolo Fusar-Poli

**Affiliations:** 1 Early Psychosis: Interventions and Clinical detection (EPIC) Lab, Department of Psychosis Studies, Institute of Psychiatry, Psychology and Neuroscience, King’s College London, London, UK; 2 Department of Psychosis Studies, Institute of Psychiatry, Psychology and Neuroscience, King’s College London, London, UK; 3 Department of Psychiatry, University of Basel, Basel, Switzerland; 4 OASIS Service, South London and the Maudsley NHS National Health Service Foundation Trust, London, UK; 5 Department of Brain and Behavioural Sciences, University of Pavia, Pavia, Italy; 6 National Institute of Health Research, Mental Health, Translational Research Collaboration, Early Psychosis Workstream, UK

**Keywords:** psychosis, clinical high risk, risk, symptoms, functioning, schizophrenia

## Abstract

Twenty percent of individuals at clinical high risk for psychosis (CHR-P) develop the disorder within 2 years. Extensive research has explored the factors that differentiate those who develop psychosis and those who do not, but the results are conflicting.

The current systematic review and meta-analysis comprehensively addresses the consistency and magnitude of evidence for non-purely genetic risk and protective factors associated with the risk of developing psychosis in CHR-P individuals. Random effects meta-analyses, standardized mean difference (SMD) and odds ratio (OR) were used, in combination with an established stratification of evidence that assesses the association of each factor and the onset of psychotic disorders (from class I, convincing evidence to class IV weak evidence), while controlling for several types of biases.

A total of 128 original controlled studies relating to 26 factors were retrieved. No factors showed class I-convincing evidence. Two further factors were associated with class II-highly suggestive evidence: attenuated positive psychotic symptoms (SMD = 0.348, 95% CI: 0.280, 0.415) and global functioning (SMD = −0.291, 95% CI: −0.370, −0.211). There was class III-suggestive evidence for negative psychotic symptoms (SMD = 0.393, 95% CI: 0.317, 0.469). There was either class IV-weak or no evidence for all other factors.

Our findings suggest that despite the large number of putative risk factors investigated in the literature, only attenuated positive psychotic symptoms, global functioning, and negative psychotic symptoms show suggestive evidence or greater for association with transition to psychosis. The current findings may inform the refinement of clinical prediction models and precision medicine in this field.

## Introduction

The introduction of the first Clinical High Risk for Psychosis (CHR-P^1^) service, the PACE clinic,^2^ has stimulated extensive research into psychosis prevention, to the point that the CHR-P construct has become a key component of clinical services for early intervention^3^ (eg NICE [National Institute for Health and Care Excellence] guidelines^4^; NHS England Access, and Waiting Time standard^5^). Simultaneously, some challenges have emerged, such as the need to refine the prediction of outcomes.^6^ A key limitation is that the level of risk observed in CHR-P individuals is mostly accounted for by their sampling.^7^ For example, when CHR-P criteria are applied to the general population, the level of risk of individuals meeting them is very low.^8–10^ An additional problem is that there is poor knowledge about factors that modulate the level of risk in these individuals, because their identification and outcomes are entirely predicated on the basis of symptoms. However, symptoms represent an epiphenomenon of an underlying etiopathology. In fact, the overarching model underlying the development of psychosis involves the culmination of genetic and environmental factors that can increase (risk factors) or decrease (protective factors) the likelihood of developing psychosis, as well as the interaction between them.^11,12^ It is therefore essential to better understand the role of specific risk and protective factors in this area. Accordingly, we have recently published an umbrella review (a review of reviews) to quantitatively synthesize the existing literature on risk/protective factors for psychosis in the general population.^12^ In a companion meta-analysis we confirmed that CHR-P individuals accumulate several environmental risk factors for psychosis, like childhood trauma, adverse life events and affective dysfunction, compared to controls, whereas the role of genetic and epigenetic risk factors in this group awaits clarification.^13^ The effect of different risk/protective factors on the risk of developing and later transition to psychosis within individuals who have met CHR-P criteria has yet to be clarified at a meta-analytical level.

Despite much research into risk/protective factors potentially associated with transition to psychosis in CHR-P samples, the studies are often small, underpowered or inconsistent in their results. Meta-analytical methods can address these issues. Reviewing risk/protective factors for the development of psychosis within CHR-P samples is relevant two-fold. First, although we know that 20% of CHR-P individuals transition to psychosis within 2 years,^14^ we are currently unable to predict who will transition and who will not. Greater understanding of the specific risk/protective factors that modify risk of transition at the individual subject level will allow for improved prognostication. Second, some factors may be potentially modifiable, therefore allowing for novel, individualized therapeutic strategies; improving primary indicated prevention of psychosis.

To the best of our knowledge, this study is the first meta-analysis to quantitatively synthesize the evidence for risk/protective factors for developing psychosis in CHR-P individuals. The primary aim was to systematically review the evidence for risk/protective factors within the CHR-P population and to provide a meta-analytical summary of their magnitude, direction of effect and consistency, controlling for several biases (eg small study effect and excess significance bias). The latter point will be achieved by complementing the standard pairwise meta-analysis with the use of validated criteria that have been developed for umbrella reviews^12,15–17^ to stratify the evidence of association between risk/protective factors and outcomes.

## Methods

### Search Strategies

The Preferred Reporting Items for Systematic Reviews and Meta-analyses (PRISMA)^18^ and Meta-analysis of Observational Studies in Epidemiology (MOOSE) guidelines^19^ were adhered to throughout to achieve high quality of reporting (supplementary tables 1 and 2). Details of the protocol for systematic review were registered on PROSPERO (CRD42017077470).

A two-step systematic search of the literature was performed by two independent researchers (T.R. and O.B.B.) to identify relevant studies investigating the effect of risk and protective factors for transition to psychosis in CHR-P individuals.

The Ovid database by Wolters Kluwer (including MEDLINE, EMBASE, and PsycINFO) was searched. Full search strategy including keywords can be seen in supplementary methods 1. The search was extended from inception until 13th May 2018.

### Inclusion Criteria

Articles meeting the inclusion criteria for the current systematic review and meta-analysis were the following: (a) original articles, written in English (b) cohort studies examining the association between risk/protective factors and psychotic disorders in the CHR-P population (c) included CHR-P individuals defined by standard psychometric instruments: Comprehensive Assessment of At Risk Mental States (CAARMS)^20^; Brief Psychiatric Rating Scale (BPRS)^21^; Structured Interview for Psychosis-risk Syndromes (SIPS)^22^; Basel Screening Instrument for Psychosis (BSIP)^23^ (d) reported transitions to a psychotic disorder as a key outcome measure, defined according to standard international *Diagnostic and Statistical Manual of Mental Disorders* (*DSM*) or *International Statistical Classification of Diseases and Related Health Problems* (*ICD*) criteria—any version—(e) reported follow-up of at least 1 year, based on meta-analytical evidence suggesting that shorter follow-up times may be associated with infrequent events^24^ (transitions to psychosis) resulting in underpowered studies.

### Exclusion Criteria

In line with our previous work^12,13^ we excluded biomarkers, purely genetic factors, and cognitive factors, because these would require a different and specific meta-analytical approach. Furthermore, despite advances in genetic understanding in this field (eg polygenic risk scores), our understanding is still relatively limited, whereas the role of biomarkers^25^ and cognition^26^ has already been meta-analyzed by our group. As such, we excluded: (a) conference abstracts, reviews, case-reports, cross-sectional studies, and case–control studies, (c) purely genetic factors, (d) biomarkers or cognitive factors, (d) studies using CHR-P definitions other than those listed earlier.

### Data Extraction

Data extraction was done independently by two investigators (T.R. and O.B.B.). Any discrepancies were resolved in consensus meetings with another author (D.O.) under the supervision of a senior researcher (P.F.P.). Data selection and extraction were based on a systematic approach that is further detailed in supplementary methods 2. For continuous factors we also considered the mean baseline value in the transition and mean baseline value in the non-transition group. The factors were grouped in the following domains that had no influence on the statistical analyses, in line with previous studies in this area: sociodemographic/parental factors, later factors, antecedents, and symptom scores/clinical factors.^12,27,28^ Details of risk of bias assessment can be found in supplementary methods 3.

### Statistical Analysis

#### Standard Pairwise Meta-analysis

The meta-analytical effect-size measure was odds ratio (OR) for dichotomous factors and standardized mean difference (SMD) for continuous factors. An OR greater than 1 or an SMD greater than 0 indicated that the factor was associated with an increased likelihood of psychotic disorders. OR lower than 1 or SMD lower than 0 indicated that the factor was associated with a reduced likelihood of psychotic disorders, ie it was protective.

The meta-analysis investigated each specific risk/protective factor without providing pooled estimates (within-subgroup summary effects) as they were felt to be clinically uninterpretable. In the case of studies reporting different definitions of the same outcome measure (eg reporting both CAARMS and BPRS for symptom scores), a mean effect size and an estimate of the variance based on the calculated weight of the included definitions was computed.

Random-effects models were used to control for heterogeneity.

#### Hierarchical Classification of the Evidence

In line with previous studies using umbrella review criteria for classifying the evidence of association between risk/protective factors and health disorders,^12,15–17^ analyses included the following: (a) an Egger test to assess small‐study effects that lead to potential reporting or publication bias^29^, and (b) a test of excess significance bias.^30^ The test of excess significance bias consisted of a binomial test to compare the observed vs the expected number of studies yielding statistically significant results. This expected number was calculated as the sum of the statistical power of the studies. Small‐study effects and excess significance bias were claimed at one‐sided *P* values <0.05, as in previous studies.^15^

The levels of evidence of the associations between putative risk/protective factors and transition to psychotic disorder were then classified according to the guidelines for umbrella reviews^31^: convincing (class I) when number of cases >1000, *P* < 10^−6^, *I*^2^ < 50%, 95% prediction interval excluding the null, no small‐study effects, and no excess significance bias; highly suggestive (class II) when number of cases >1000, *P* < 10^−6^, largest study with a statistically significant effect, and class I criteria not met; suggestive (class III) when number of cases > 1000, *P* < 10^−3^, and class I–II criteria not met; weak (class IV) when *P* < 0.05 and class I–III criteria not met; non‐significant when *P* > 0.05.

Finally, a sensitivity analysis was conducted for the factors classified as class I–III by using only prospective studies. Prospective studies allow one to address the temporality of the association, thus dealing with the problem of reverse causation.^16,17^

Analyses were carried out using Comprehensive Meta Analysis, version 3, and Stata 14.

## Results

### Database

**Fig. 1.**
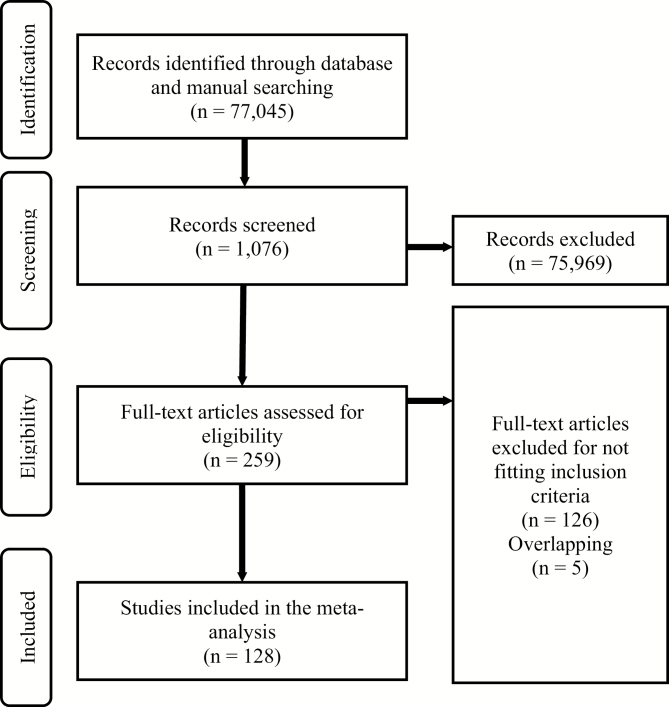
Preferred Reporting Items for Systematic Reviews and Meta-analyses (PRISMA) flowchart outlining study selection process.

Overall, 77 045 records were searched, 259 were screened and 128 were eligible (see figure 1). The eligible articles were published between 1998 (shortly after the first CHR-P service was established) and 13th May 2018.

Overall, the 128 eligible studies comprising 17 967 patients reported on 26 putative risk/protective factors of transition to psychotic disorders for CHR-P individuals (supplementary table 3). These 26 putative risk/protective factors were separated for descriptive purposes into four categories: sociodemographic/parental factors, later factors, antecedents and symptom scores/clinical factors.

The number of cases was greater than 1000 for five factors (19.2%). Of the 26 analyzed factors, 11 had significant associations with psychosis (34.6%), with eight (30.8%) reaching *P* < 0.001 (tables 1–4). Nine factors (34.6%) presented a large heterogeneity (*I*^2^ > 50%). In addition, the evidence for small-study effects was noted for 10 of the 23 factors (43.5%) with enough studies for this to be conducted.

**Table 1.** Level of Evidence for the Association of Sociodemographic/Parental Factors and Psychotic Disorders

Risk Factor	*K*	Random Effects Measures, ES (95% CI)	*N*	*P* Random Effects	*I* ^2^ (*P*)	PI (95% CI)	LS	SSE/ESB	eOR	CE
*Male gender*	*66*	*OR, 1.178 (1.034, 1.341)*	*1732*	***0.014***	*13.983 (0.174)*	*0.7810, 1.7760*	*Yes*	*No/No*	*1.178*	*IV*
Urbanicity	4	OR, 1.548 (0.584, 4.104)	59	0.380	69.435 (0.020)	−5.0916, 8.1876	Yes	Yes/No	1.548	ns
Age	61	SMD, −0.035 (−0.102, 0.033)	1776	0.313	32.012 (0.009)	−0.3260, 0.2560	No	No/No	0.939	ns
Parental socioeconomic status	14	OR, 0.955 (0.739, 1.234)	444	0.725	37.519 (0.077)	−0.2389, 2.1489	No	No/No	0.955	ns
Migrant status	2	OR, 0.932 (0.544, 1.596)	113	0.797	33.457 (0.220)	N/A	No	N/A/No	0.932	ns
Non-white ethnicity	19	OR, 0.949 (0.604, 1.203)	714	0.665	25.641 (0.143)	−0.4990, 1.8070	No	No/No	0.949	ns
Education	25	OR, 0.872 (0.718, 1.059)	795	0.167	40.038 (0.021)	−0.4570, 1.6630	No	No/No	0.872	ns

*Note*: *K*, number of samples for each factor,; ES, effect size; *N*, number of cases; PI, prediction interval; CI, confidence interval; SSE, small study effect; ESB, excess significance bias; LS, largest study with significant effect; eOR, equivalent odds ratio; CE, class of evidence; OR, odds ratio; SMD, standardized mean difference; NA, not assessable; ns, not significant. Higher classes of evidence for associations are emphasized in italics. Bold text is indicative of why factors are not a higher CE.

**Table 2.** Level of Evidence for the Association of Later Factors and Psychotic Disorders

Risk Factor	*K*	Random Effects Measures, ES (95% CI)	*N*	*P* Random Effects	*I* ^2^ (*P*)	PI	LS	SSE/ESB	eOR	CE
*Stress/trauma*	*11*	*OR, 1.146 (1.038, 1.265)*	***454***	*<10* ^*−6*^	*35.681 (0.113)*	*0.9015, 1.3905*	*No*	*No/No*	*1.146*	*IV*
*Living status*	*10*	*OR, 1.557 (1.085, 2.232)*	***289***	***0.016***	*0.000 (0.537)*	*0.6547, 2.4593*	*No*	*No/No*	*1.557*	*IV*
*Employment*	*7*	*OR, 0.553 (0.400, 0.765)*	***268***	*<10* ^*−4*^	*0.000 (0.870)*	*0.4000, 0.7650*	*No*	*No/No*	*0.553*	*IV*
Stigma	2	OR, 4.604 (0.825, 25.701)	21	0.082	70.619 (0.065)	N/A	Yes	N/A/Yes	4.604	ns
Substance misuse^a^	12	OR, 1.322 (0.965, 1.813)	382	0.082	13.760 (0.310)	0.1734, 2.4706	No	No/No	1.322	ns
Tobacco use	10	OR, 1.285 (0.904, 1.826)	233	0.162	14.907 (0.306)	0.0342, 2.5358	No	No/No	1.285	ns
Cannabis use	23	OR, 1.189 (0.954, 1.480)	759	0.123	35.848 (0.046)	0.0217, 2.3563	No	Yes/No	1.189	ns
Brain injury	2	OR, 0.888 (0.561, 1.405)	104	0.611	0.000 (0.665)	N/A	No	N/A/No	0.888	ns
Alcohol	10	OR, 0.834 (0.626, 1.110)	472	0.212	29.747 (0.171)	−0.3278, 1.9958	Yes	No/No	0.834	ns

*Note*: Abbreviations are explained in the first footnote to table 1. Higher classes of evidence for associations are emphasized in italics. Bold text is indicative of why factors are not a higher CE.

^a^Substance misuse refers to substances not covered by other factors i.e. does not refer to alcohol, cannabis, or tobacco use.

**Table 3.** Level of Evidence for the Association of Antecedents and Psychotic Disorders

Risk Factor	*K*	Random Effects Measures, ES (95% CI)	*N*	*P* Random Effects	*I* ^2^ (*P*)	PI	LS	SSE/ESB	eOR	CE
*Right handedness*	*16*	*OR, 1.602 (1.041, 2.465)*	***354***	***0.032***	*0.000 (0.663)*	*0.6576, 2.5464*	*No*	*No/No*	*1.602*	*IV*
Perinatal complications	6	OR, 2.058 (0.893, 4.746)	129	0.090	87.785 (0.000)	−2.5694, 6.6854	Yes	No/No	2.058	ns
Height	5	SMD, 0.157 (−0.047, 0.361)	138	0.132	0.000 (0.824)	−0.1742, 0.4882	No	Yes/No	1.329	ns
BMI	3	SMD, −-0.060 (−0.440, 0.320)	26	0.756	0.000 (0.709)	−2.5234, 2.4034	No	Yes/No	0.897	ns

*Note*: Abbreviations are explained in the first footnote to table 1. BMI, body mass index. Higher classes of evidence for associations are emphasized in italics. Bold text is indicative of why factors are not a higher CE.

**Table 4.** Level of Evidence for the Association of Symptom Scores/Clinical Factors and Psychotic Disorders

Risk Factor	*K*	Random Effects Measures, ES (95% CI)	*N*	*P* Random Effects	*I* ^2^ (*P*)	PI	LS	SSE/ESB	eOR	CE
*Attenuated positive psychotic symptoms*	*49*	*SMD, 0.348 (0.280, 0.415)*	*1163*	*<10* ^*−6*^	***69.344 (<0.001)***	***−0.0010***, ***0.6970***	*Yes*	***Yes*** */No*	*2.563*	*II*
*Global functioning*	*49*	*SMD, −0.291 (−0.370, −0.211)*	*1560*	*<10* ^*−6*^	***76.205 (<0.001)***	***−0.7146, 0.1330***	*Yes*	***Yes*** */No*	*0.590*	*II*
*Negative psychotic symptoms*	*49*	*SMD, 0.393 (0.317, 0.469)*	*1374*	*<10* ^*−6*^	*62.872 (<0.001)*	***−*** *0.0090, 0.7770*	***No***	*Yes/No*	*2.681*	*III*
*Disorganized/cognitive symptoms*	*18*	*SMD, 0.317 (0.172, 0.461)*	***503***	*<10* ^*−6*^	*77.067 (<0.001)*	***−*** *0.1810, 0.8150*	*No*	*Yes/No*	*2.485*	*IV*
*Total symptoms score*	*29*	*SMD, 0.307 (0.148, 0.467)*	***675***	*<10* ^*−6*^	*72.282 (<0.001)*	***−*** *0.4403, 1.0543*	*No*	*Yes/No*	*1.743*	*IV*
*General symptoms*	*21*	*SMD, 0.227 (0.122, 0.332)*	***541***	*<10* ^*−4*^	*62.307 (<0.001)*	***−*** *0.1190, 0.5730*	*No*	*Yes/No*	*2.271*	*IV*
Co-morbidity	19	OR, 1.134 (0.926, 1.389)	587	0.223	54.470 (0.002)	0.4282, 1.8392	No	No/No	1.134	ns
Basic symptoms	2	SMD, 0.267 (−0.027, 0.562)	115	0.075	43.119 (0.185)	N/A	Yes	N/A/No	1.621	ns

*Note*: Abbreviations are explained in the first footnote to table 1.

Higher classes of evidence for associations are emphasized in italics. Bold text is indicative of why factors are not a higher CE.

### Convincing Evidence for Association With Transition to Psychosis

**Fig. 2.**
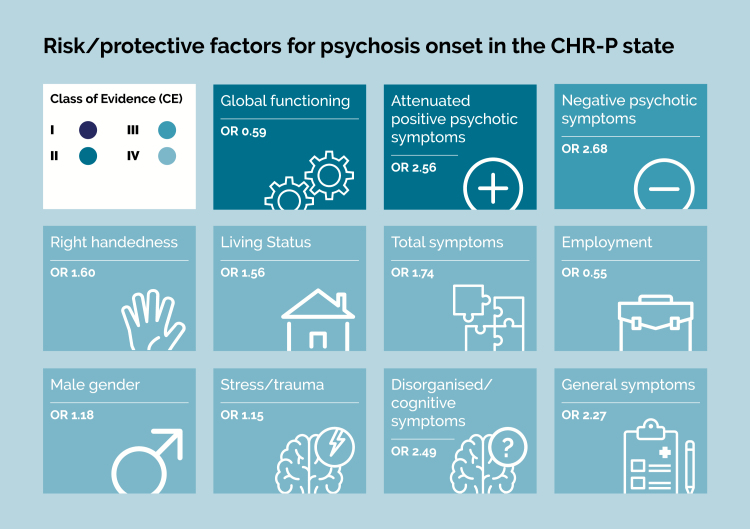
Graphical summary of risk/protective factors for psychosis onset in the CHR-P state. No factors met criteria for a convincing level of evidence (class I), two factors for a highly suggestive level of evidence (class II), one factor for a suggestive level of evidence (class III) and eight for a weak level of evidence (class IV).

There were no risk or protective factors with a convincing level of evidence (class I: number of cases >1000, *P* < 10^−6^, *I*^2^ < 50%, 95% prediction interval excluding the null, no small‐study effects, and no excess significance bias) for an association with risk of transition to psychosis (tables 1–4, figure 2).

### Highly Suggestive Evidence for Association With Transition to Psychosis

There was highly suggestive evidence (class II: >1000 cases, *P* < 0.001, largest study with statistically significant effect, and class I criteria not met) that two further factors are associated with increased (attenuated positive psychotic symptoms; SMD = 0.348, 95% CI: 0.280, 0.415) and decreased (global functioning; SMD = −0.291, 95% CI: −0.370, −0.211) transition risk, respectively (table 4, figure 2).

### Suggestive Evidence for Association With Transition to Psychosis

There was suggestive evidence (class III: >1000 cases, *P* < 0.01, class I/II criteria not met) for negative psychotic symptoms (SMD = 0.393, 95% CI: 0.317, 0.469) increasing risk of transition to psychosis (table 4, figure 2). This changed little when analyses were run without total negative SIPS/SOPS scores or questionable negative SIPS/SOPS items (SMD = 0.369, 95% CI: 0.280, 0.458)

### Weak Evidence of Association With Transition to Psychosis

There was weak evidence (class IV: *P* < 0.05 and class I–III criteria not met) of an association with increased risk of transition to psychosis for one sociodemographic/parental factor (male gender [OR = 1.178, 95% CI: 1.034, 1.341]), three later factors (stress/trauma [OR = 1.146, 95% CI: 1.038, 1.265], living status [OR = 1.557, 95% CI: 1.085, 2.232], employment [OR = 0.553, 95% CI: 0.400, 0.765]), one antecedent (right handedness [OR = 1.602, 95% CI: 1.041, 2.465]), and three symptom scores/clinical factors (disorganized/cognitive symptoms [SMD = 0.317, 95% CI: 0.172, 0.461], general symptoms [SMD = 0.227, 95% CI: 0.122, 0.332], total symptom scores [SMD = 0.307, 95% CI: 0.148, 0.467]) (tables 1–4, figure 2).

There was no evidence of association with transition to psychotic disorders for all other 16 factors (see tables 1–4, figure 2)

### No Change in Classification of Evidence of Associations After Sensitivity Analysis

No factors with suggestive evidence or greater (attenuated positive psychotic symptoms, global functioning, and negative psychotic symptoms) were downgraded following removal of studies with retrospective designs (supplementary table 6) or studies not using *ICD* or *DSM* criteria to determine transition status in addition to CHR-P instruments. Only one study was considered to have a retrospective design with all other studies having prospective designs.

## Discussion

To the best of our knowledge, this is the first meta-analysis of risk and protective factors for transition to psychotic disorders in CHR-P individuals that includes a robust hierarchical classification of the published evidence. After two decades of CHR-P research, it was imperative to advance knowledge by screening the available evidence against robust criteria. Overall, 128 individual studies comprising 17 967 patients and 26 factors potentially associated with transition to psychosis were included. There were no factors with convincing evidence (class I) for an association with risk of transition. Attenuated positive psychotic symptoms and global functioning were characterized by highly suggestive evidence (class II) with negative psychotic symptoms supported by suggestive evidence (class III).

The main finding of this meta-analysis is that, although a large number of risk/protective factors for transition to psychotic disorders have been evaluated in numerous CHR-P studies, none show convincing evidence with few having suggestive or stronger support. This likely reflects a research field which is fragmented, heterogeneous and that still represents a small niche to display a scalable impact. For example, the availability of different CHR-P assessment instruments is associated with disagreement in the designation of cases or definition of their outcomes.^32^ The recent introduction of the *DSM-5* category of Attenuated Psychosis Syndrome has further complicated the psychometric comparability of CHR-P cases.^33^ On a similar note, the heterogeneity of the CHR-P group has already been demonstrated at both diagnostic^32,34^ and prognostic^14,35^ level, to the point that stratification of this group has been suggested in a previous issue of this journal.^1,36^ The limited scalability and impact of the CHR-P field has also received empirical demonstration on several lines of evidence. Since the CHR-P literature is characterized by relatively small studies with infrequent events (transition to psychosis), the meta-analytical findings did not survive the strict criteria for the classification of evidence, with it being particularly rare for factors to have more than 1000 cases. Although this criterion is intended to identify robust epidemiological risk/protective factors, the CHR-P field is per se epidemiologically weak,^37^ because it is characterized by substantial sampling biases.^7^ A striking example of these points is the recent evidence showing that only about 5% of individuals who will develop psychosis can be detected at their CHR-P stage in secondary mental health care.^38,39^ Overall, this finding clearly indicates that future CHR-P research should be collaborative, scalable, and better harmonized in terms of assessment of intake criteria and outcomes. Ongoing international projects such as PSYSCAN,^40^ PRONIA^41^, and North American Prodrome Longitudinal Study (NAPLS)^42^ which have all been integrated in the HARMONY project may reach the critical mass that is needed to better identify risk/protective factors that modulate transition to psychosis with convincing evidence.

Despite these caveats, we found highly suggestive evidence that attenuated positive psychotic symptoms and global functioning are directly and inversely associated with the risk of transitioning to psychosis, respectively. These findings are unsurprising. First, severity of positive symptoms is the main factor in deciding whether an individual meets CHR-P criteria and develops a first episode of psychosis. CHR-P individuals with higher attenuated positive psychotic symptom scores at baseline are closer to the threshold of transition and therefore do not require the same degree of symptom progression as others with less severe symptoms. Although there is consensus that current CHR-P tools are biased toward detecting attenuated positive psychotic symptoms^1,43,44^ the P1-P4 subscales on the CAARMS and the P1-P5 subscales on the SIPS actually contain a variety of attenuated symptoms beyond positive ones.^1,32^ For example, obsessive thoughts, derealization and depersonalization experiences as well as time perception alterations. Fine-grained data are not available: most studies did not report the single severity and frequency scores of the specific CAARMS/SIPS subscales (see supplementary table 3). Moreover, this is true of randomized controlled trials.^43^ When these data were available, sensitivity analyses confirmed that all individual attenuated positive psychotic symptoms remained significant with the exception of grandiose ideas (supplementary table 4). Moreover, even when splitting attenuated positive psychotic symptoms into individual items on assessments, this may not be fine-grained enough for optimal prediction. Previous important studies have shown that auditory hallucinations may be highly predictive of transition to psychosis, whereas visual hallucinations may be associated with a reduced risk.^45^ Unfortunately, this level of detail in data is rarely reported in primary literature, so further analysis was not possible. Second, previous research has already shown that higher functioning at baseline is associated with reduced risk of transition.^46,47^ Although impaired global functioning is variably ascertained by CHR-P assessment instruments,^32^ it is one of the most robust predictors in this field. Machine-learning prediction models determined social outcomes at 1 year in up to 83% of patients in clinical high-risk states and 70% of patients with recent-onset depression.^48^ We also found suggestive evidence for a direct association of negative psychotic symptom severity and risk of transition to psychosis. This factor would have met the criteria for highly suggestive evidence, however, the largest study^49^ did not show a statistically significant effect. Negative psychotic symptoms of at least moderate severity are incredibly common among CHR-P individuals with 82% endorsing at least one negative psychotic symptom^50^ and with high prevalence (41%) of comorbid affective disorders.^51^ Negative psychotic symptoms along with impaired baseline global functioning, are typically the driving force for individuals seeking help at CHR-P services^52^ and their persistence leads to poor outcomes.^53^

A number of other factors were found to have weak evidence of an association with transition to psychosis in CHR-P individuals, with the key restriction for a greater class of evidence being fewer than 1000 cases. Stress/trauma increased risk of psychosis within CHR-P individuals. Our previous meta-analyses found that trauma is a key risk factor for psychosis in the general population^12^ and a risk factor for CHR-P status.^13^ Male gender was also seen to increase psychosis risk within CHR-P samples. Our previous meta-analyses found it to be a risk factor for psychosis in the general population^12^ and for CHR-P status,^13^ however with greater effect sizes than in this analysis. One potential explanation for this lies in the fact that the current analysis focuses on an enriched sample for these factors, thus diluting the variance. This is likely to be true of other factors traditionally associated with psychosis, such as cannabis use, that were found to have non-significant associations in this analysis. Moreover, cannabis use has typically been assessed in a binary fashion, measuring if individuals have ever used before, despite degree of exposure seemingly being key to the association with psychosis in both the general population^54^ and in CHR-P^55^. We also found that employment is protective, reducing the risk of transitioning to psychosis in CHR-P individuals. Employment is an indirect measure, contingent on other factors such as symptoms and global functioning.^56^ Right handedness also had a weak association with increased psychosis risk within CHR-P individuals. This effect was in the opposite direction to in the general population.^12^ However, as many included studies were fairly small and data was not systematically reported, interpretation should be taken cautiously. Other clinical factors, particularly disorganized and cognitive symptoms, were found to have a weak association with psychosis. Their impact can be particularly relevant within the clinical subgroup of brief limited intermittent psychotic symptoms (BLIPS), where disorganizing or dangerous features have been associated with an extremely high risk of transition.^34^

The above findings can advance clinical knowledge in this area. First, they can be used to improve the prognosis of outcomes. At present, CHR-P assessment tools have outstanding sensitivity but lack specificity^6,8^, ie they are adept at ruling out psychosis risk but are inefficient at ruling it in. Accordingly, recent studies have suggested using refined clinical prediction models to improve prognostic accuracy.^38,39,57^ The risk and protective factors identified in class II and III of evidence in the current meta-analysis could represent core benchmarks for developing future clinical prediction models. Some of the factors identified by our analysis have already been incorporated into risk calculators for CHR-P individuals. For example, the NAPLS calculator includes higher levels of attenuated positive psychotic symptoms (unusual thought content and suspiciousness) and greater decline in social functioning,^57^ whereas another calculator similarly includes attenuated positive psychotic symptoms (unusual thought content, visual perceptual abnormalities, and disorganization), negative psychotic symptoms (social anhedonia and ideational richness) and global functioning.^58^ Prognostic accuracy can be further improved when clinical prediction models are combined with biomarker^59^ or cognitive^57^ data in a sequential assessment framework. Stepped assessments offer the advantage to optimize the resources reserving more complex assessment to those already filtered through simpler procedures.^25^ Our analysis also reveals key risk/protective factors that at the moment present with weak evidence for association and that awaits further validation through larger cohort studies. Improved understanding of which CHR-P individuals are more likely to transition to psychosis would also lead to some potential clinical benefits such as easiest detection of those more at risk and faster referrals to early detection services, thereby reducing the duration of untreated psychosis and improving outcomes.^60–67^ Finally, advancing knowledge on factors that modulate the onset of psychosis within CHR-P samples can inform preventive interventions, as some of these may be potentially modifiable. Available preventive interventions do not seem to be more effective, compared to each other, nor benefit the severity of attenuated positive psychotic symptoms,^43^ negative psychotic symptoms^68^, or global functioning^69^ that have been identified as class II–III. Although the meta-analytical picture is currently bleak, due to the infancy of the field there have been very few randomized controlled trials in CHR-P. Further studies and increased focus on the effects of these treatments on the severity of attenuated positive psychotic symptoms, negative symptoms, and global functioning are key to the progression of the field. Since there is no evidence that current preventive treatments can reliably modify the risk of developing psychosis in CHR-P samples,^70^ experimental therapeutics in this area are urgently needed and should be the focus of the next generation of research.

The main limitation of the current analysis is that the CHR-P literature is still relatively small compared to other areas of psychiatry. For example, our umbrella review^12^ assessing risk and protective factors for psychosis was able to draw on 50 years of evidence, and yet was only able to find two factors with a convincing level of evidence. Although the CHR-P field only has the past 20 years to draw evidence from, there are still two factors with highly suggestive levels of evidence. This may be due to the fact that the CHR-P paradigm is intrinsically embedded in prospective cohort studies. Future studies in this area have the potential to move class III factors into higher classes and therefore to progress and improve the evidence base. Similarly, as already noted in the umbrella review,^12^ the vast majority of factors assessed in the current literature are risk factors, rather than protective factors. Protective factors like self-esteem, social support, and resilience may be better assessed in future primary research studies to identify what may aid psychosis prevention. Another limitation is the clinical heterogeneity of the CHR-P population. Within this, there are people with attenuated psychotic symptoms (APS), BLIPS and genetic risk and deterioration syndrome (GRD).^14^ Furthermore, there are differences between APSS (attenuated positive symptom syndrome) as defined by the SIPS, APS as defined by the CAARMS, *DSM-5* APS, as well as others.^33^ For example, within SIPS-based studies, the decline of global functioning is intrinsically linked to the GRD subgroup only. However, these differences are limited at the meta-analytical level^8^ with the majority of risk (around 60%) for developing psychosis in CHR-P individuals being accumulated before the assessment is performed.^7,71^ As such, there is a high degree of variance within the CHR-P cohorts in these studies, which can dilute the effect of certain risk factors as they can affect these subgroups differently. Future studies would be wise to subdivide and sufficiently power their samples to ascertain the differential effects of risk/protective factors on these subgroups.^1^ Finally, there were only a few studies available to contribute data for some factors, and as for any other meta-analysis that has adopted our classification criteria of evidence, we cannot exclude that other risk/protective factors may be identified and published in the near future.

## Conclusions

Severity of attenuated positive psychotic symptoms and low global functioning show convincing evidence, whereas severity of negative psychotic symptoms shows suggestive evidence for increasing transition risk in CHR-P individuals. These factors should be considered as benchmarks by future clinical prediction models and key targets of new experimental therapeutics.

## Funding

D.O. is supported by the UK Medical Research Council (MR/N013700/1) and King’s College London member of the MRC Doctoral Training Partnership in Biomedical Sciences.

## Supplementary Material

sbz039_suppl_Supplementary-Material
