# Supplementary material for: What Causes the Onset of Psychosis in Individuals at Clinical High Risk? A Meta-analysis of Risk and Protective Factors
Source: Schizophr Bull. 2019 Jun 20;46(1):110–20. doi: 10.1093/schbul/sbz039 (PMC6942149; doi:10.1093/schbul/sbz039)
Supplement: sbz039_suppl_Supplementary-Material [file sbz039_suppl_supplementary-material.docx]

**Supplementary Online Content**

Oliver D, Reilly T, Baccaredda Boy O et al. What causes the onset of psychosis in individuals at clinical high risk? A meta-analysis of risk and protective factors.

**eMethods 1** – Search keywords

**eMethods 2 –** Extracted variables

**eMethods 3 -** Risk of bias (quality) assessment

**eTable 1** – MOOSE guidelines for meta-analysis and systematic reviews

**eTable 2** – PRISMA guidelines for meta-analysis and systematic reviews

**eTable 3** – Factors examined by study

**eTable 4 –** Sensitivity analyses of attenuated positive psychotic symptoms at the symptom level

**eTable 5 –** Scales used to assess attenuated positive psychotic symptoms, negative psychotic symptoms and global functioning

**eTable 6 -** Sensitivity analysis for the associations of symptom scores/clinical factors and psychotic disorders within individual prospective studies of class I‐III factors

**eTable 7** - Risk of bias (quality assessment) using modified Newcastle Ottawa Scale

**eFigure1-48** – Funnel plot of standard error/precision against standardised mean difference/log odds ratio

**eReferences**

**eMethods 1 – Search keywords**

1. Psychosis OR psychotic OR schizo*
2. Risk OR prodrom* UHR OR CHR OR ARMS
3. subclinic* subthreshold* OR develop* OR future OR imminent OR incipient OR onset OR earl*) adj2 (psychosis OR psychotic OR schizo*
4. 1 AND (2 OR 3)

The above search was combined with keywords relating to risk factors for psychosis which have shown significant associations in the literature previously identified by our group^12^:

Pregnan* OR Obstetr* OR Pre-eclampsia OR Preeclampsia OR Placent* OR Rhesus OR Premature OR Pre-mature OR Smok* OR Tobacco OR Obes* OR Diabet* OR Cannabis OR Utero OR Uterine OR Perinatal OR Prenatal OR Fetus OR Fetal OR Chlamydia OR HHV* OR Herpes OR Toxoplasma OR VZV OR Varicella Zoster OR EBV OR Epstein Barr vir* OR CMV OR Cytomegalovir* OR BDV OR Borna disease vir* OR HERV* OR Endogenous retrovirus* OR Influenza* OR HTLV* OR Human T-lymphotropic virus OR JC vir* OR John Cunningham vir* OR BK vir* OR HIV OR Human Immunodeficiency Virus OR AIDS OR Acquired Immune Deficiency OR Toxocara OR Parvovir* OR Epilep* OR Seizure* OR CNS adj2 infect* OR (Central nervous system) adj2 infect* OR Brain adj2 injur* OR Trauma* OR Life adj2 event OR Stress OR Advers* OR Abus* OR Neglect* OR Bully* OR Parent* adj2 loss OR Parent* adj2 separat* OR Social adj2 class OR Adopt* OR Migra* OR Immigr* OR Emigr* OR Environment* OR Urban* OR Season adj2 (birth OR born) OR Educat* OR Relig* OR Cultur* OR Physical adj2 activ* OR Exercis* OR Diet* OR Risk* adj2 factor* OR Protect*

**eMethods 2 – Extracted variables**

Several variables were recorded: year of publication, lead author, study setting, study design, definition of psychosis risk and instrument used, length of follow-up, definition of transition to psychosis, type of factor investigated, number of individuals in the exposed group, number of individuals in the unexposed group, number and risk of transition to psychosis in the exposed group at each time-point (Outcome), number and risk of transition to psychosis in the unexposed group at each time-point (Outcome), demographics of the exposed and unexposed group (age, gender, ethnicity), medication use in the exposed and unexposed group, type of treatments received in the exposed and unexposed group

**eMethods 3 - Risk of bias (quality) assessment**

Bias was assessed using a modified version of the Newcastle-Ottawa Scale for cohort studies, in line with our previous studies^13,29^. Studies were awarded a maximum of eight points on items related to the selection of the cohort, comparability of exposed and non-exposed groups, ascertainment of exposure, ascertainment of outcome, and adequacy of follow-up. This was scored independently by two investigators (T.R. & O.B.B.) with disagreement resolved in consensus meetings with another author (D.O.).

| **eTable 1:** MOOSE guidelines for meta-analysis and systematic reviews | | **Reported on page #** |
| --- | --- | --- |
| **Title** | Identify the study as a meta-analysis (or systematic review) | **1** |
| **Abstract** | Use the journal’s structured format | **3** |
| **Introduction** | **Present**   - The clinical problem - The hypothesis - A statement of objectives that includes the study population, the condition of interest, the exposure or intervention, and the outcome(s) considered | - **5** - **5** - **5** |
| **Sources** | **Describe**   - - Qualifications of searchers (e.g. librarians and investigators)   - Search strategy, including time period included in the synthesis and keywords   - Effort to include all available studies, including contact with authors   - Databases and registries searched   - Search software used, name and version, including special features used (e.g. explosion)   - Use of hand searching (e.g. reference lists of obtained articles)   - List of citations located and those excluded, including justification   - Method of addressing articles published in languages other than English   - Method of handling abstracts and unpublished studies   - Description of any contact with authors | - - **6**   - **6**   - **6**   - **6**   - **6**   - **6**   - **e7**   - **7**   - **7**   - **n/a** |
| **Study Selection** | **Describe**   - Types of study designs considered - Relevance or appropriateness of studies gathered for assessing the hypothesis to be tested - Rationale for the selection and coding of data (e.g. sound clinical principles or convenience) - Documentation of how data were classified and coded (e.g. multiple raters, blinding and interrater reliability) - Assessment of confounding (e.g. comparability of cases and controls in studies where appropriate) - Assessment of study quality, including blinding of quality assessors; stratification or regression on possible predictors of study results - Assessment of heterogeneity - Statistical methods (e.g. complete description of fixed or random effects models, justification of whether the chosen models account for predictors of study results, dose-response models, or cumulative meta-analysis) in sufficient detail to be replicated) | - **7** - **7** - **7/8** - **7/8** - **8** - **8** - **9** - **8-10** |
| **Results** | **Present**   - A graph summarizing individual study estimates and the overall estimate - A table giving descriptive information for each included study - Results of sensitivity testing (eg, subgroup analysis) - Indication of statistical uncertainty of findings | - **n/a** - **e7** - **23-26** - **11** |
| **Discussion** | **Discuss**   - Strengths and weaknesses - Potential biases in the review process (eg, publication bias) - Justification for exclusion (eg, exclusion of non–English-language citations) - Assessment of quality of included studies - Consideration of alternative explanations for observed results - Generalization of the conclusions (ie, appropriate for the data presented and within the domain of the literature review) - Guidelines for future research - Disclosure of funding source | - **12-13, 15-17** - **13** - **16** - **e13** - **12-15** - **17** - **15/16** - **18** |

| **eTable 2:** PRISMA guidelines for meta-analysis and systematic reviews | | | |
| --- | --- | --- | --- |
| **Section/topic** |   **#** | **Checklist item** |      **Page** |
| **TITLE** | | |  |
| Title |   1 | Identify the report as a systematic review, meta-analysis, or both. | 1 |
| **ABSTRACT** | | |  |
| Structured summary | 2 | Provide a structured summary including, as applicable: background; objectives; data sources; study eligibility criteria, participants, and interventions; study appraisal and synthesis methods; results; limitations; conclusions and implications of key findings; systematic review registration number. | 3 |
| **INTRODUCTION** | | |  |
| Rationale | 3 | Describe the rationale for the review in the context of what is already known. | 4/5 |
| Objectives | 4 | Provide an explicit statement of questions being addressed with reference to participants, interventions, comparisons, outcomes, and study design (PICOS). | 5 |
| **METHODS** | | |  |
| Protocol and registration |   5 | Indicate if a review protocol exists, if and where it can be accessed (e.g., Web address), and, if available, provide registration information including registration number. | 6 |
| Eligibility criteria | 6 | Specify study characteristics (e.g., PICOS, length of follow-up) and report characteristics (e.g., years considered, language, publication status) used as criteria for eligibility, giving rationale. | 7 |
| Information sources | 7 | Describe all information sources (e.g., databases with dates of coverage, contact with study authors to identify additional studies) in the search and date last searched. | 6 |
| Search | 8 | Present full electronic search strategy for at least one database, including any limits used, such that it could be repeated. | 6 |
| Study selection | 9 | State the process for selecting studies (i.e., screening, eligibility, included in systematic review, and, if applicable, included in the meta-analysis). | 5-6 |
| Data collection process | 10 | Describe method of data extraction from reports (e.g., piloted forms, independently, in duplicate) and any processes for obtaining and confirming data from investigators. | 7/8 |
| Data items | 11 | List and define all variables for which data were sought (e.g., PICOS, funding sources) and any assumptions and simplifications made. | 7/8 |
| Risk of bias in individual studies | 12 | Describe methods used for assessing risk of bias of individual studies (including specification of whether this was done at the study or outcome level), and how this information is to be used in any data synthesis. | 8 |
| Summary measures | 13 | State the principal summary measures (e.g., risk ratio, difference in means). | 8 |
| Synthesis of results | 14 | Describe the methods of handling data and combining results of studies, if done, including measures of consistency (e.g., I2) for each meta-analysis. | 8/9 |
| Risk of bias across studies | 15 | Specify any assessment of risk of bias that may affect the cumulative evidence (e.g., publication bias, selective reporting within studies). | 9/10 |
| Additional analyses | 16 | Describe methods of additional analyses (e.g., sensitivity or subgroup analyses, meta-regression), if done, indicating which were pre-specified. | 9/10 |
| **RESULTS** |  |  |  |
| Study selection | 17 | Give numbers of studies screened, assessed for eligibility, and included in the review, with reasons for exclusions at each stage, ideally with a flow diagram. | 23 |
| Study characteristics | 18 | For each study, present characteristics for which data were extracted (e.g., study size, PICOS, follow-up period) and provide the citations. | e6-e11 |
| Risk of bias within studies | 19 | Present data on risk of bias of each study and, if available, any outcome level assessment (see item 12). | e13-e18 |
| Results of individual studies | 20 | For all outcomes considered (benefits or harms), present, for each study: (a) simple summary data for each intervention group (b) effect estimates and confidence intervals, ideally with a forest plot. | - |
| Synthesis of results | 21 | Present results of each meta-analysis done, including confidence intervals and measures of consistency. | 24-28 |
| Risk of bias across studies | 22 | Present results of any assessment of risk of bias across studies (see Item 15). | 24-28 |
| Additional analysis | 23 | Give results of additional analyses, if done (e.g., sensitivity or subgroup analyses, meta-regression [see Item 16]). | 24-28, e12 |
| **DISCUSSION** |  |  |  |
| Summary of evidence |   24 | Summarize the main findings including the strength of evidence for each main outcome; consider their relevance to key groups (e.g., healthcare providers, users, and policy makers). | 12/13 |
| Limitations | 25 | Discuss limitations at study and outcome level (e.g., risk of bias), and at review-level (e.g., incomplete retrieval of identified research, reporting bias). | 16/17 |
| Conclusions | 26 | Provide a general interpretation of the results in the context of other evidence, and implications for future research. | 12-17 |
| **FUNDING** |  |  |  |
| Funding | 27 | Describe sources of funding for the systematic review and other support (e.g., supply of data); role of funders for the systematic review. | 18 |

| **eTable 3 – Factors examined by study** | | |
| --- | --- | --- |
| **Study** | **CHR-P Instrument** | **Factors examined** |
| Addington et al, 2017a^1^ | SIPS | Alcohol, Age, Attenuated positive psychotic symptoms*, Cannabis use, Functioning, Substance misuse |
| Addington et al, 2017b^2^ | SIPS | Co-morbidity |
| Alderman et al, 2015^3^ | SIPS | Non-white ethnicity |
| Allott et al, 2014^4^ | PANSS | Age, Attenuated positive psychotic symptoms, Co-morbidity, Education, Global functioning, Male gender, Negative psychotic symptoms, Substance misuse, Total attenuated psychotic symptoms score |
| Armando et al, 2015^5^ | SIPS | Age, Education, Global functioning, Male gender, Urbanicity |
| Atkinson et al, 2017^6^ | CAARMS | Alcohol, Cannabis use, Co-morbidity, Global functioning, Living status, Male gender, Right handedness, Substance misuse, Tobacco use, Unemployment |
| Auther et al, 2012^7^ | SOPS | Cannabis use |
| Auther et al, 2015^8^ | SIPS | Alcohol, Cannabis use |
| Bang et al, 2017^9^ | SIPS | Basic symptoms |
| Bearden et al, 2011^10^ | SIPS | Age, Global functioning, Male gender, Non-white ethnicity, Parental socioeconomic status, Total attenuated psychotic symptoms score |
| Bechdolf et al, 2011^11^ | CAARMS | Age, Co-morbidity, Global functioning, Male gender, Stress/trauma |
| Bedi et al, 2015^12^ | SIPS | Age, Male gender, Non-white ethnicity |
| Berger et al, 2016^13^ | PANSS | Alcohol, Age, Attenuated positive psychotic symptoms, Cannabis use, Male gender, Negative psychotic symptoms, Tobacco use |
| Bloemen et al, 2010^14^ | SIPS | Age, Cannabis use, Male gender, Right handedness |
| Bodatsch et al, 2011^15^ | ERIraos | Age, Education, Male gender, Right handedness, Tobacco use |
| Bousman et al, 2013^16^ | CAARMS | Age, Cannabis use, Global functioning |
| Brewer et al, 2003^17^ | BPRS | Right handedness, Tobacco use |
| Burcato et al, 2017^128^ | SIPS | Age, Attenuated positive psychotic symptoms, Disorganised/cognitive symptoms, Education, Ethnicity, Gender, General symptoms, Global functioning and Negative psychotic symptoms |
| Buchy et al, 2014^18^ | SIPS | Alcohol, Age, Cannabis use, Education, Living status, Male gender, Non-white ethnicity, Substance misuse, Tobacco use |
| Buchy et al, 2015^19^ | SIPS | Alcohol, Cannabis use, Male gender, Tobacco use |
| Cannon et al, 2008^20^ | SIPS | Attenuated positive psychotic symptoms*, Global functioning, Substance misuse |
| Cannon et al, 2016^21^ | SIPS | Attenuated positive psychotic symptoms*, Global functioning, Stress/trauma |
| Cotter et al, 2017^22^ | CAARMS | Unemployment |
| Chung et al, 2015^23^ | SIPS | Age, Attenuated positive psychotic symptoms*, Male gender, Non-white ethnicity, Parental socioeconomic status |
| Corcoran et al, 2015^24^ | SIPS | Age, Attenuated positive psychotic symptoms*, Male gender, Negative psychotic symptoms |
| Cornblatt et al, 2015^25^ | SIPS | Disorganised/cognitive symptoms, Global functioning |
| Crump et al, 2017^26^ | SIPS | Attenuated positive psychotic symptoms*, Male gender, Non-white ethnicity |
| Dazzan et al, 2012^27^ | BPRS/CAARMS | Male gender, Right handedness |
| Deighton et al, 2016^28^ | SIPS | Cannabis use |
| Demjaha et al, 2012^29^ | CAARMS | Disorganised/cognitive symptoms, Negative psychotic symptoms |
| DeVylder et al, 2014^30^ | SIPS | Age, Attenuated positive psychotic symptoms*, Cannabis use, Education, Male gender, Negative psychotic symptoms, Non-white ethnicity, Stress/trauma |
| Dragt et al, 2011^31^ | SIPS | Age, Brain injury, Cannabis use, Global functioning, Male gender, Non-white ethnicity, Parental socioeconomic status, Perinatal complications, Unemployment, Urbanicity |
| Focking et al, 2016^32^ | PANSS | Alcohol, Age, BMI, Cannabis use, Male gender, Tobacco use |
| Francey et al, 2005^33^ | BPRS | Global functioning, Male gender |
| Fusar-Poli et al, 2010^34^ | PACE/CAARMS | Living status, Unemployment |
| Garner et al, 2005^35^ | BPRS | Age, Male gender, Negative psychotic symptoms, Total attenuated psychotic symptoms score |
| Grivel et al, 2018^36^ |  | Stress/trauma |
| Gschwandtner et al, 2009^37^ | BSIP | Total attenuated psychotic symptoms score |
| Haroun et al, 2006^38^ | SIPS | Age, Attenuated positive psychotic symptoms, Co-morbidity, Disorganised/cognitive symptoms, Education, Global functioning, General symptoms, Male gender, Negative psychotic symptoms, Substance misuse, Total attenuated psychotic symptoms score |
| Healey et al, 2013^39^ | SIPS | Education, Non-white ethnicity, Parental socioeconomic status |
| Heinze et al, 2015^40^ | CAARMS | Age, Attenuated positive psychotic symptoms*, Male gender, Total attenuated psychotic symptoms score |
| Hengartner et al, 2017^41^ | SIPS | Attenuated positive psychotic symptoms*, Global functioning, Negative psychotic symptoms |
| Higuchi et al, 2013^42^ | CAARMS | Age, Attenuated positive psychotic symptoms, Male gender, Negative psychotic symptoms |
| Hur et al, 2015^43^ | CAARMS/SIPS | Parental socioeconomic status |
| Ising et al, 2016^44^ | CAARMS | Attenuated positive psychotic symptoms, General symptoms, Global functioning, Negative psychotic symptoms |
| Keefe et al, 2006^45^ | SIPS | Non-white ethnicity |
| Kim et al, 2011^46^ | SIPS | Age, Attenuated positive psychotic symptoms, Education, General symptoms, Global functioning, Male gender, Negative psychotic symptoms, Parental socioeconomic status, Right handedness, Total attenuated psychotic symptoms score |
| Korkeila et al, 2013^47^ | SIPS | Co-morbidity, Living status, Male gender, Perinatal complications, |
| Kotlicka-Antczak et al, 2017^48^ | CAARMS | Age, Attenuated positive psychotic symptoms, Co-morbidity, Education, Male gender, Negative psychotic symptoms, Perinatal complications, Substance misuse, Tobacco, Total attenuated psychotic symptoms score |
| Koutsouleris et al, 2009^49^ | PANSS | Age, Attenuated positive psychotic symptoms, Education, General symptoms, Male gender, Negative psychotic symptoms, Right handedness, Perinatal complications, Total attenuated psychotic symptoms score |
| Koutsouleris et al, 2012a^50^ | PANSS | Right handedness |
| Koutsouleris et al, 2012b^51^ | PANSS | Age, Attenuated positive psychotic symptoms, Education, General symptoms, Global functioning, Male gender, Negative psychotic symptoms, Total attenuated psychotic symptoms score |
| Kraan et al, 2015^52^ | CAARMS | Stress/trauma |
| Kraan et al, 2017^53^ | CAARMS | Stress/trauma |
| Kraan et al, 2017b^54^ | CAARMS | Stress/trauma |
| Kristensen et al, 2007^55^ | SIPS | Cannabis use |
| Kwon et al, 2012^56^ | CAARMS/SIPS | Age, Male gender, Total attenuated psychotic symptoms score |
| Labad et al, 2015^57^ | CAARMS | Alcohol, Age, Attenuated positive psychotic symptoms, BMI, Cannabis use, General symptoms, Male gender, Negative psychotic symptoms, Stress/trauma, Tobacco use |
| Lavoie et al, 2012^58^ | PANSS | Age, Attenuated positive psychotic symptoms, Male gender, Negative psychotic symptoms |
| Lavoie et al, 2014^59^ | PANSS | Alcohol, Age, Cannabis use, Male gender, Right handedness |
| Lee et al, 2014^60^ | SIPS | Male gender |
| Lehembre-Shiah et al, 2017^61^ | SIPS | Age, Attenuated positive psychotic symptoms*, Disorganised/cognitive symptoms, General symptoms, Global functioning, Negative psychotic symptoms, Non-white ethnicity |
| Lemos-Giraldez et al, 2009^62^ | SIPS | Age, Attenuated positive psychotic symptoms, Disorganised/cognitive symptoms, Education, General symptoms, Global functioning, Male gender, Negative psychotic symptoms, Substance misuse, Total attenuated psychotic symptoms score |
| Li et al, 2017^63^ | SIPS | Global functioning, Total attenuated psychotic symptoms score |
| Lim et al, 2015^64^ | CAARMS | Co-morbidity |
| Lim et al, 2016^65^ | SIPS | Attenuated positive psychotic symptoms, Co-morbidity, Disorganised/cognitive symptoms, General symptoms, Global functioning, Negative psychotic symptoms |
| Lin et al, 2013^66^ | CAARMS | Age, Attenuated positive psychotic symptoms*, Education, Global functioning, Male gender, Negative psychotic symptoms |
| Lyngberg et al, 2015^67^ | SIPS | Global functioning |
| Mason et al, 2004^68^ | BPRS | Attenuated positive psychotic symptoms, Negative psychotic symptoms |
| McAusland et al, 2017^69^ | SIPS | Co-morbidity |
| McHugh et al, 2017^69^ | CAARMS | Cannabis use |
| McLaughlin et al, 2016^70^ | SIPS | Age, Attenuated positive psychotic symptoms, Global functioning |
| Mechelli et al, 2017^71^ | CAARMS | Attenuated positive psychotic symptoms*, Basic symptoms, Disorganised/cognitive symptoms, General symptoms, Global functioning, Negative psychotic symptoms, |
| Metzler et al, 2016^72^ | SIPS | Age, Attenuated positive psychotic symptoms, Global functioning, Male gender, Negative psychotic symptoms |
| Michel et al, 2014^73^ | SIPS | Age, Education, Living status, Male gender, Unemployment |
| Mittal et al, 2007^74^ | SIPS | Age, General symptoms, Male gender, Non-white ethnicity, Total attenuated psychotic symptoms score |
| Mittal et al, 2009^75^ | SIPS | Non-white ethnicity, Perinatal complications |
| Mittal et al, 2010a^76^ | SIPS | Attenuated positive psychotic symptoms, Co-morbidity, General symptoms, Negative psychotic symptoms, Total attenuated psychotic symptoms score |
| Mittal et al, 2010b^77^ | SIPS | Age, Male gender, Parental socioeconomic status |
| Mourik et al, 2017^78^ | SIPS | Age, Attenuated positive psychotic symptoms, Disorganised/cognitive symptoms, Education, General symptoms, Global functioning, Male gender, Negative psychotic symptoms, |
| Nelson et al, 2012^79^ | CAARMS | Disorganised/cognitive symptoms |
| Nieman et al, 2009^80^ | SIPS | Age, Global functioning, Male gender |
| Nieman et al, 2013^81^ | SIPS | Symptomatology, Total attenuated psychotic symptoms score |
| Nieman et al, 2014^82^ | SIPS | Age, Cannabis use, Global functioning, Male gender, Negative psychotic symptoms, Urbanicity |
| O’Donoghue et al, 2015^83^ | CAARMS | Global functioning, Parental socioeconomic status |
| Papmeyer et al, 2016^84^ | BSIP | Age, Education, General symptoms, Living status, Male gender, Stress/trauma, Substance misuse, Total attenuated psychotic symptoms score |
| Pawelczyk et al, 2016^85^ | CAARMS | Age, Attenuated positive psychotic symptoms*, BMI, Education, Global functioning, Male gender, Parental socioeconomic status, Tobacco use, Urbanicity |
| Perez et al, 2014^86^ | SIPS | Age, Attenuated positive psychotic symptoms, Male gender, Negative psychotic symptoms, Parental socioeconomic status, Right handedness |
| Phillips et al, 2002a^87^ | BPRS | Cannabis use |
| Phillips et al, 2002b^88^ | BPRS | Age, Height, Male gender, Right handedness |
| Piskulic et al, 2012^89^ | SIPS | Negative psychotic symptoms |
| Power et al, 2016^90^ | CAARMS | Male gender |
| Ramyead et al, 2015^91^ | SIPS | Age, Attenuated positive psychotic symptoms, Co-morbidity, Education, Male gender, Negative psychotic symptoms, Substance misuse, Total attenuated psychotic symptoms score |
| Rekhi et al, 2017^92^ | CAARMS | Age, General symptoms, Global functioning, Male gender |
| Riecher-Rossler et al, 2009^93^ | BSIP | Attenuated positive psychotic symptoms, Cannabis use, Co-morbidity, Disorganised/cognitive symptoms, Global functioning, Negative psychotic symptoms, Substance misuse |
| Ruhrmann et al, 2010^94^ | SIPS | Attenuated positive psychotic symptoms*, Co-morbidity, Education, General symptoms |
| Rusch et al, 2015^95^ | SIPS | Attenuated positive psychotic symptoms, Age, Global functioning, Male gender, Negative psychotic symptoms, Stigma |
| Sabb et al, 2010^96^ | SIPS | Global functioning, Right handedness |
| Salokangas et al, 2012^97^ | SIPS | Co-morbidity, Stigma |
| Salokangas et al, 2013^98^ | SIPS | Attenuated positive psychotic symptoms, Disorganised/cognitive symptoms, Negative psychotic symptoms, Total attenuated psychotic symptoms score |
| Schultze-Lutter et al, 2014^99^ | SIPS | Age, Co-morbidity, Global functioning, Living status, Male gender, Unemployment |
| Seidman et al, 2010^100^ | SIPS | Education, Non-white ethnicity, Parental socioeconomic status |
| Seidman et al, 2016^101^ | SIPS | Alcohol, Age, Cannabis use, Education, Male gender, Negative psychotic symptoms, Non-white ethnicity, Parental socioeconomic status |
| Simeonova et al, 2011^102^ | SIPS | Disorganised/cognitive symptoms, General symptoms, Global functioning, Negative psychotic symptoms, Non-white ethnicity |
| Stowkowy et al, 2016^103^ | SIPS | Stress/trauma |
| Takahashi et al, 2010^104^ | CAARMS | Age, Height, Male gender, Negative psychotic symptoms, Right handedness, Total attenuated psychotic symptoms score |
| Takahashi et al, 2014^105^ | CAARMS | Age, Attenuated positive psychotic symptoms, Height, Male gender, Negative psychotic symptoms, Right handedness, Total attenuated psychotic symptoms score |
| Tamagni et al, 2013^106^ | BSIP | Co-morbidity |
| Tarbox et al, 2013^107^ | SIPS | Age, Attenuated positive psychotic symptoms*, Education, Global functioning, Male gender, Negative psychotic symptoms, Non-white ethnicity, Parental socioeconomic status |
| Thompson et al, 2011^108^ | CAARMS | Age, Attenuated positive psychotic symptoms*, Education, Global functioning, Male gender, Non-white ethnicity, Substance misuse, Total attenuated psychotic symptoms score |
| Thompson et al, 2014^109^ | BPRS/CAARMS | Stress/trauma |
| Valmaggia et al, 2014^110^ | BPRS/CAARMS | Cannabis use |
| van Tricht et al, 2010^111^ | SIPS | Age, Male gender |
| van Tricht et al, 2014^112^ | SIPS | Age, Male gender |
| Velthorst et al, 2009^113^ | SIPS | Age, Attenuated positive psychotic symptoms, Cannabis use, Co-morbidity, Disorganised/cognitive symptoms, General symptoms, Global functioning, Male gender, Negative psychotic symptoms |
| Velthorst et al, 2010^114^ | SIPS | Global functioning |
| Velthorst et al, 2013^115^ | CAARMS | Age, Attenuated positive psychotic symptoms, Global functioning, Male gender, Negative psychotic symptoms, Total attenuated psychotic symptoms score |
| Walder et al, 2013^116^ | SIPS | Age, Attenuated positive psychotic symptoms, Disorganised/cognitive symptoms, Global functioning, Male gender, Negative psychotic symptoms |
| Walker et al, 2010^117^ | SIPS | Negative psychotic symptoms |
| Walterfang et al, 2008^118^ | BPRS | Age, Height, Male gender, Negative psychotic symptoms, Right handedness, Total attenuated psychotic symptoms score |
| Yun et al, 2005^119^ | BPRS | Age, Global functioning, Male gender, Negative psychotic symptoms, Perinatal complications, Total attenuated psychotic symptoms score |
| Yung et al, 1998^120^ | BPRS | Attenuated positive psychotic symptoms, Global functioning, Negative psychotic symptoms, Total attenuated psychotic symptoms score |
| Yung et al, 2004^121^ | BPRS | Negative psychotic symptoms |
| Yung et al, 2005^122^ | CAARMS | Attenuated positive psychotic symptoms*, Disorganised/cognitive symptoms, General symptoms, Negative psychotic symptoms, Total attenuated psychotic symptoms score |
| Zhang et al, 2015^123^ | SIPS | Age |
| Zhang et al, 2017^124^ | SIPS | Age, Attenuated positive psychotic symptoms*, Co-morbidity, Disorganised/cognitive symptoms, Education, Global functioning, General symptoms, Living status, Male gender, Negative psychotic symptoms, Total attenuated psychotic symptoms score, Unemployment |
| Ziermans et al, 2011^125^ | SIPS | Cannabis use, Living status |
| Ziermans et al, 2012^126^ | SIPS | Height, Right handedness, Total attenuated psychotic symptoms score |
| Ziermans et al, 2014^127^ | SIPS | Age, Attenuated positive psychotic symptoms, Disorganised/cognitive symptoms, General symptoms, Global functioning, Male gender, Negative psychotic symptoms, Parental socioeconomic status |
| BPRS – Brief Psychiatric Rating Scale, BSIP – Basel Screening Instrument for Psychosis, CAARMS – Comprehensive Assessment for at Risk Mental States, ERIraos - Early Recognition Inventory based on the retrospective assessment of the onset of schizophrenia, SIPS – Structured Interview for Prodromal Syndromes  *Reported data for individual item scores for positive subscales of CAARMS/SIPS for sensitivity analysis  Overlapping study samples were removed from the analysis | | |

| **eTable 4 – Sensitivity analyses of attenuated positive psychotic symptoms at the symptom level** | | | | | |
| --- | --- | --- | --- | --- | --- |
| **Factor** | **K** | **Random effects measures, ES (95% CI)** | **N** | **P random effects** | **I^2^ (p)** |
| Conceptual disorganisation | 4 | SMD, 0.274 (0.086, 0.462) | 251 | 0.004 | 23.818 (0.268) |
| Disorders of thought content | 3 | SMD, 0.525 (0.358, 0.692) | 231 | <0.001 | 0.119 (0.367) |
| Disorganised communication | 8 | SMD, 0.483 (0.347, 0.619) | 389 | <0.001 | 0.000 (0.991) |
| Grandiose ideas | 7 | SMD, 0.036 (0.237, 0.350) | 374 | 0.727 | 58.610 (0.025) |
| Perceptual abnormalities | 12 | SMD, 0.155 (0.057, 0.252) | 565 | 0.002 | 0.000 (0.610) |
| Suspiciousness/Paranoia | 9 | SMD, 0.316 (0.135, 0.497) | 441 | 0.001 | 61.231 (0.008) |
| Unusual thought content | 14 | SMD, 0.384 (0.300, 0.468) | 666 | <0.001 | 0.000 (0.468) |

| **eTable 5 – Scales used to assess attenuated positive psychotic symptoms, negative psychotic symptoms and global functioning** | | | |
| --- | --- | --- | --- |
| **Study** | **Attenuated positive psychotic symptoms** | **Negative psychotic symptoms** | **Global functioning** |
| Addington et al, 2017a^1^ | SIPS | N/A | QLS, SFS |
| Allott et al, 2014^4^ | PANSS | MADRS, PANSS | GAF |
| Armando et al, 2015^5^ | N/A | N/A | GF: Role, GF: Social |
| Atkinson et al, 2017^6^ | N/A | N/A | GAF, GF: Role, GF: Social, SOFAS |
| Bearden et al, 2011^10^ | N/A | N/A | GAF |
| Bechdolf et al, 2011^11^ | N/A | N/A | SOFAS |
| Berger et al, 2016^13^ | PANSS | PANSS | N/A |
| Bousman et al, 2013^16^ | N/A | N/A | GAF |
| Brucato et al, 2017^128^ | SIPS | SIPS | GF: Role, GF: Social |
| Cannon et al, 2008^20^ | SIPS | N/A | GF: Social |
| Cannon et al, 2016^21^ | SIPS | N/A | GF: Social |
| Chung et al, 2015^23^ | SIPS | N/A | N/A |
| Corcoran et al, 2015^24^ | SIPS | SOPS | N/A |
| Cornblatt et al, 2015^25^ | N/A | N/A | GAF |
| Crump et al, 2017^26^ | SIPS | N/A | N/A |
| Demjaha et al, 2012^29^ | N/A | CAARMS | N/A |
| DeVylder et al, 2014^30^ | SIPS | SOPS | N/A |
| Dragt et al, 2011^31^ | N/A | N/A | GAF |
| Francey et al, 2005^33^ | N/A | N/A | GAF |
| Garner et al, 2005^35^ | N/A | HRSD, SANS | N/A |
| Haroun et al, 2006^38^ | SAPS, SIPS | SANS, SIPS | GAF |
| Heinze et al, 2015^40^ | BPRS, CAARMS | N/A | N/A |
| Hengartner et al, 2017^41^ | SIPS | SIPS | GAF |
| Higuchi et al, 2013^42^ | CAARMS | SANS | N/A |
| Ising et al, 2016^44^ | CAARMS | CAARMS | PBIQ-R, SOFAS |
| Kim et al, 2011^46^ | SIPS | HRSD, PANSS | GAF |
| Kotlicka-Antczak et al, 2017^48^ | CAARMS | CAARMS | N/A |
| Koutsouleris et al, 2009^49^ | PANSS | MADRS, PANSS | N/A |
| Koutsouleris et al, 2012b^51^ | PANSS | MADRS, PANSS | GAF |
| Labad et al, 2015^57^ | CAARMS | PANSS | N/A |
| Lavoie et al, 2012^58^ | PANSS | PANSS | N/A |
| Lehembre-Shiah et al, 2017^61^ | SIPS | SIPS | GAF |
| Lemos-Giraldez et al, 2009^62^ | SIPS | SOPS | GAF |
| Li et al, 2017^63^ | N/A | N/A | GAF |
| Lim et al, 2016^65^ | SIPS | SOPS | GAF |
| Lin et al, 2013^66^ | CAARMS | HRSD, SANS | GAF |
| Lyngberg et al, 2015^67^ | N/A | N/A | PAS |
| Mason et al, 2004^68^ | BPRS | APSS, SANS | N/A |
| McLaughlin et al, 2016^70^ | SIPS | N/A | GAF, GF: Role |
| Mechelli et al, 2017^71^ | CAARMS | CAARMS, SANS | GAF |
| Metzler et al, 2016^72^ | SIPS | HRSD, PANSS | GAF |
| Mittal et al, 2010a^76^ | SIPS | SIPS | N/A |
| Mourik et al, 2017^78^ | SIPS | SIPS | GAF |
| Nieman et al, 2009^80^ | N/A | N/A | GAF |
| Nieman et al, 2014^82^ | N/A | SIPS | PAS |
| O’Donoghue et al, 2015^83^ | N/A | N/A | GAF |
| Pawelczyk et al, 2016^85^ | CAARMS | N/A | SOFAS |
| Perez et al, 2014^86^ | SIPS | SOPS | N/A |
| Piskulic et al, 2012^89^ | N/A | SIPS | N/A |
| Ramyead et al, 2015^91^ | SIPS | BPRS | N/A |
| Rekhi et al, 2017^92^ | N/A | N/A | SOFAS |
| Riecher-Rossler et al, 2009^93^ | BPRS, BSIP | BSIP, SANS | BSIP |
| Ruhrmann et al, 2010^94^ | SIPS | N/A | N/A |
| Rusch et al, 2015^95^ | SIPS | PANSS | GAF |
| Sabb et al, 2010^96^ | N/A | N/A | GAF |
| Salokangas et al, 2013^98^ | SPQ | SPQ | N/A |
| Schultze-Lutter et al, 2014^99^ | N/A | N/A | SOFAS |
| Seidman et al, 2016^101^ | N/A | CDS | N/A |
| Simeonova et al, 2011^102^ | N/A | CBCL | CBCL |
| Takahashi et al, 2010^104^ | N/A | SANS | N/A |
| Takahashi et al, 2014^105^ | BPRS | SANS | N/A |
| Tarbox et al, 2013^107^ | SIPS | SIPS | PAS |
| Velthorst et al, 2009^113^ | SOPS | SOPS | GAF |
| Velthorst et al, 2010^114^ | N/A | N/A | GAF, WHODAS |
| Velthorst et al, 2013^115^ | CAARMS | HDRS, SANS | GAF, QLS |
| Walder et al, 2013^116^ | SIPS | SOPS | GF: Role, GF: Social |
| Walker et al, 2010^117^ | N/A | BDI | N/A |
| Walterfang et al, 2008^118^ | N/A | SANS | N/A |
| Yun et al, 2005^119^ | N/A | SANS | GAF |
| Yung et al, 1998^120^ | BPRS | HDRS, SANS | QLS |
| Yung et al, 2004^121^ | N/A | SANS | N/A |
| Yung et al, 2005^122^ | CAARMS | CAARMS | N/A |
| Zhang et al, 2017^124^ | SIPS | SOPS | GAF |
| Ziermans et al, 2014^127^ | SIPS | SIPS | GAF |
| APSS – Assessment of Prodromal and Schizotypal Symptoms, BDI – Beck’s Depression Inventory, BPRS – Brief Psychiatric Rating Scale, BSIP – Basel Screening Instrument for Psychosis, CAARMS – Comprehensive Assessment for at Risk Mental States, CBCL – Child Behaviour Checklist, CDS – Calgary Depression Scale, ERIraos - Early Recognition Inventory based on the retrospective assessment of the onset of schizophrenia, GAF – Global Assessment of Functioning, GF: Role – Global Functioning Role Scale, GF: Social – Global Functioning Social Scale, HRSD – Hamilton Rating Scale for Depression, MADRS – Montgomery Asberg Depression Rating Scale, PAS – Premorbid Adjustment Scale, PBIQ-R – Personal Beliefs about Illness Questionnaire Revised, QLS – Quality of Life Scale, SANS – Scale for the Assessment of Negative Symptoms, SAPS – Scale for the Assessment of Positive Symptoms, SFS – Social and Functioning Scale, SIPS – Structured Interview for Prodromal Syndromes, SOFAS – Social and Occupational Functioning Assessment Scale, SOPS – Scale of Prodromal Symptoms, SPQ – Scale for the Assessment of Schizotypal Personality, WHODAS – World Health Organisation Disability Assessment Schedule | | | |

| **eTable 6 - Sensitivity analysis for the associations of symptom scores/clinical factors and psychotic disorders within individual prospective studies of class I‐III factors** | | | | | | | | | | |
| --- | --- | --- | --- | --- | --- | --- | --- | --- | --- | --- |
| **Risk factor** | **K** | **Random effects measures, ES (95% CI)** | **N** | **P random effects** | **I^2^ (p)** | **PI** | **LS** | **SSE/ESB** | **eOR** | **CE** |
| Attenuated positive psychotic symptoms | 47 | SMD, 0.363 (0.280, 0.446) | 1004 | <10^-6^ | 77.74 (<0.001) | -0.589 - 1.315 | Yes | Yes/No | 1.929 | II |
| Functioning | 47 | SMD, -0.290 (-0.391, -0.189) | 1425 | <10^-6^ | 75.993 (<0.001) | -1.207 - 0.627 | Yes | Yes/No | 1.690 | II |
| Negative psychotic symptoms | 47 | SMD, 0.416 (0.325, 0.507) | 1215 | <10^-6^ | 63.257 (<0.001) | -0.567 - 1.399 | No | Yes/No | 2.123 | III |
| k – number of samples for each factor, ES – effect size, N – number of cases, PI – prediction interval, CI – confidence interval, SSE – small study effect, ESB – excess significance bias, LS – largest study with significant effect, eOR – equivalent odds ratio, CE – class of evidence, OR – odds ratio, SMD – standardised mean difference, NA – not assessable, ns – not significant | | | | | | | | | | |

| **eTable 7 – Risk of bias (quality assessment) using modified Newcastle Ottawa Scale** | | | | | | | | |
| --- | --- | --- | --- | --- | --- | --- | --- | --- |
|  | **Selection** | | | | **Comparability** | **Outcome** | | **Total score** |
| **Study** | **Representativeness of exposed cohort** | **Representativeness of non-exposed cohort** | **Ascertainment of exposure** | **Demonstration that outcome of interest was not present at start of study** | **Comparability of cohorts on the basis of the design or analysis** | **Assessment of outcome** | **Adequacy of follow-up** |  |
| Addington et al, 2017a^1^ | **-** | **-** | **+** | **+** | **+ +** | **-** | **+** | 5 |
| Addington et al, 2017b^2^ | **+** | **+** | **+** | **+** | **+ +** | **-** | **-** | 6 |
| Alderman et al, 2015^3^ | **+** | **+** | **-** | **+** | **- -** | **-** | **-** | 3 |
| Allott et al, 2014^4^ | **-** | **-** | **+** | **-** | **+ -** | **+** | **+** | 4 |
| Armando et al, 2015^5^ | **+** | **+** | **+** | **+** | **+ +** | **-** | **+** | 7 |
| Atkinson et al, 2017^6^ | **+** | **+** | **+** | **+** | **+ -** | **-** | **-** | 5 |
| Auther et al, 2012^7^ | **+** | **+** | **+** | **+** | **+ +** | **-** | **+** | 7 |
| Auther et al, 2015^8^ | **+** | **+** | **+** | **+** | **+ +** | **-** | **+** | 7 |
| Bang et al, 2017^9^ | **+** | **+** | **+** | **-** | **+ -** | **+** | **-** | 5 |
| Bearden et al, 2011^10^ | **+** | **+** | **+** | **+** | **+ -** | **+** | **+** | 7 |
| Bechdolf et al, 2011^11^ | **+** | **+** | **+** | **+** | **+ +** | **-** | **+** | 7 |
| Bedi et al, 2015^12^ | **+** | **+** | **+** | **+** | **- -** | **-** | **+** | 5 |
| Berger et al, 2016^13^ | **+** | **+** | **+** | **+** | **- -** | **+** | **-** | 5 |
| Bloemen et al, 2010^14^ | **-** | **-** | **+** | **+** | **- -** | **-** | **+** | 3 |
| Bodatsch et al, 2011^15^ | **+** | **+** | **-** | **+** | **- -** | **-** | **-** | 3 |
| Bousman et al, 2013^16^ | **+** | **+** | **+** | **+** | **- -** | **-** | **+** | 5 |
| Brewer et al, 2003^17^ | **+** | **+** | **+** | **+** | **- -** | **-** | **-** | 4 |
| Brucato et al, 2017^128^ | **+** | **+** | **+** | **+** | **- -** | **+** | **-** | 5 |
| Buchy et al, 2014^18^ | **+** | **+** | **+** | **+** | **+ +** | **-** | **+** | 7 |
| Buchy et al, 2015^19^ | **+** | **+** | **+** | **+** | **+ -** | **-** | **-** | 5 |
| Cannon et al, 2008^20^ | **+** | **+** | **+** | **-** | **+ +** | **-** | **-** | 5 |
| Cannon et al, 2016^21^ | **+** | **+** | **+** | **+** | **+ +** | **+** | **+** | 8 |
| Cotter et al, 2017^22^ | **+** | **+** | **+** | **-** | **+ -** | **-** | **-** | 4 |
| Chung et al, 2015^23^ | **+** | **+** | **+** | **+** | **- -** | **-** | **-** | 4 |
| Corcoran et al, 2015^24^ | **+** | **+** | **+** | **+** | **- -** | **-** | **-** | 4 |
| Cornblatt et al, 2015^25^ | **+** | **+** | **+** | **+** | **+ +** | **-** | **+** | 7 |
| Crump et al, 2017^26^ | **+** | **+** | **+** | **-** | **- -** | **-** | **-** | 3 |
| Dazzan et al, 2012^27^ | **+** | **+** | **+** | **-** | **- -** | **-** | **-** | 3 |
| Deighton et al, 2016^28^ | **+** | **+** | **+** | **+** | **- -** | **-** | **+** | 5 |
| Demjaha et al, 2012^29^ | **+** | **+** | **+** | **-** | **- -** | **-** | **+** | 4 |
| DeVylder et al, 2014^30^ | **+** | **+** | **+** | **+** | **+ +** | **-** | **-** | 6 |
| Dragt et al, 2011^31^ | **+** | **+** | **+** | **+** | **+ +** | **-** | **-** | 6 |
| Focking et al, 2016^32^ | **+** | **+** | **+** | **+** | **- -** | **+** | **-** | 7 |
| Francey et al, 2005^33^ | **+** | **+** | **+** | **-** | **- -** | **+** | **+** | 5 |
| Fusar-Poli et al, 2010^34^ | **+** | **+** | **+** | **+** | **- -** | **+** | **-** | 5 |
| Garner et al, 2005^35^ | **+** | **+** | **+** | **+** | **- -** | **+** | **-** | 5 |
| Grivel et al, 2018^36^ | **+** | **+** | **+** | **-** | **- -** | **+** | **-** | 4 |
| Gschwandtner et al, 2009^37^ | **+** | **+** | **+** | **-** | **- -** | **+** | **-** | 4 |
| Haroun et al, 2006^38^ | **-** | **-** | **+** | **+** | **- -** | **-** | **-** | 2 |
| Healey et al, 2013^39^ | **-** | **-** | **+** | **+** | **+ -** | **-** | **+** | 4 |
| Heinze et al, 2015^40^ | **+** | **+** | **+** | **-** | **- -** | **-** | **-** | 3 |
| Hengartner et al, 2017^41^ | **+** | **+** | **+** | **+** | **+ -** | **+** | **-** | 6 |
| Higuchi et al, 2013^42^ | **-** | **-** | **+** | **-** | **- -** | **-** | **-** | 1 |
| Hur et al, 2015^43^ | **+** | **+** | **+** | **-** | **- -** | **-** | **-** | 3 |
| Ising et al, 2016^44^ | **+** | **+** | **+** | **+** | **+ +** | **-** | **+** | 7 |
| Keefe et al, 2006^45^ | **+** | **+** | **-** | **-** | **+ +** | **-** | **-** | 4 |
| Kim et al, 2011^46^ | **+** | **+** | **+** | **+** | **- -** | **+** | **-** | 5 |
| Korkeila et al, 2013^47^ | **+** | **+** | **+** | **+** | **- -** | **-** | **-** | 4 |
| Kotlicka-Antczak et al, 2017^48^ | **+** | **+** | **+** | **+** | **- -** | **+** | **+** | 6 |
| Koutsouleris et al, 2009^49^ | **+** | **+** | **+** | **+** | **- -** | **+** | **-** | 5 |
| Koutsouleris et al, 2012a^50^ | **+** | **+** | **+** | **-** | **- -** | **+** | **-** | 4 |
| Koutsouleris et al, 2012b^51^ | **-** | **-** | **+** | **+** | **- -** | **-** | **+** | 3 |
| Kraan et al, 2015^52^ | **-** | **-** | **+** | **+** | **+ +** | **-** | **-** | 4 |
| Kraan et al, 2017^53^ | **+** | **+** | **+** | **+** | **+ +** | **-** | **-** | 6 |
| Kraan et al, 2017b^54^ | **+** | **+** | **+** | **+** | **+ -** | **-** | **-** | 5 |
| Kristensen et al, 2007^55^ | **+** | **+** | **+** | **+** | **- -** | **-** | **-** | 4 |
| Kwon et al, 2012^56^ | **+** | **+** | **+** | **-** | **- -** | **-** | **+** | 4 |
| Labad et al, 2015^57^ | **+** | **+** | **+** | **-** | **+ +** | **+** | **-** | 6 |
| Lavoie et al, 2012^58^ | **+** | **+** | **+** | **+** | **- -** | **-** | **-** | 4 |
| Lavoie et al, 2014^59^ | **+** | **+** | **+** | **-** | **- -** | **-** | **+** | 4 |
| Lee et al, 2014^60^ | **+** | **+** | **+** | **+** | **+ +** | **-** | **-** | 6 |
| Lehembre-Shiah et al, 2017^61^ | **+** | **+** | **+** | **-** | **+ +** | **-** | **+** | 6 |
| Lemos-Giraldez et al, 2009^62^ | **+** | **+** | **+** | **-** | **+ +** | **+** | **-** | 6 |
| Li et al, 2017^63^ | **+** | **+** | **+** | **-** | **+ -** | **+** | **+** | 6 |
| Lim et al, 2015^64^ | **+** | **+** | **+** | **+** | **- -** | **+** | **+** | 6 |
| Lim et al, 2016^65^ | **+** | **+** | **+** | **+** | **+ +** | **+** | **+** | 8 |
| Lin et al, 2013^66^ | **+** | **+** | **+** | **+** | **+ +** | **+** | **-** | 7 |
| Lyngberg et al, 2015^67^ | **-** | **-** | **+** | **+** | **- -** | **-** | **-** | 2 |
| Mason et al, 2004^68^ | **+** | **+** | **+** | **-** | **+ +** | **+** | **-** | 6 |
| McAusland et al, 2017^69^ | **+** | **+** | **+** | **+** | **- -** | **-** | **-** | 4 |
| McHugh et al, 2017^69^ | **+** | **+** | **+** | **+** | **+ +** | **-** | **+** | 7 |
| McLaughlin et al, 2016^70^ | **+** | **+** | **+** | **+** | **+ +** | **-** | **+** | 7 |
| Mechelli et al, 2017^71^ | **-** | **-** | **+** | **-** | **+ +** | **+** | **-** | 4 |
| Metzler et al, 2016^72^ | **+** | **+** | **+** | **+** | **+ +** | **-** | **+** | 7 |
| Michel et al, 2014^73^ | **+** | **+** | **+** | **+** | **- -** | **-** | **-** | 4 |
| Mittal et al, 2007^74^ | **-** | **-** | **+** | **+** | **+ -** | **+** | **-** | 4 |
| Mittal et al, 2009^75^ | **+** | **+** | **+** | **+** | **- -** | **+** | **-** | 5 |
| Mittal et al, 2010a^76^ | **+** | **+** | **+** | **+** | **+ -** | **+** | **-** | 6 |
| Mittal et al, 2010b^77^ | **+** | **+** | **+** | **+** | **- -** | **+** | **-** | 5 |
| Mourik et al, 2017^78^ | **+** | **+** | **+** | **+** | **- -** | **-** | **+** | 5 |
| Nelson et al, 2012^79^ | **+** | **+** | **+** | **+** | **+ +** | **+** | **+** | 8 |
| Nieman et al, 2009^80^ | **+** | **+** | **+** | **+** | **- -** | **-** | **+** | 5 |
| Nieman et al, 2013^81^ | **+** | **+** | **+** | **+** | **+ -** | **+** | **-** | 6 |
| Nieman et al, 2014^82^ | **+** | **+** | **+** | **+** | **+ -** | **+** | **-** | 6 |
| O’Donoghue et al, 2015^83^ | **+** | **+** | **+** | **+** | **+ +** | **-** | **-** | 6 |
| Papmeyer et al, 2016^84^ | **+** | **+** | **+** | **+** | **+ +** | **-** | **-** | 6 |
| Pawelczyk et al, 2016^85^ | **+** | **+** | **+** | **-** | **- -** | **+** | **+** | 5 |
| Perez et al, 2014^86^ | **+** | **+** | **+** | **-** | **- -** | **-** | **+** | 4 |
| Phillips et al, 2002a^87^ | **+** | **+** | **+** | **+** | **- -** | **+** | **-** | 5 |
| Phillips et al, 2002b^88^ | **+** | **+** | **+** | **+** | **- -** | **+** | **+** | 6 |
| Piskulic et al, 2012^89^ | **+** | **+** | **+** | **+** | **- -** | **-** | **+** | 5 |
| Power et al, 2016^90^ | **+** | **+** | **+** | **+** | **+ -** | **+** | **+** | 7 |
| Ramyead et al, 2015^91^ | **+** | **+** | **-** | **-** | **- -** | **-** | **-** | 2 |
| Rekhi et al, 2017^92^ | **+** | **+** | **+** | **+** | **+ -** | **+** | **-** | 6 |
| Riecher-Rossler et al, 2009^93^ | **+** | **+** | **+** | **+** | **+ +** | **+** | **+** | 8 |
| Ruhrmann et al, 2010^94^ | **+** | **+** | **+** | **+** | **+ +** | **-** | **+** | 7 |
| Rusch et al, 2015^95^ | **+** | **+** | **+** | **+** | **+ +** | **-** | **+** | 7 |
| Sabb et al, 2010^96^ | **+** | **+** | **+** | **+** | **- -** | **+** | **-** | 5 |
| Salokangas et al, 2012^97^ | **+** | **+** | **+** | **+** | **+ -** | **+** | **-** | 6 |
| Salokangas et al, 2013^98^ | **+** | **+** | **+** | **+** | **+ -** | **-** | **-** | 5 |
| Schultze-Lutter et al, 2014^99^ | **+** | **+** | **+** | **+** | **- -** | **+** | **-** | 5 |
| Seidman et al, 2010^100^ | **+** | **+** | **+** | **+** | **- -** | **-** | **+** | 5 |
| Seidman et al, 2016^101^ | **+** | **+** | **+** | **+** | **+ +** | **-** | **+** | 7 |
| Simeonova et al, 2011^102^ | **+** | **+** | **+** | **+** | **+ +** | **-** | **-** | 6 |
| Stowkowy et al, 2016^103^ | **+** | **+** | **+** | **+** | **+ +** | **+** | **+** | 8 |
| Takahashi et al, 2010^104^ | **+** | **+** | **+** | **+** | **- -** | **+** | **-** | 5 |
| Takahashi et al, 2014^105^ | **+** | **+** | **+** | **+** | **- -** | **-** | **-** | 4 |
| Tamagni et al, 2013^106^ | **+** | **+** | **+** | **+** | **- -** | **+** | **-** | 5 |
| Tarbox et al, 2013^107^ | **+** | **+** | **+** | **-** | **+ +** | **-** | **-** | 5 |
| Thompson et al, 2011^108^ | **+** | **+** | **+** | **+** | **+ +** | **-** | **-** | 6 |
| Thompson et al, 2014^109^ | **+** | **+** | **+** | **+** | **+ +** | **-** | **-** | 6 |
| Valmaggia et al, 2014^110^ | **+** | **+** | **+** | **-** | **- -** | **-** | **-** | 3 |
| van Tricht et al, 2010^111^ | **+** | **+** | **+** | **+** | **- -** | **-** | **-** | 4 |
| van Tricht et al, 2014^112^ | **+** | **+** | **+** | **+** | **- -** | **-** | **-** | 4 |
| Velthorst et al, 2009^113^ | **+** | **+** | **+** | **+** | **+ +** | **-** | **+** | 7 |
| Velthorst et al, 2010^114^ | **+** | **+** | **+** | **+** | **+ +** | **+** | **-** | 7 |
| Velthorst et al, 2013^115^ | **+** | **+** | **+** | **+** | **+ +** | **+** | **-** | 7 |
| Walder et al, 2013^116^ | **+** | **+** | **+** | **+** | **+ +** | **+** | **-** | 7 |
| Walker et al, 2010^117^ | **-** | **-** | **+** | **+** | **- -** | **+** | **-** | 3 |
| Walterfang et al, 2008^118^ | **+** | **+** | **+** | **+** | **- -** | **-** | **-** | 4 |
| Yun et al, 2005^119^ | **+** | **+** | **+** | **+** | **- -** | **-** | **-** | 4 |
| Yung et al, 1998^120^ | **+** | **+** | **+** | **+** | **- -** | **-** | **-** | 4 |
| Yung et al, 2004^121^ | **+** | **+** | **+** | **+** | **+ +** | **-** | **+** | 7 |
| Yung et al, 2005^122^ | **+** | **+** | **+** | **-** | **- -** | **+** | **+** | 5 |
| Zhang et al, 2015^123^ | **+** | **+** | **+** | **-** | **- -** | **+** | **-** | 4 |
| Zhang et al, 2017^124^ | **+** | **+** | **+** | **-** | **+ +** | **+** | **-** | 6 |
| Ziermans et al, 2011^125^ | **+** | **+** | **+** | **+** | **- -** | **+** | **+** | 6 |
| Ziermans et al, 2012^126^ | **+** | **+** | **+** | **-** | **- -** | **+** | **-** | 4 |
| Ziermans et al, 2014^127^ | **+** | **+** | **+** | **+** | **+ -** | **+** | **-** | 6 |

eFigure 1 Funnel plot of standard error against standardised mean difference for Age

eFigure 2 Funnel plot of precision against standardised mean difference for Age


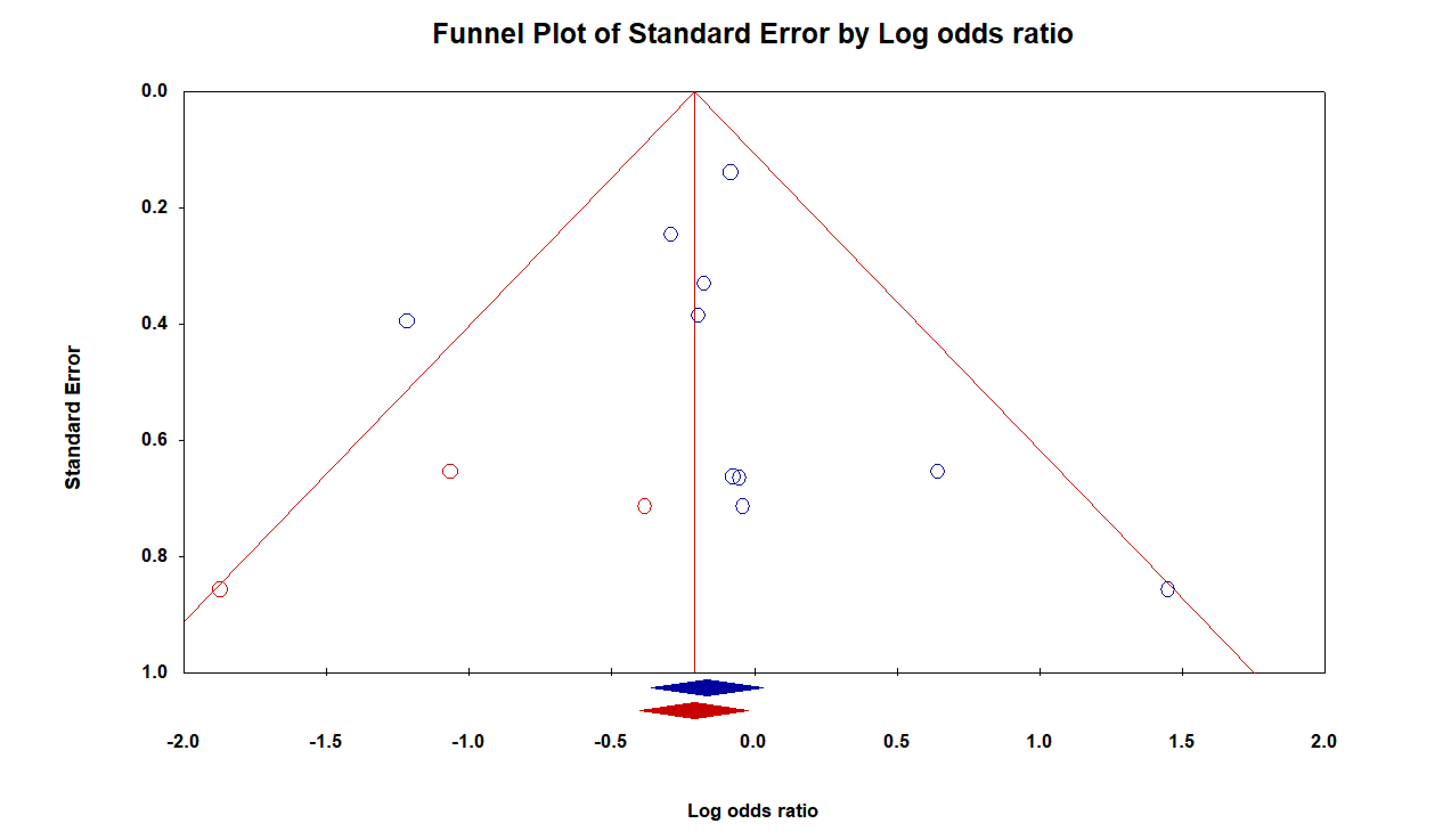


eFigure 3 Funnel plot of standard error against log odds ratio for Alcohol


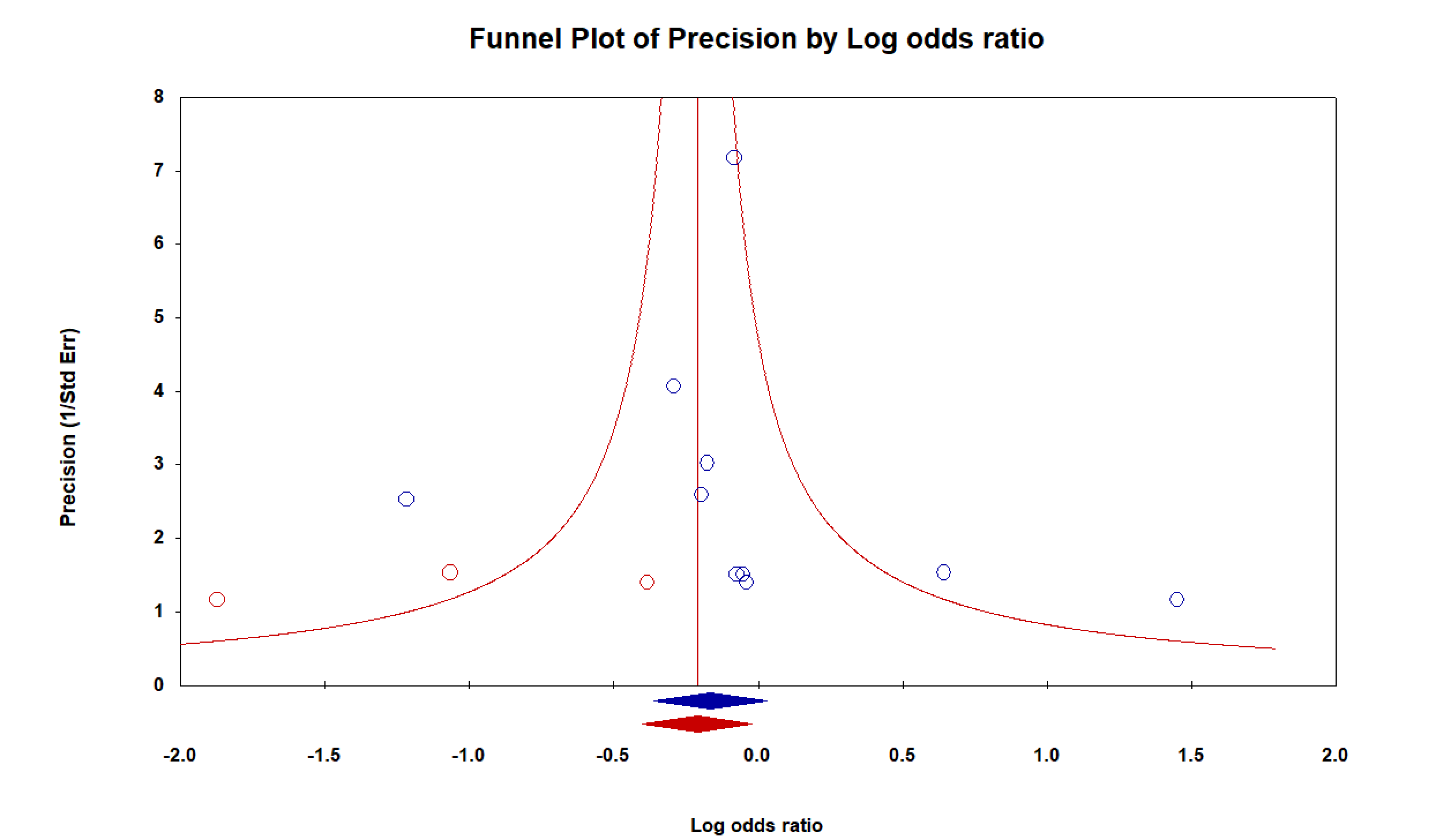


eFigure 4 Funnel plot of precision against log odds ratio for Alcohol


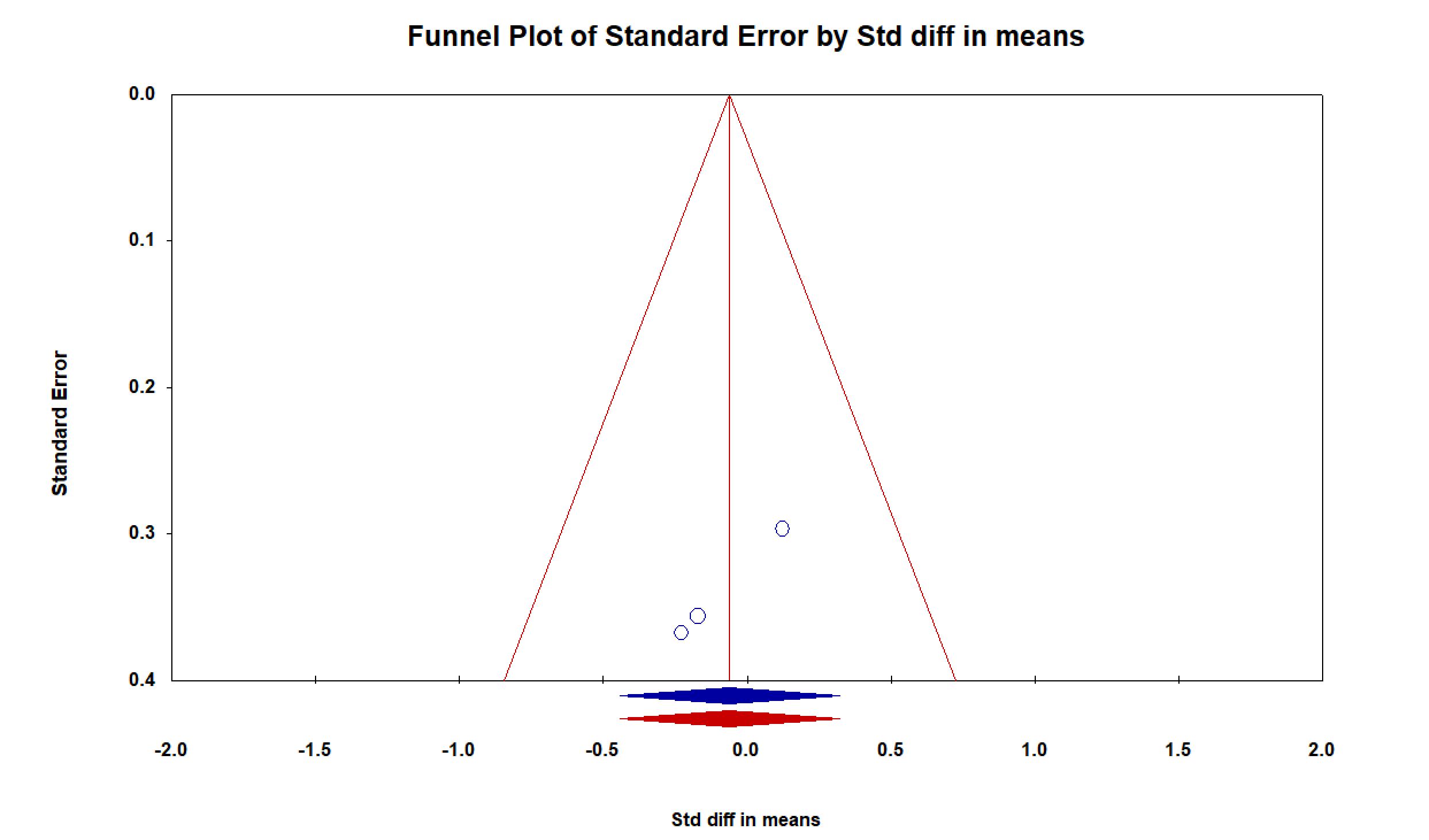


eFigure 5 Funnel plot of standard error against standardised mean difference for BMI


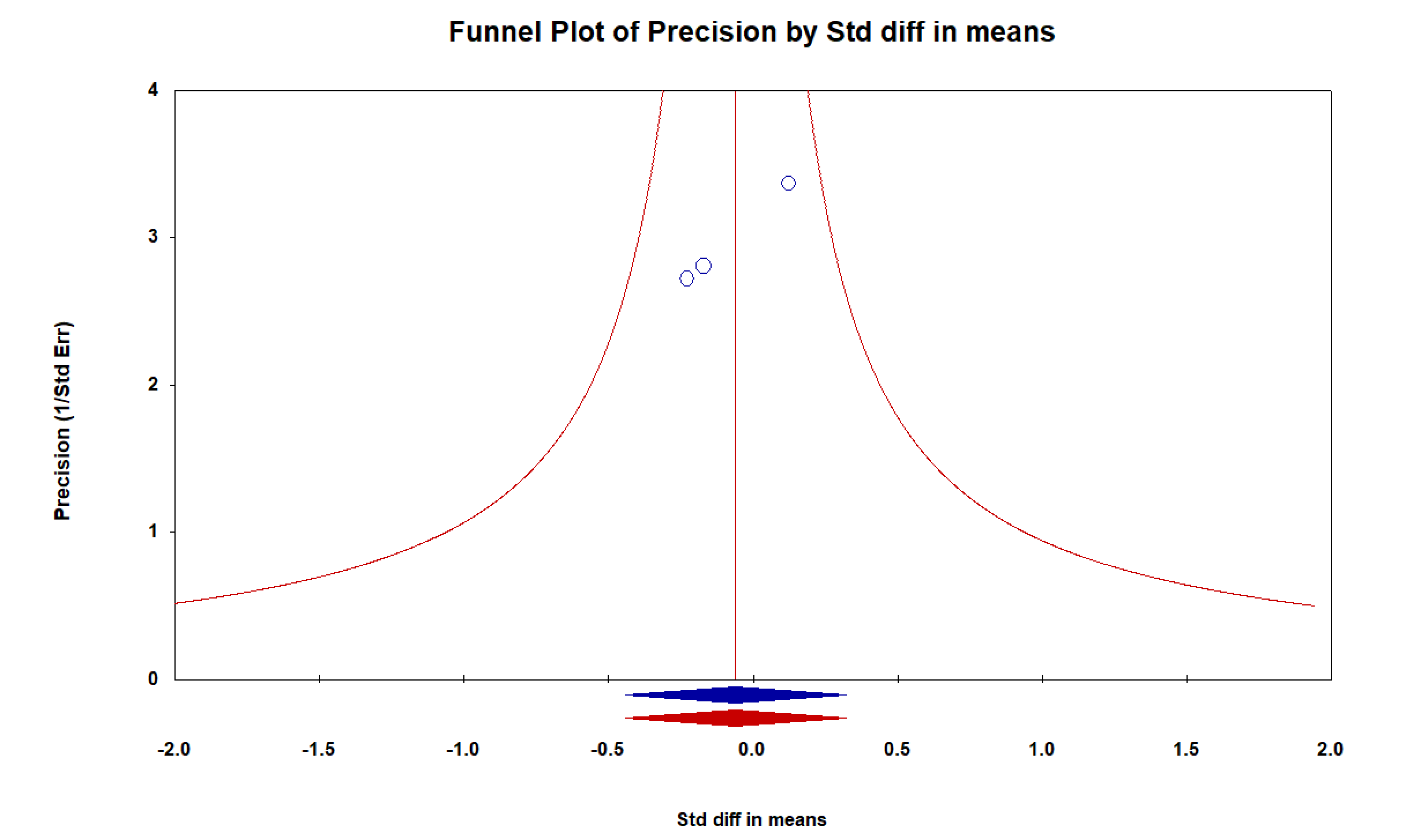


eFigure 6 Funnel plot of precision against standardised mean difference for BMI


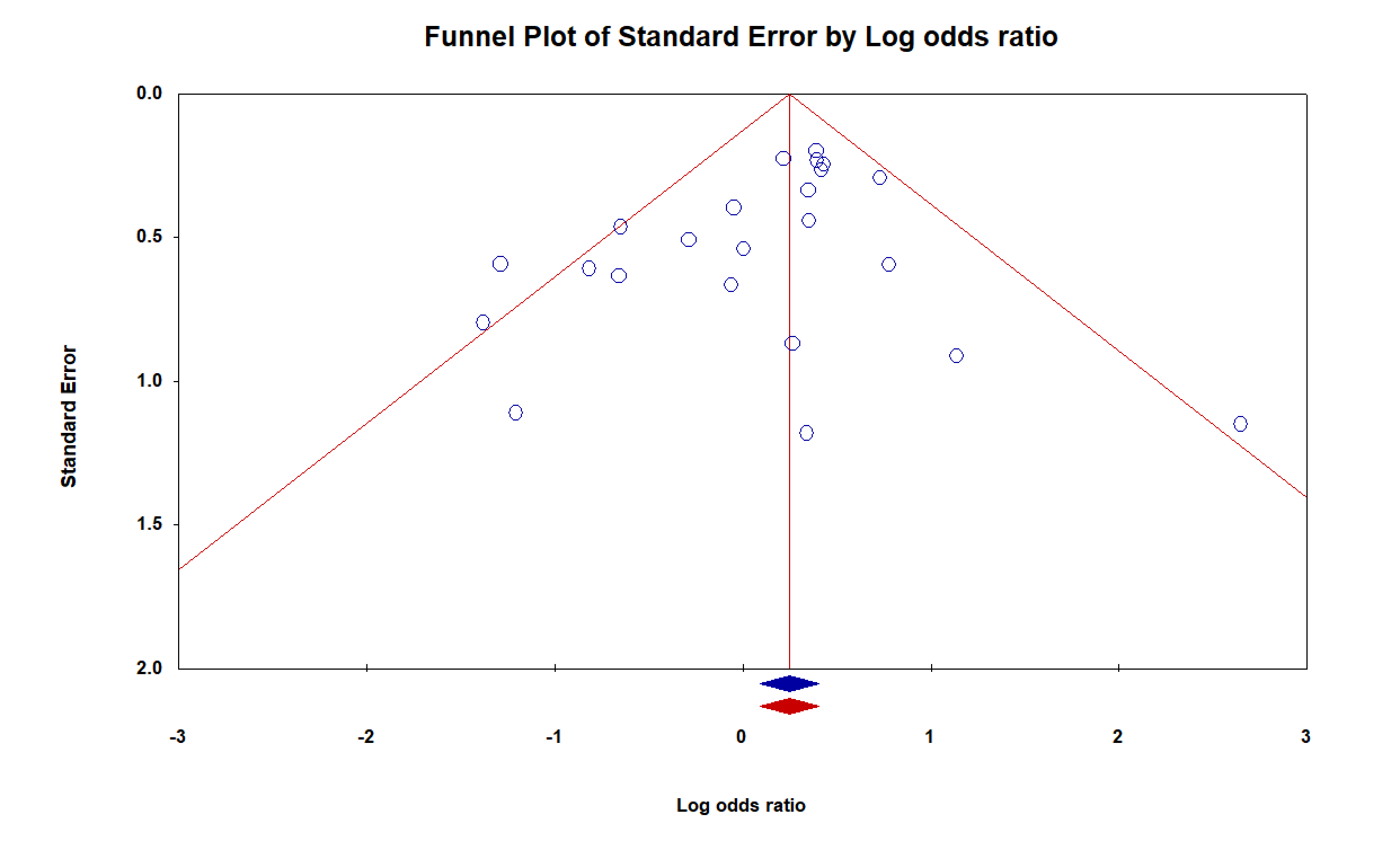


eFigure 7 Funnel plot of standard error against log odds ratio for Cannabis use


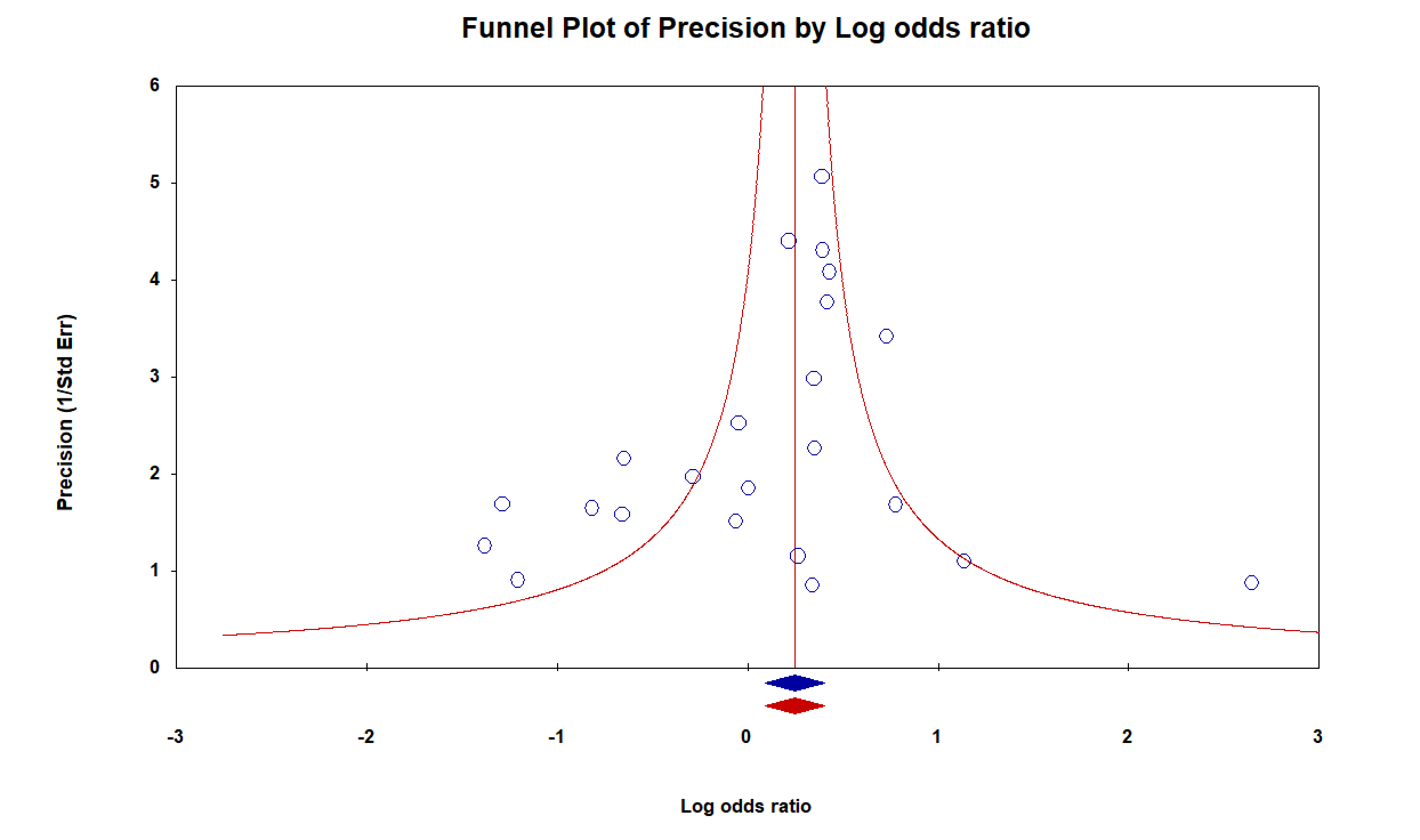


eFigure 8 Funnel plot of precision against log odds ratio for Cannabis use


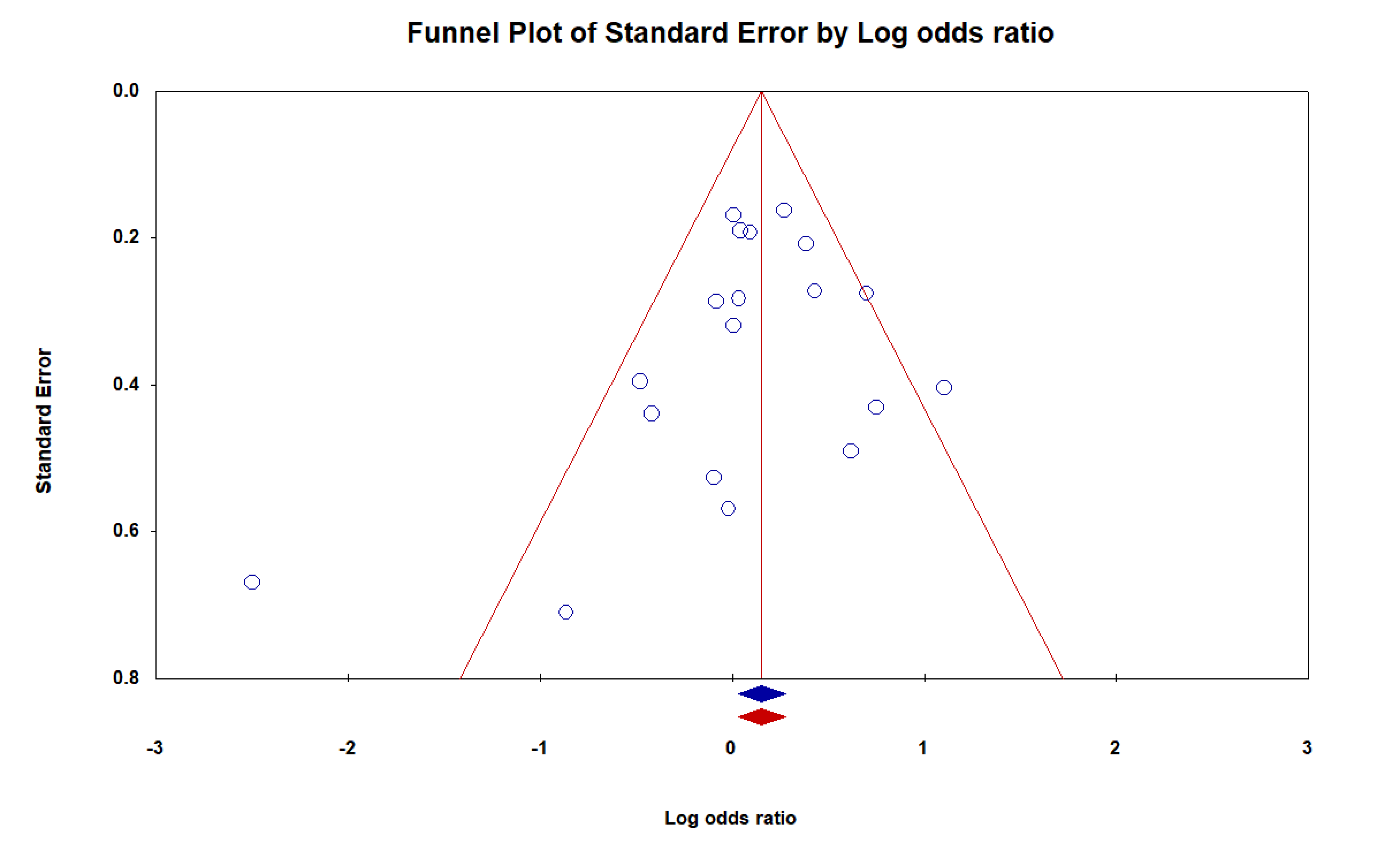


eFigure 9 Funnel plot of standard error against log odds ratio for Co-morbidity


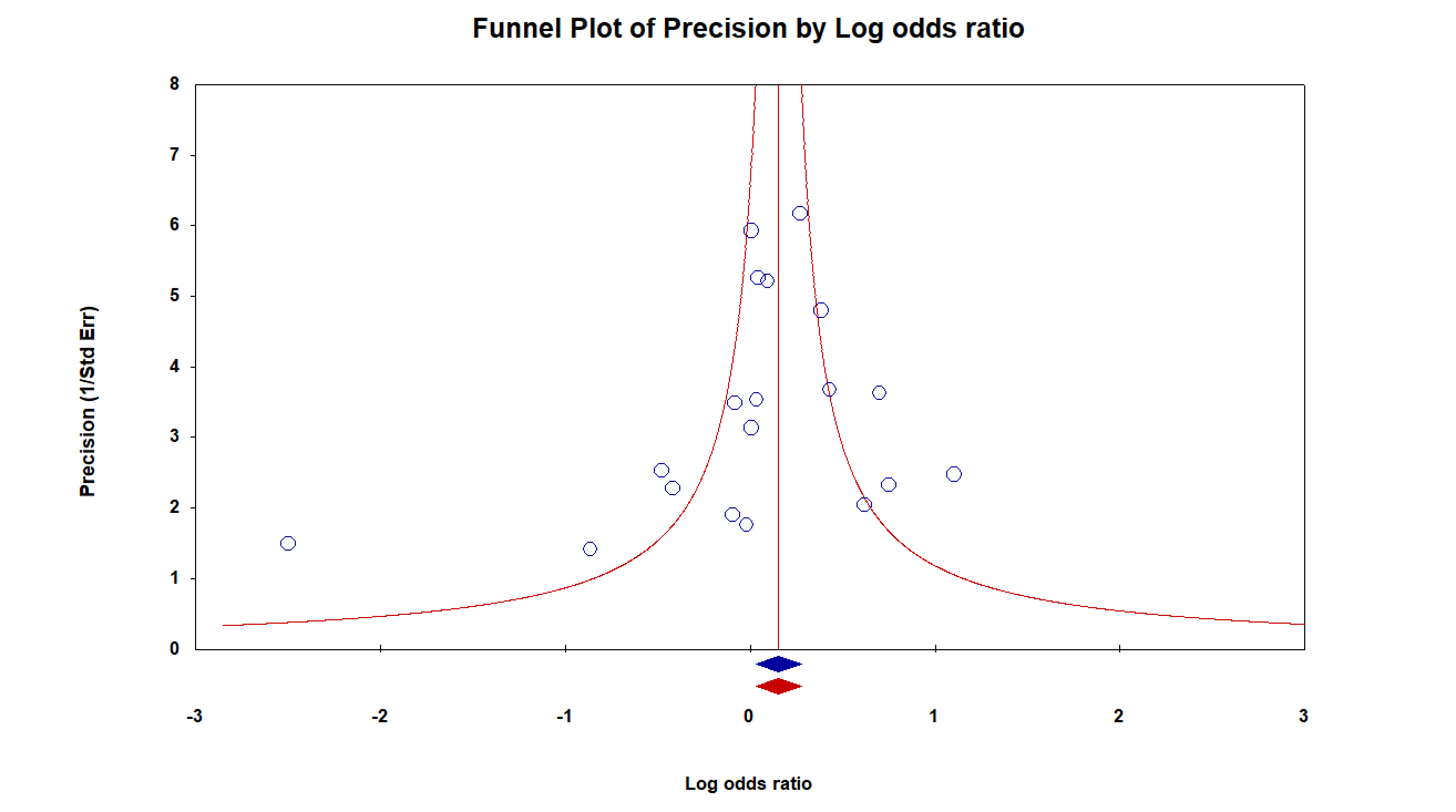


eFigure 10 Funnel plot of precision against log odds ratio for Co-morbidity

eFigure 11 Funnel plot of standard error against standardised difference in means for Disorganised/Cognitive Symptoms

eFigure 12 Funnel plot of precision against standardised difference in means for Disorganised/Cognitive Symptoms

eFigure 13 Funnel plot of standard error against log odds ratio for Education

eFigure 14 Funnel plot of precision against log odds ratio for Education

eFigure 15 Funnel plot of standard error against standardised differences in means for Global functioning

eFigure 16 Funnel plot of precision against standardised differences in means for Global functioning

eFigure 17 Funnel plot of standard error against log odds ratio for Gender

eFigure 18 Funnel plot of precision against log odds ratio for Gender

eFigure 19 Funnel plot of standard error against standardised difference in means for General symptoms

eFigure 20 Funnel plot of precision against standardised difference in means for General symptoms


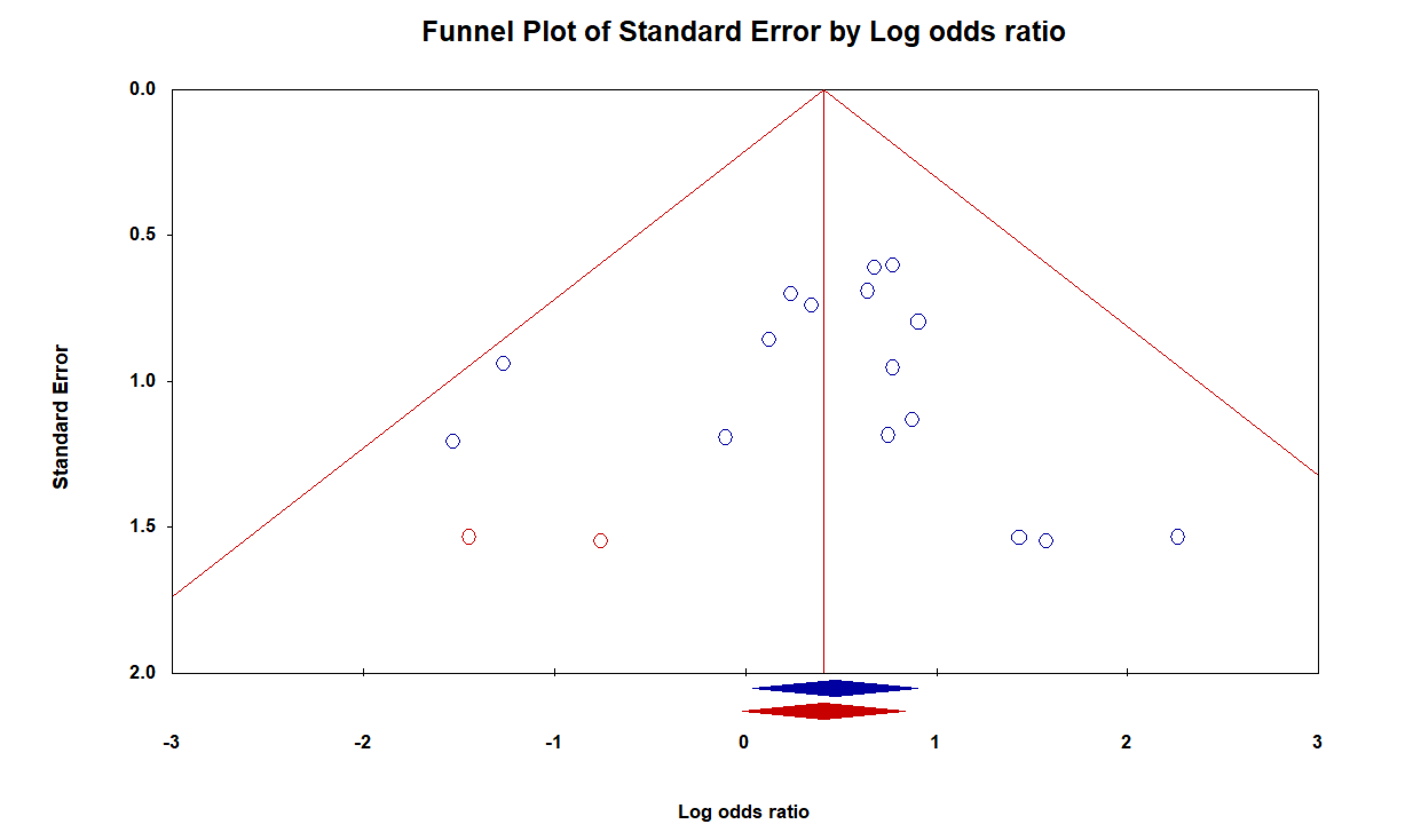


eFigure 21 Funnel plot of standard error against log odds ratio for Handedness

*
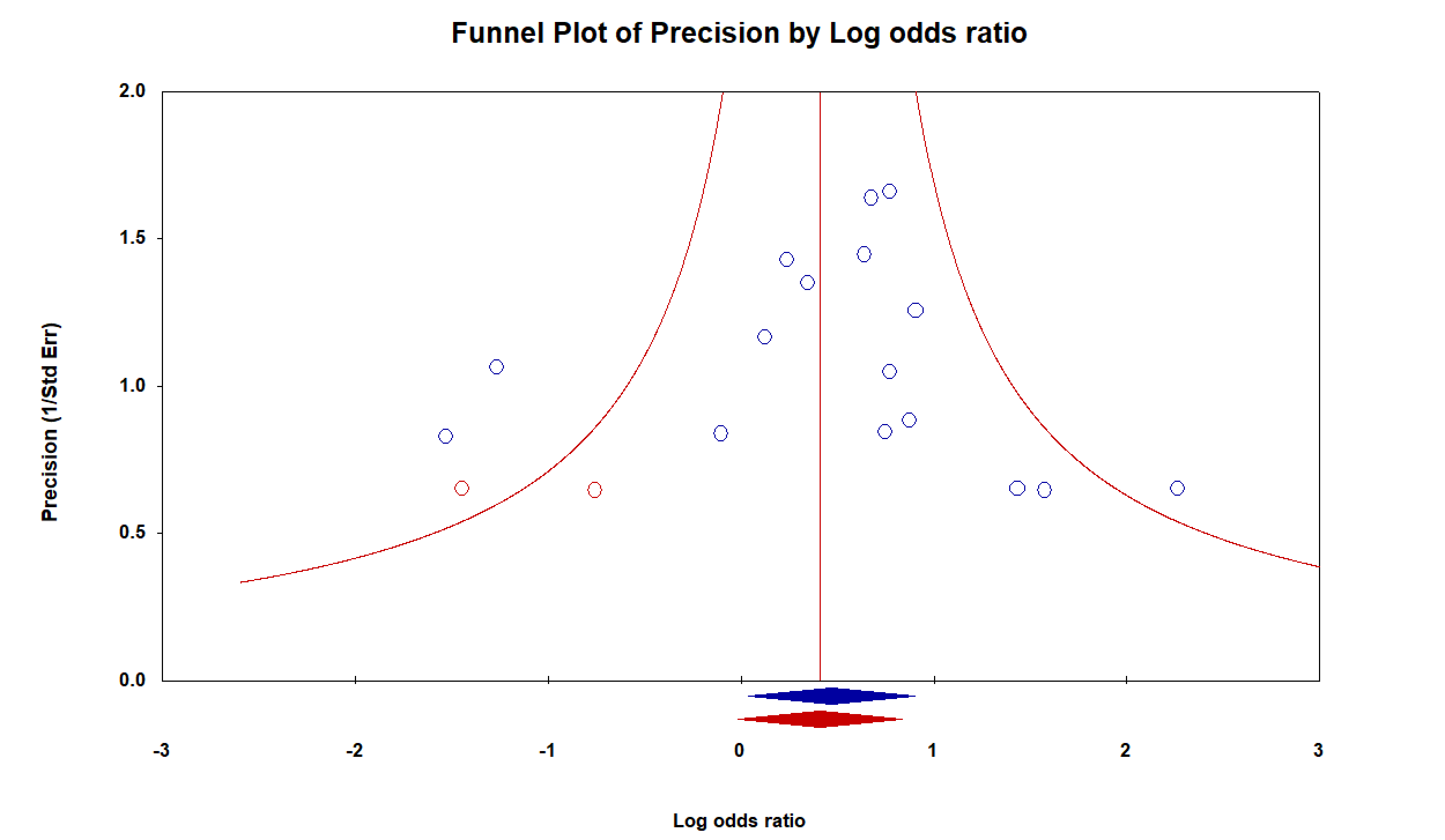
*

eFigure 22 Funnel plot of precision against log odds ratio for Handedness


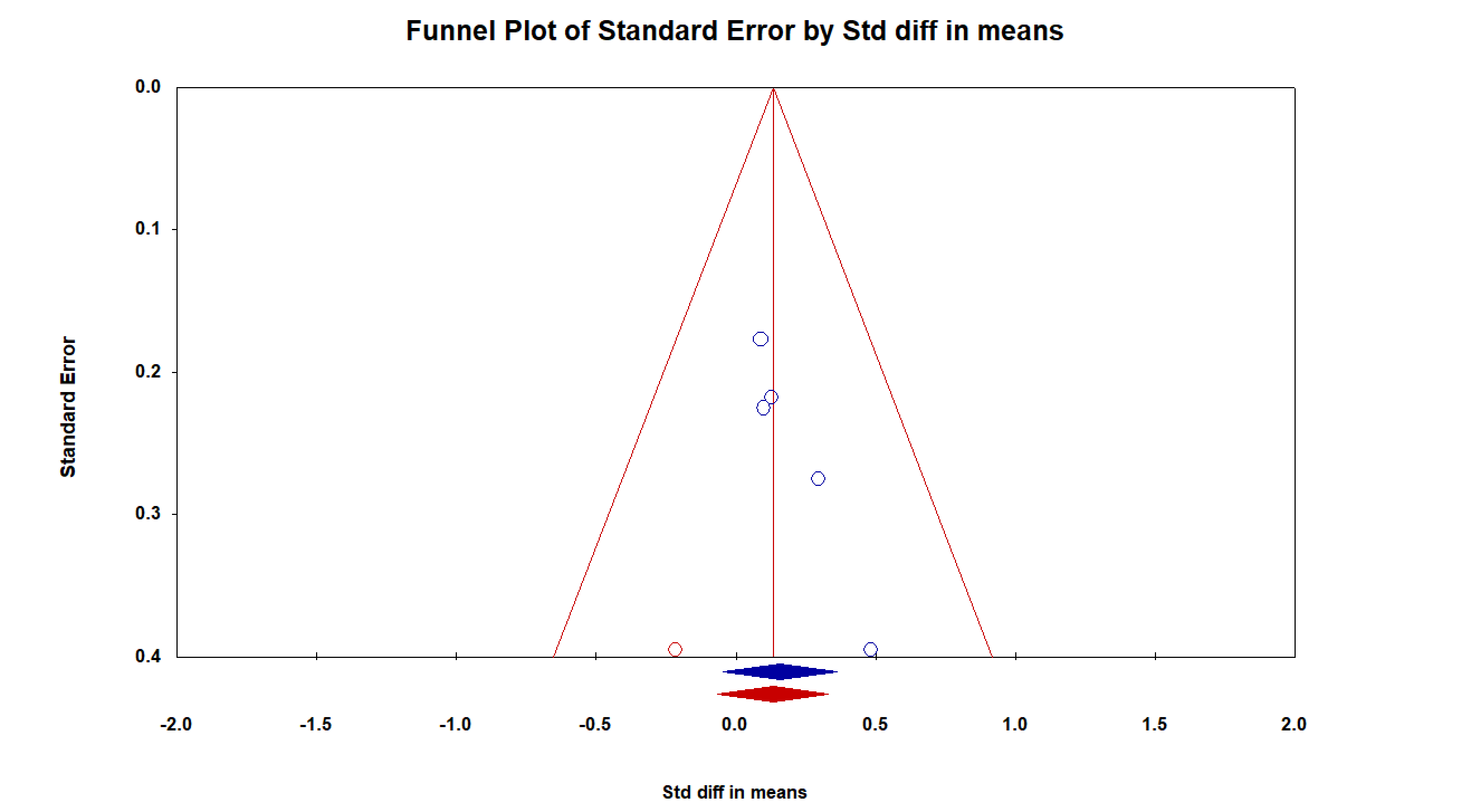


eFigure 23 Funnel plot of standard error against standardised difference in means for Height

*
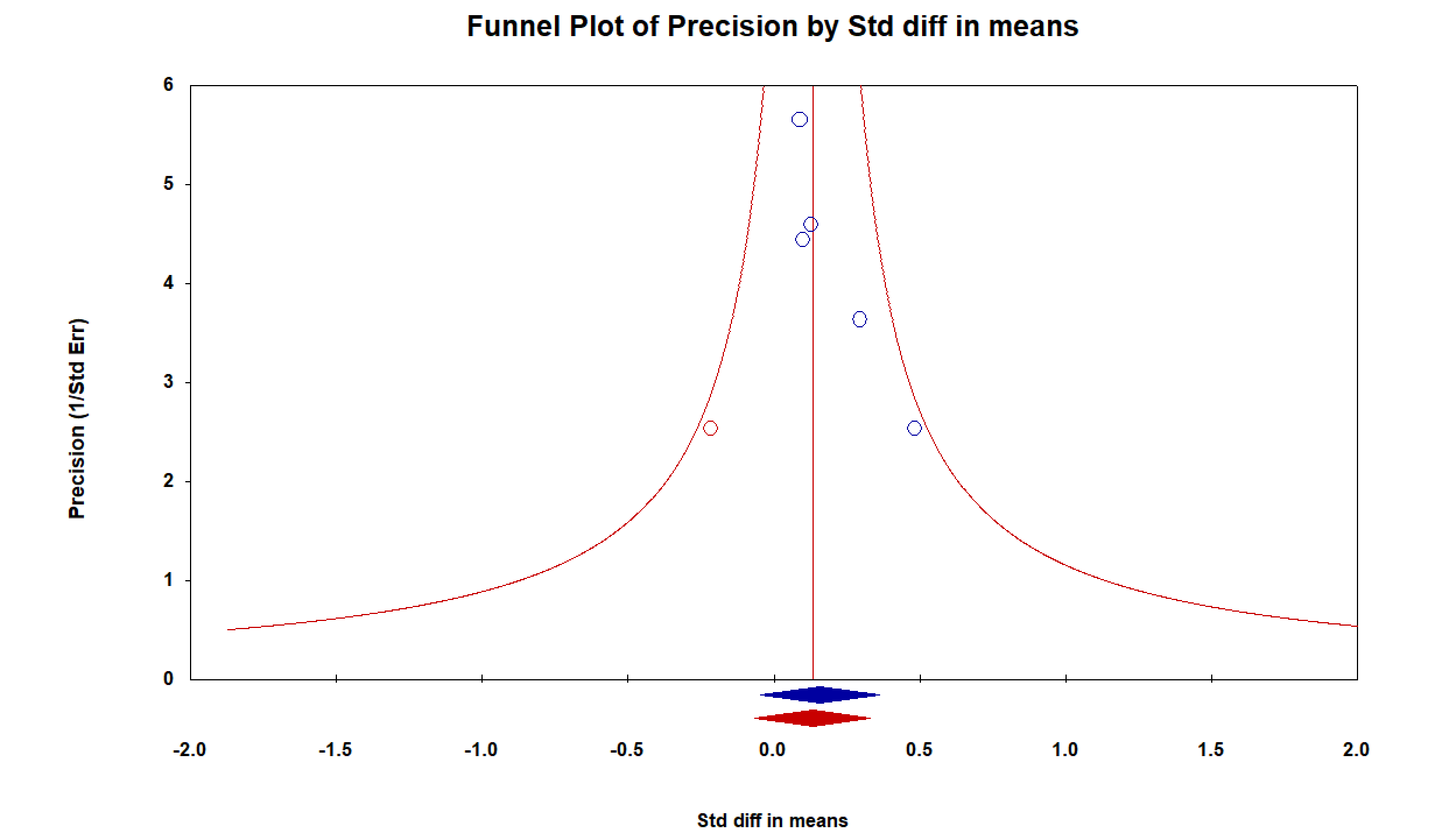
*

eFigure 24 Funnel plot of precision against standardised difference in means for Height

*
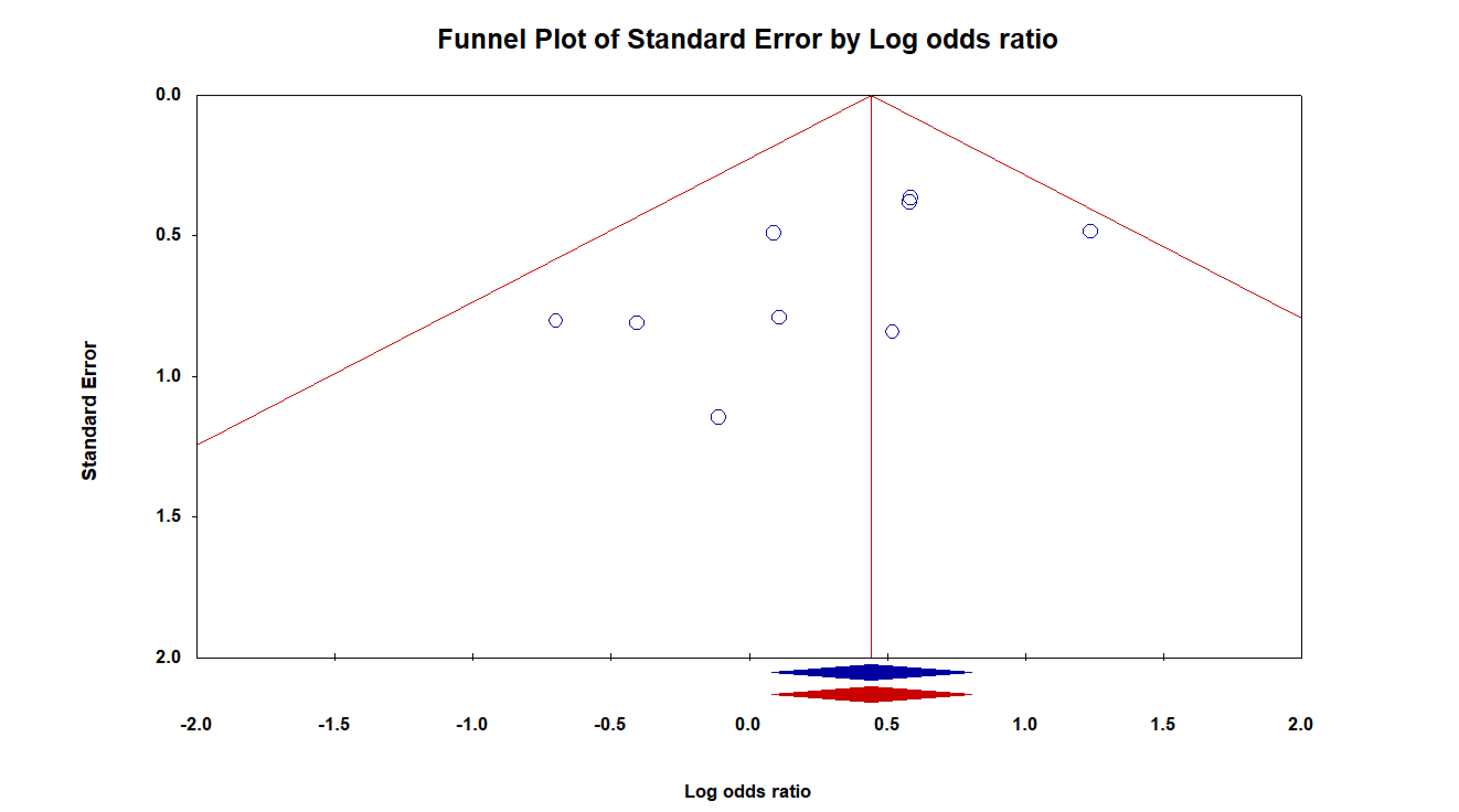
*

eFigure 25 Funnel plot of standard error against log odds ratio for Living status


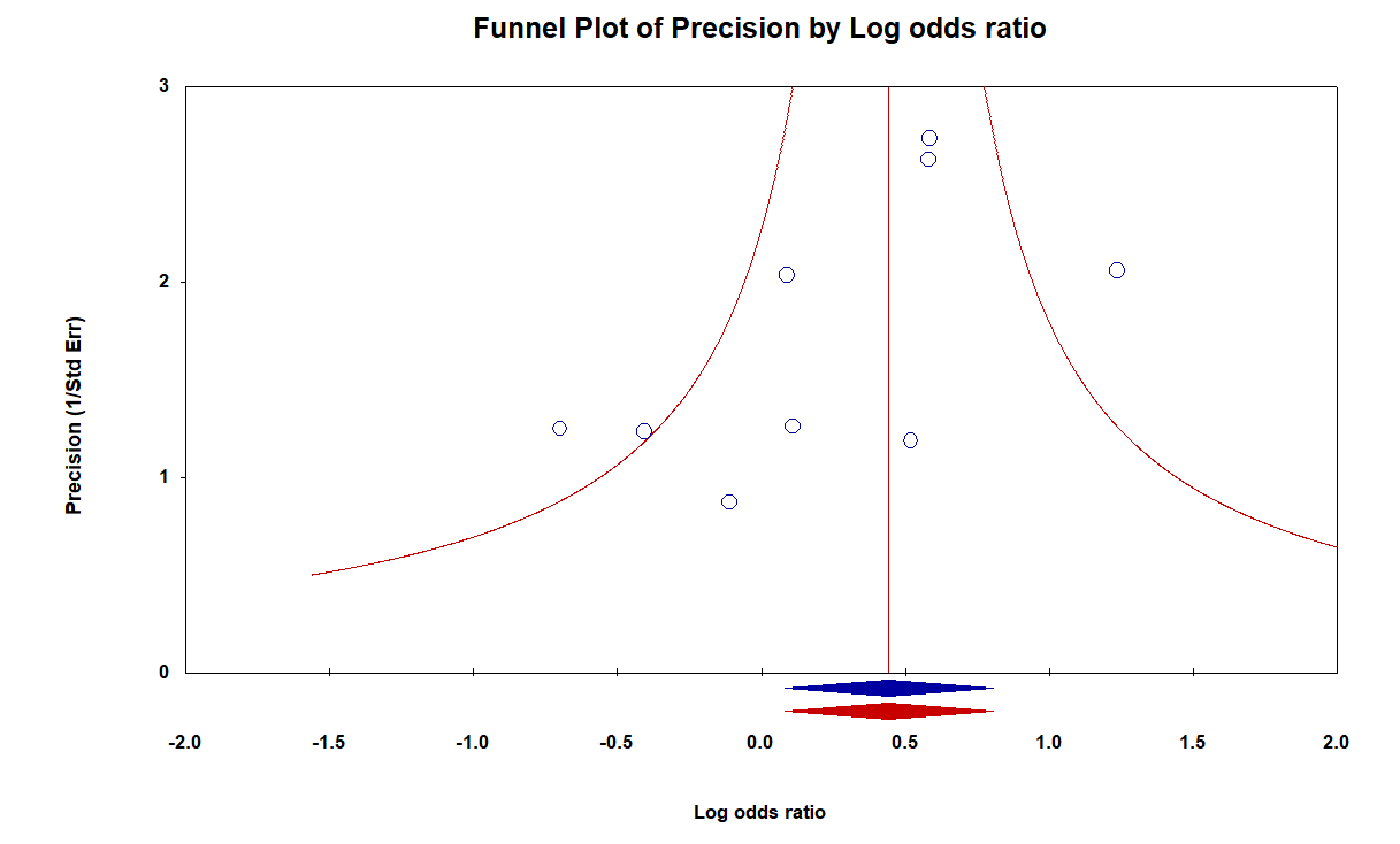


eFigure 26 Funnel plot of precision against log odds ratio for Living status

**

eFigure 27 Funnel plot of standard error against standardised mean difference for Negative psychotic symptoms

**

eFigure 28 Funnel plot of precision against standardised mean difference for Negative psychotic symptoms

**

eFigure 29 Funnel plot of standard error against log odds ratio for Non-white ethnicity

**

eFigure 30 Funnel plot of precision against log odds ratio for Non-white ethnicity

*
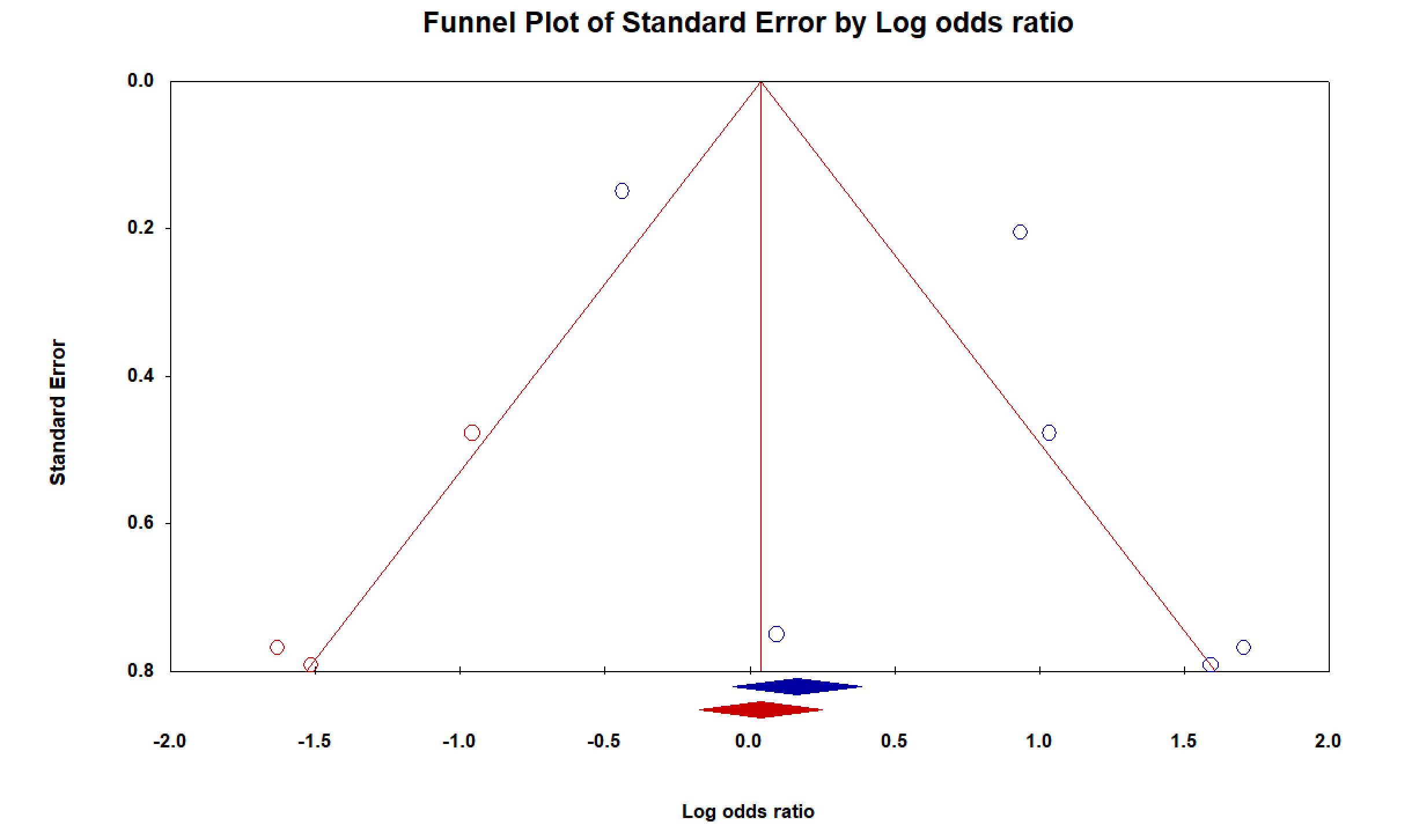
*

eFigure 31 Funnel plot of standard error against log odds ratio for Perinatal complications

*
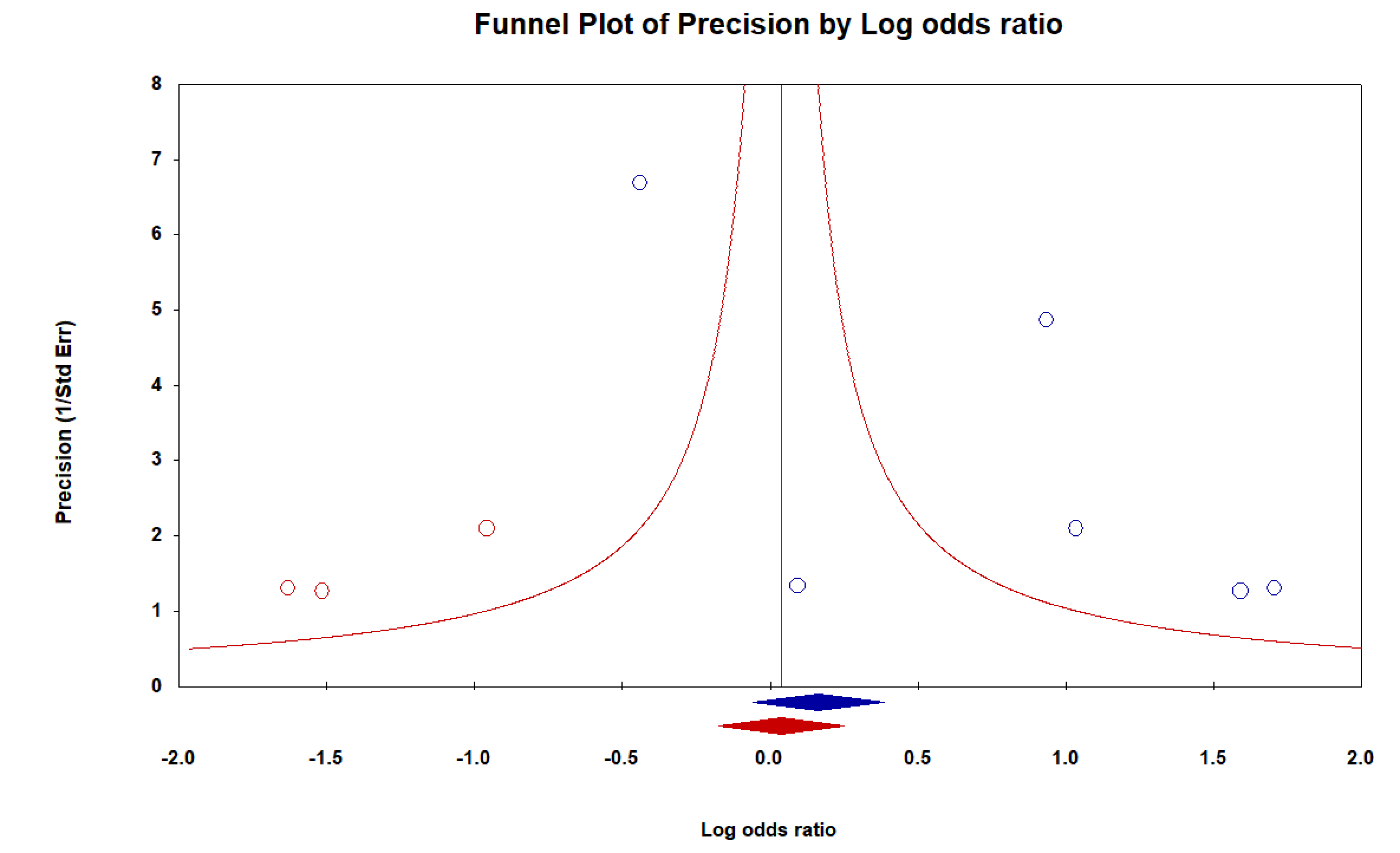
*

eFigure 32 Funnel plot of precision against log odds ratio for Perinatal complications

**

eFigure 33 Funnel plot of standard error against standardised mean difference for Attenuated positive psychotic symptoms

**

eFigure 34 Funnel plot of precision against standardised mean difference for Attenuated positive psychotic symptoms


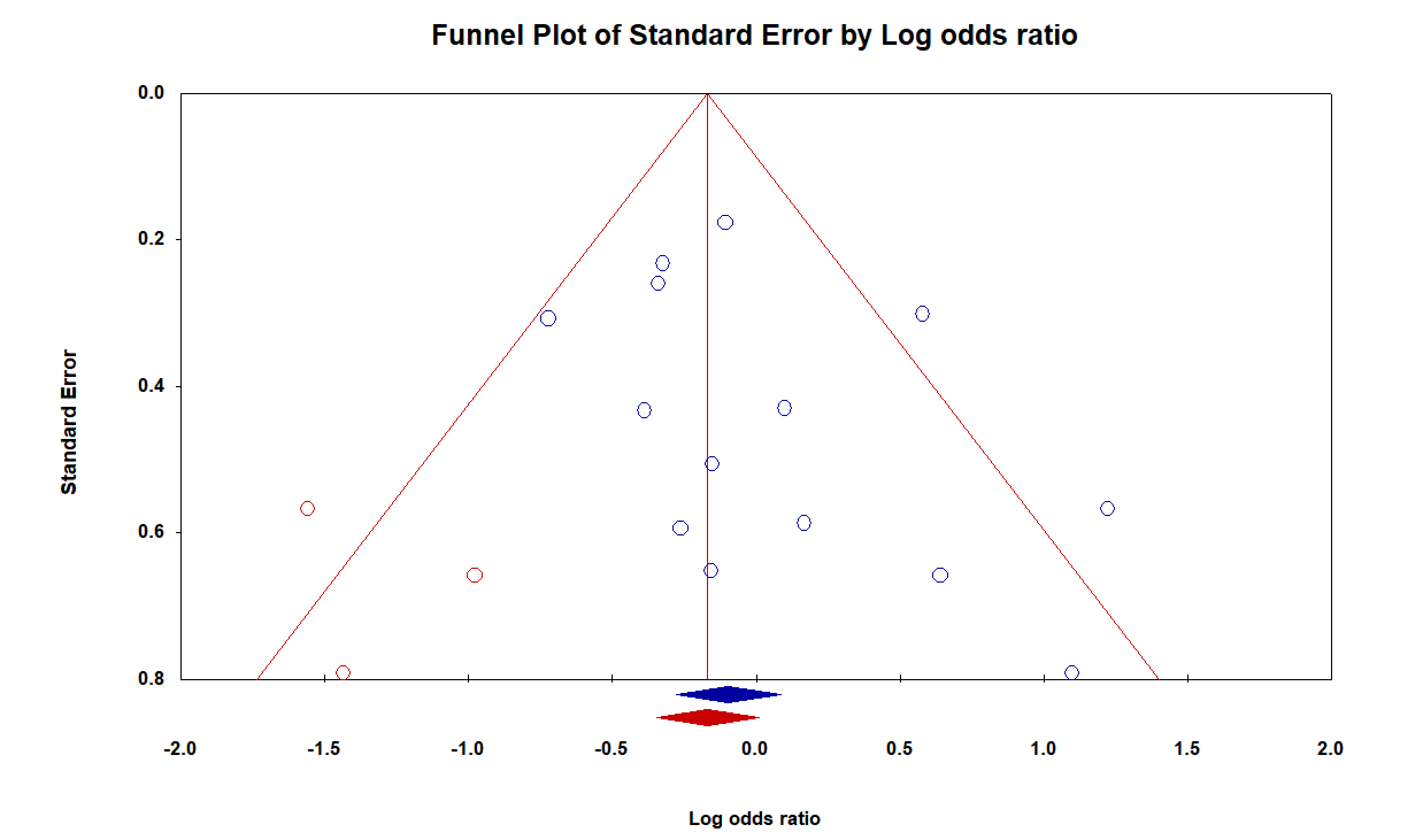


eFigure 35 Funnel plot of standard error against log odds ratio for Socioeconomic status

*
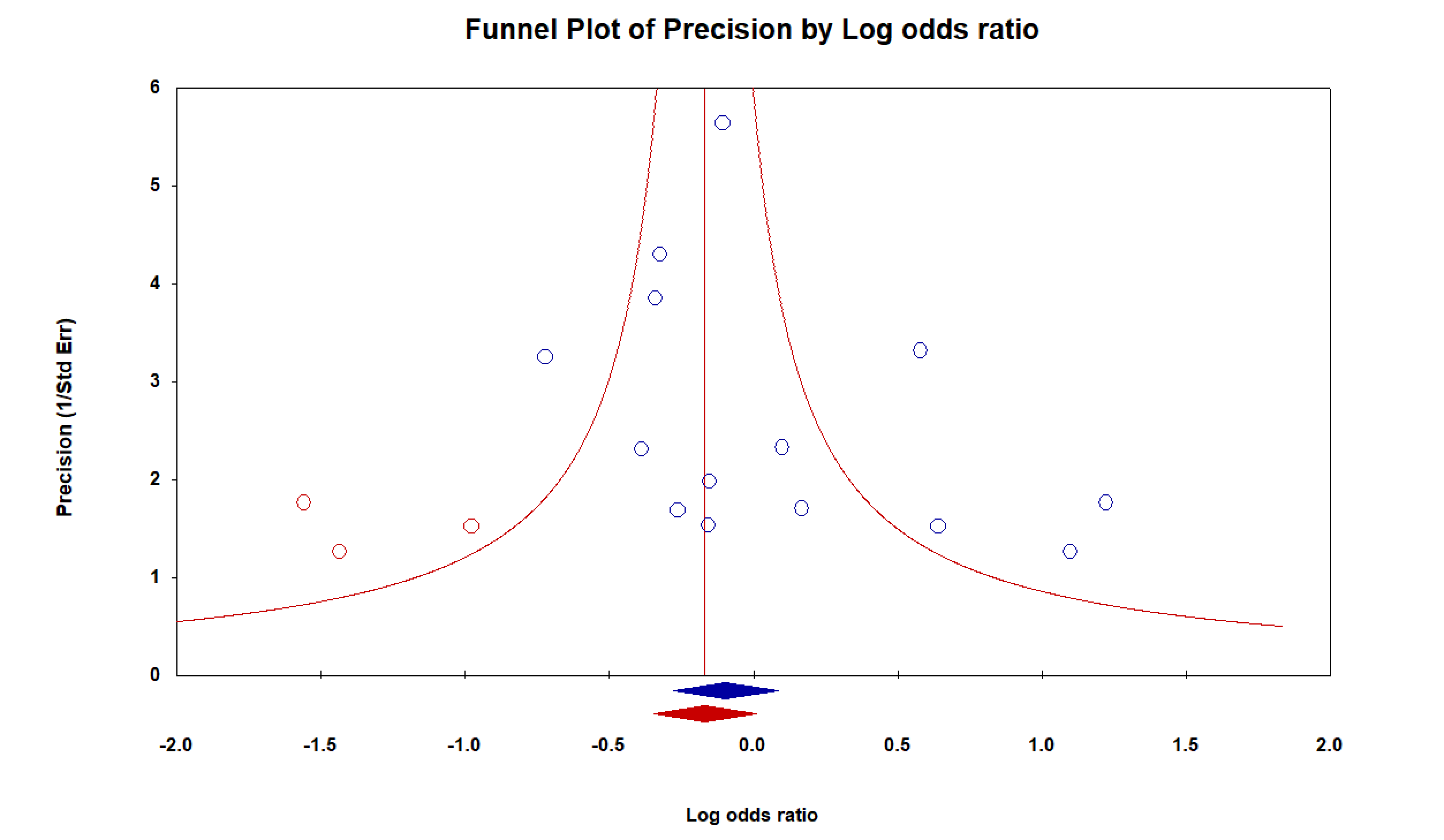
*

eFigure 36 Funnel plot of precision against log odds ratio for Socioeconomic status

*
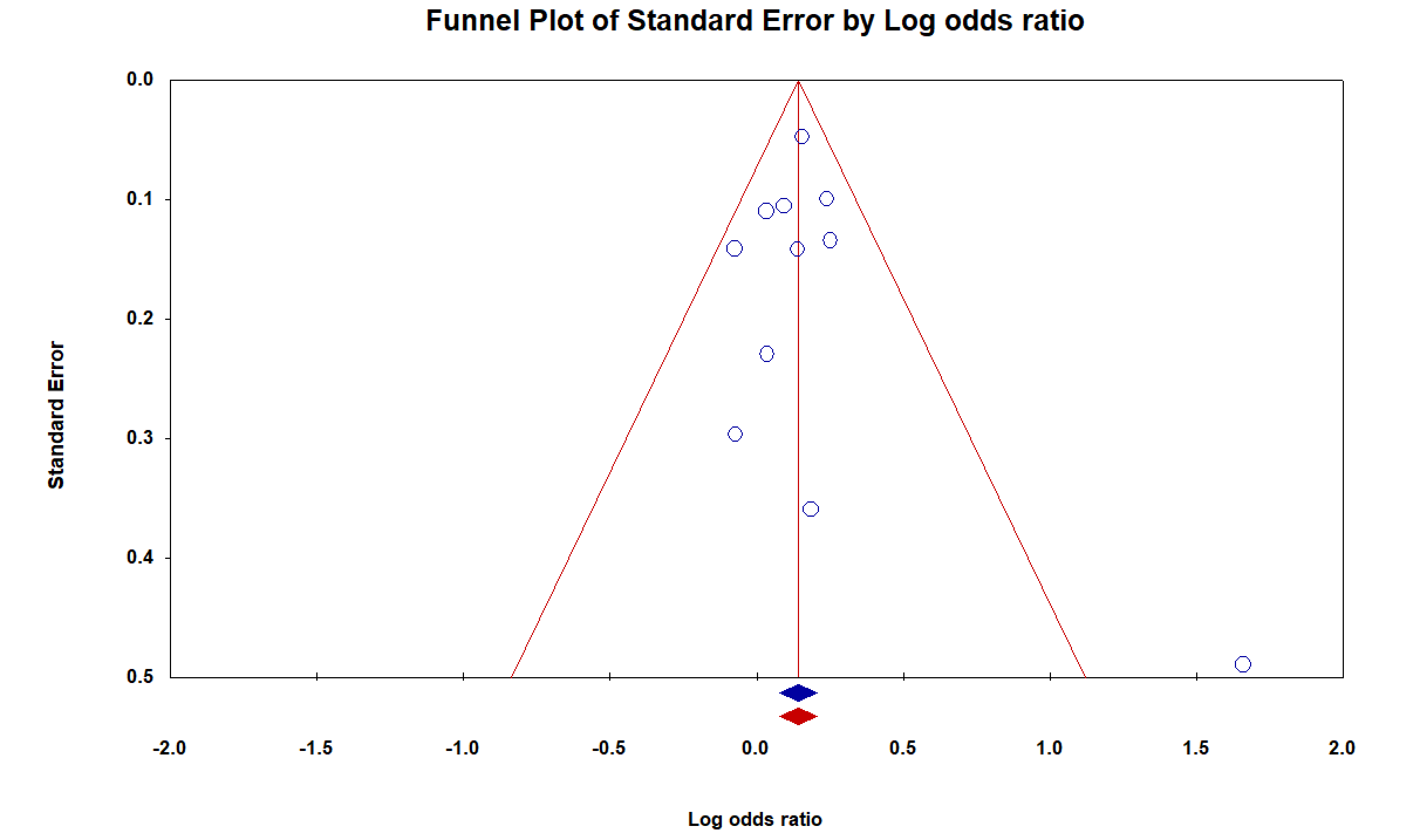
*

eFigure 37 Funnel plot of standard error against log odds ratio for Stress & trauma


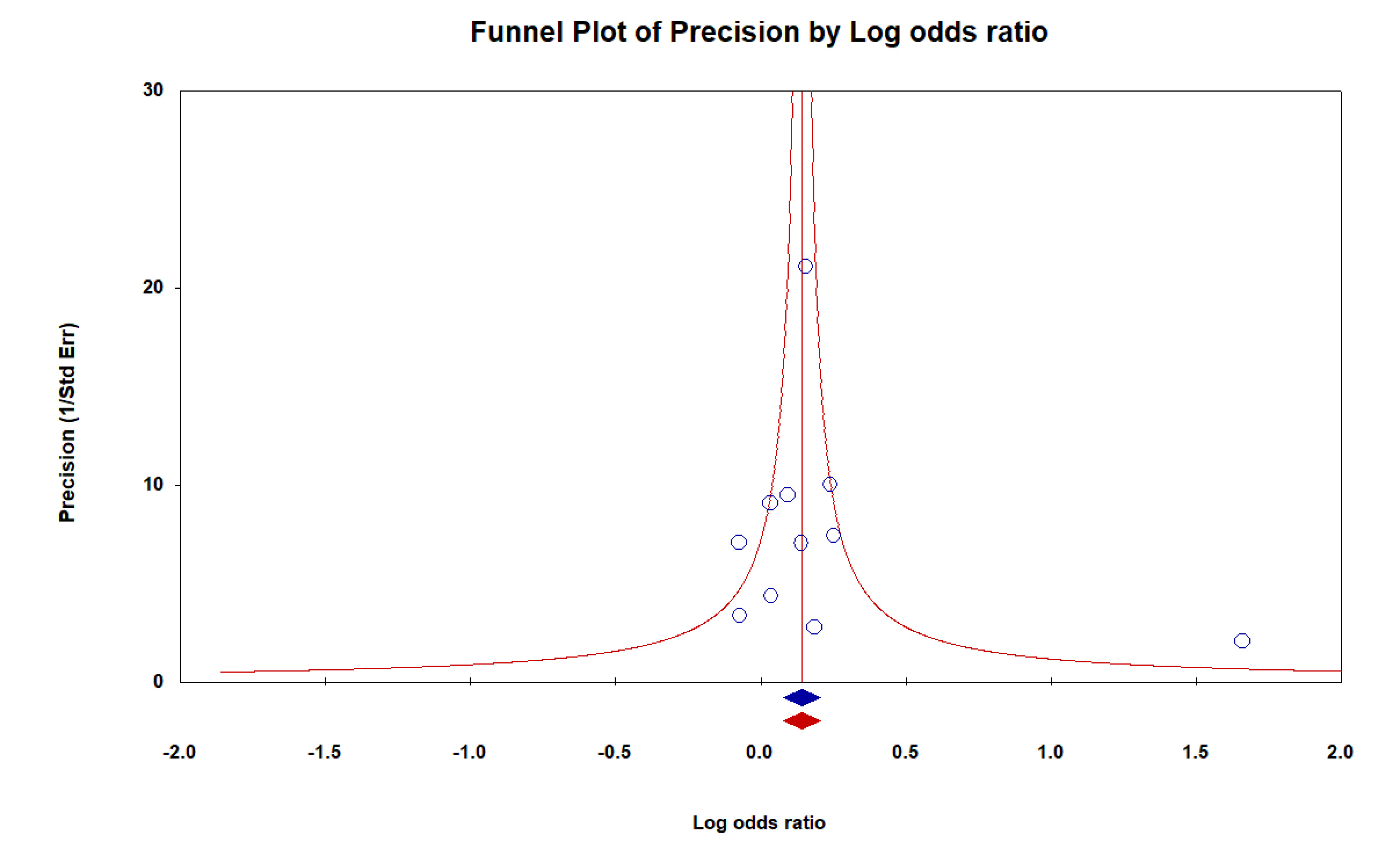


eFigure 38 Funnel plot of precision against log odds ratio for Stress & trauma


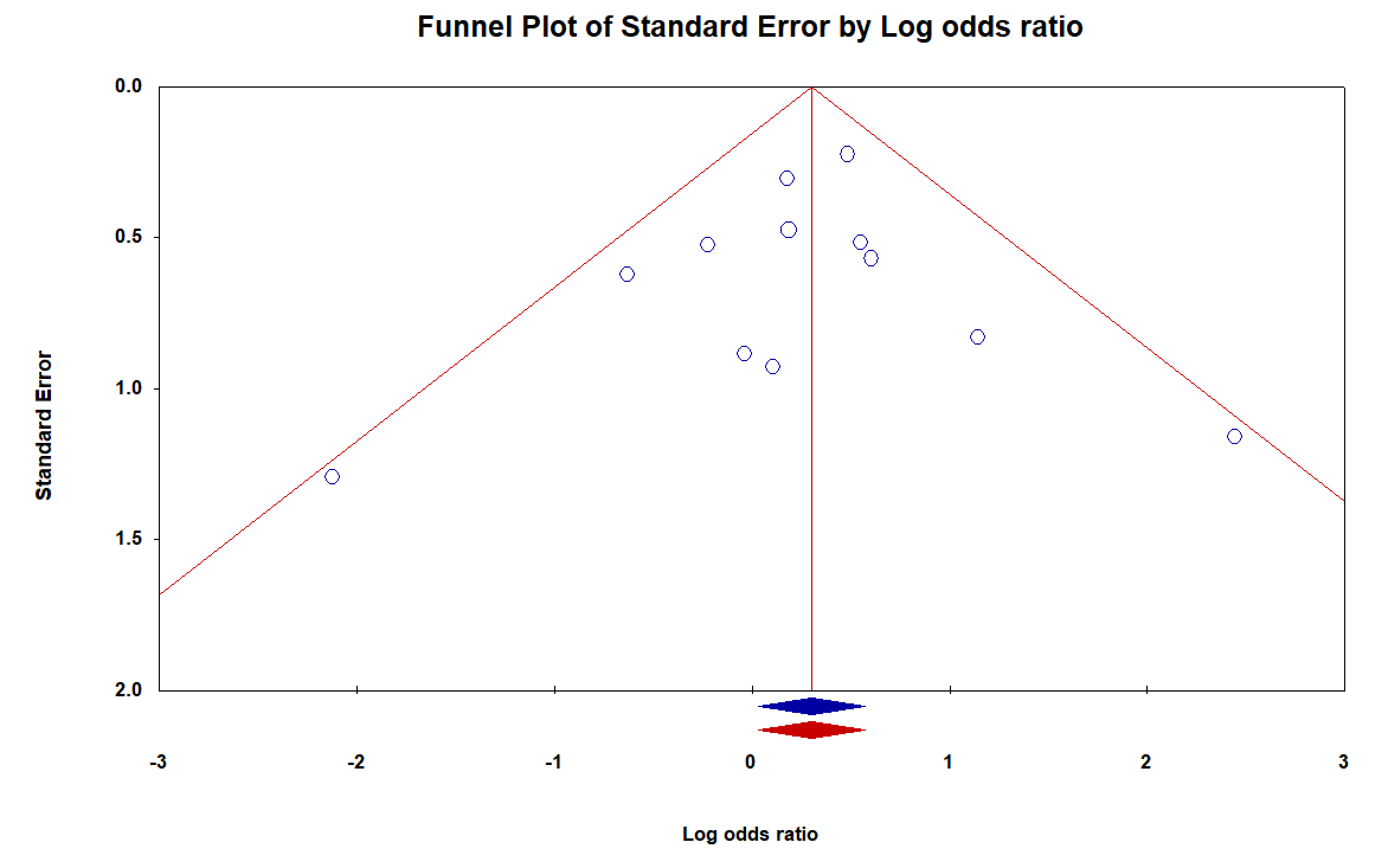


eFigure 39 Funnel plot of standard error against log odds ratio for Substance misuse


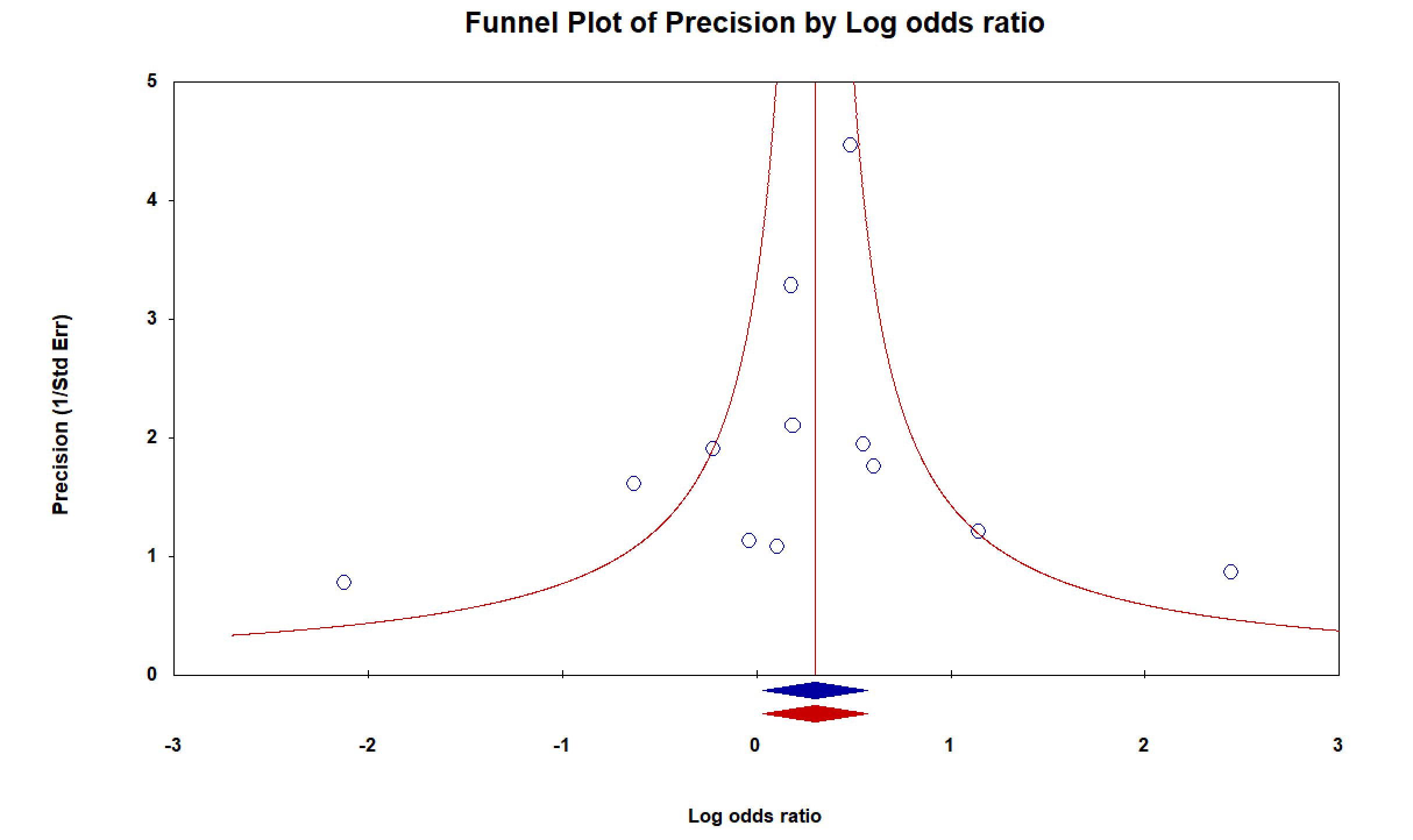


eFigure 40 Funnel plot of precision against log odds ratio for Substance misuse


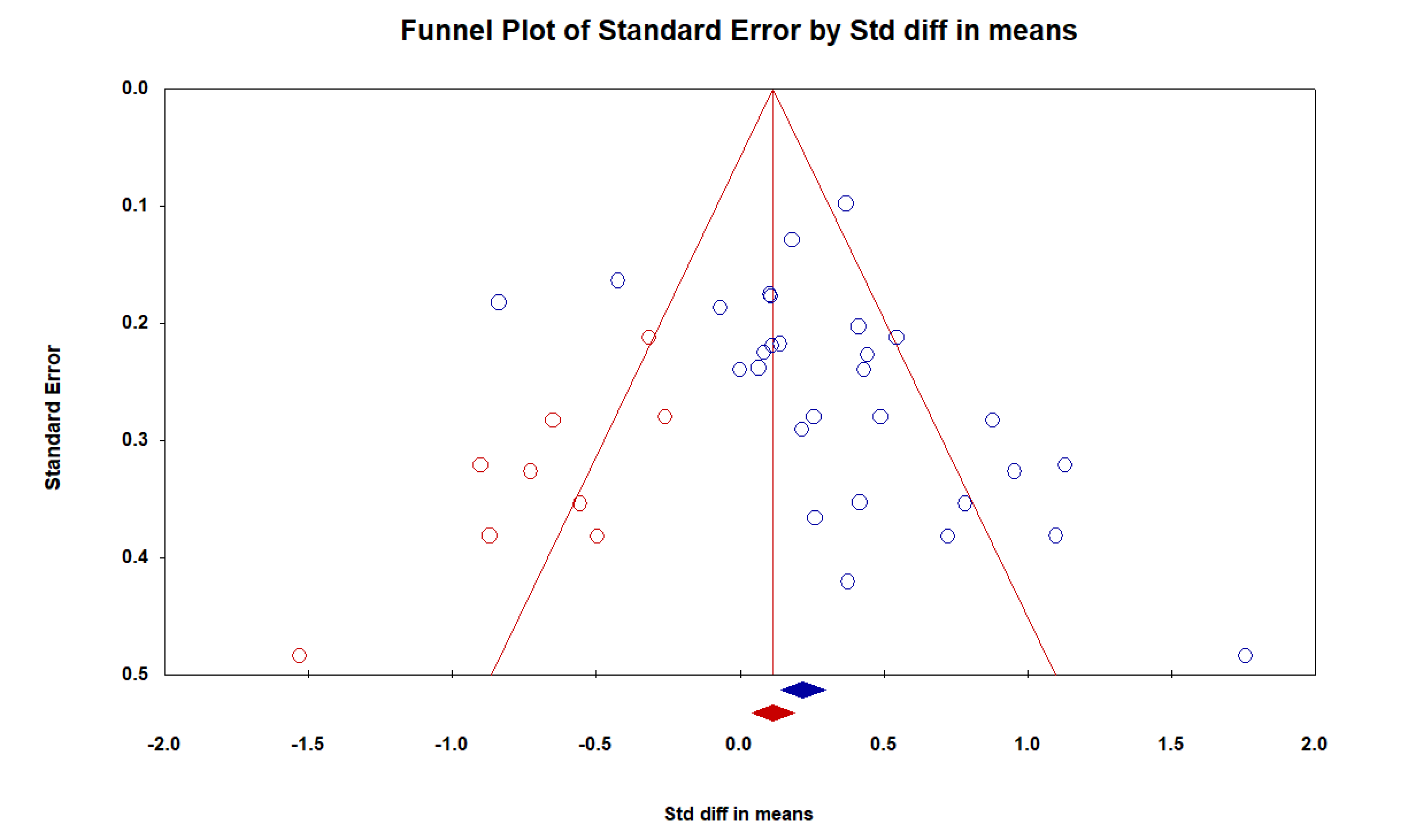


eFigure 41 Funnel plot of standard error against standardised mean difference for Total attenuated psychotic symptoms score


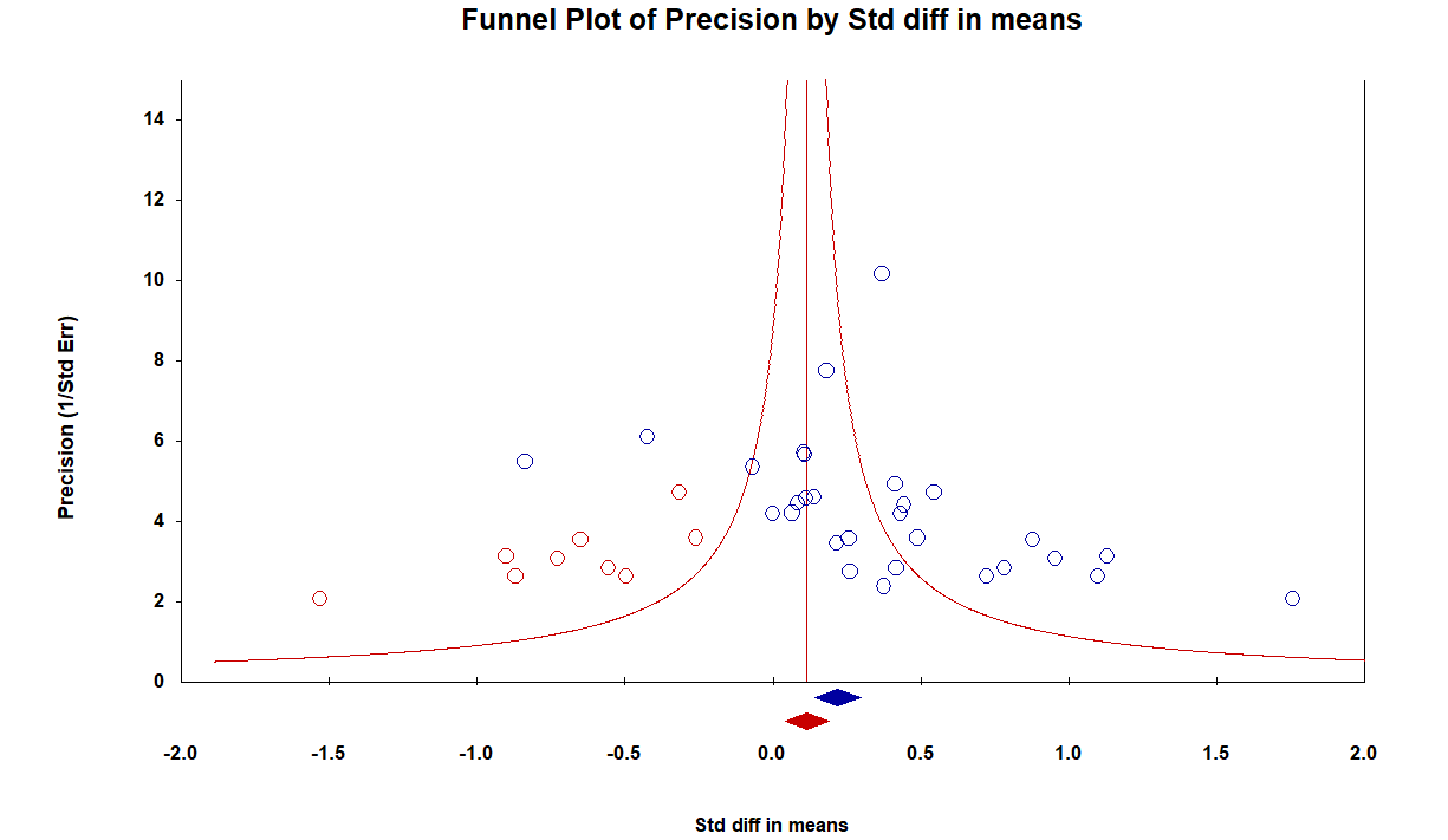


eFigure 42 Funnel plot of precision against standardised mean difference for Total attenuated psychotic symptoms score


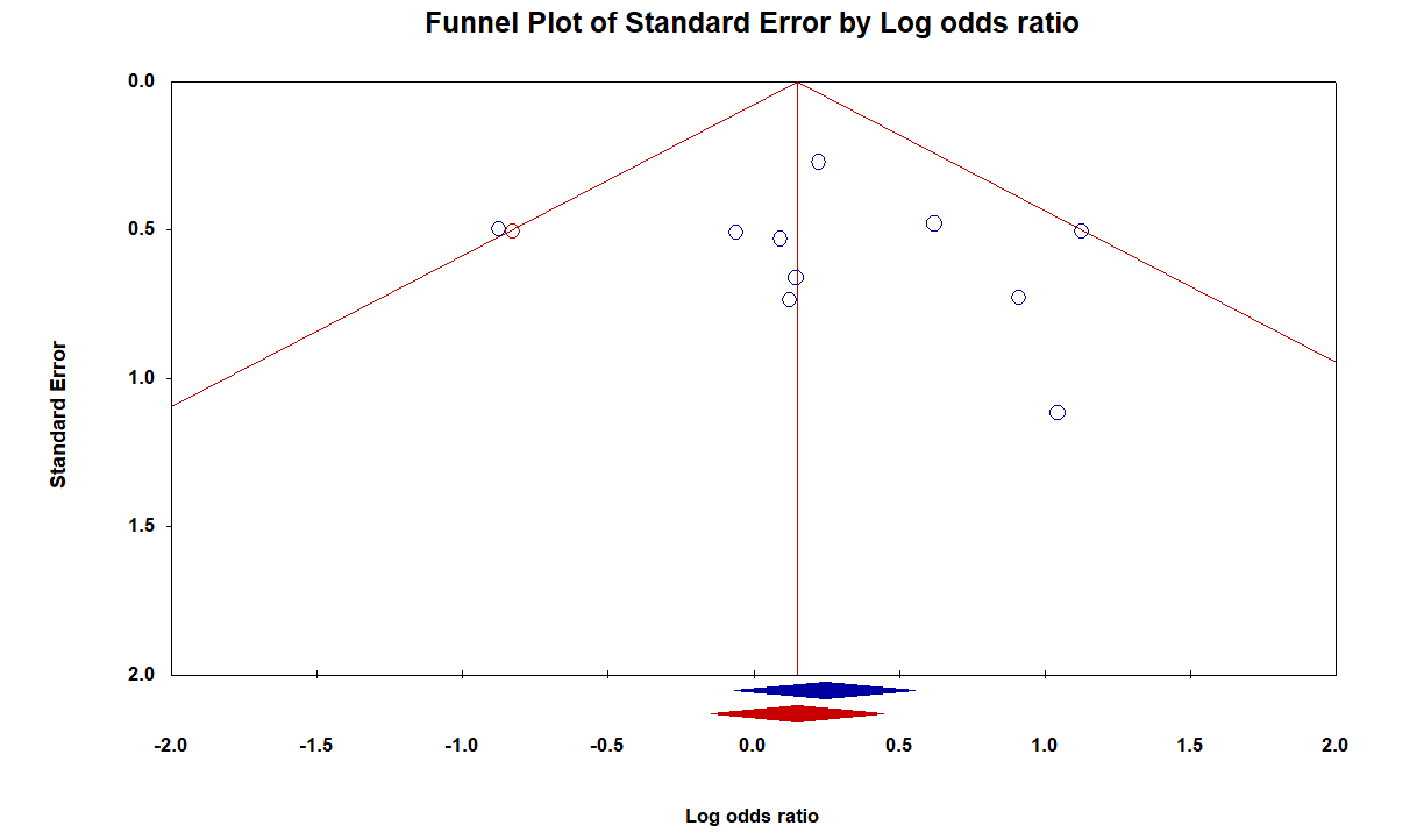


eFigure 43 Funnel plot of standard error log odds ratio for Tobacco use


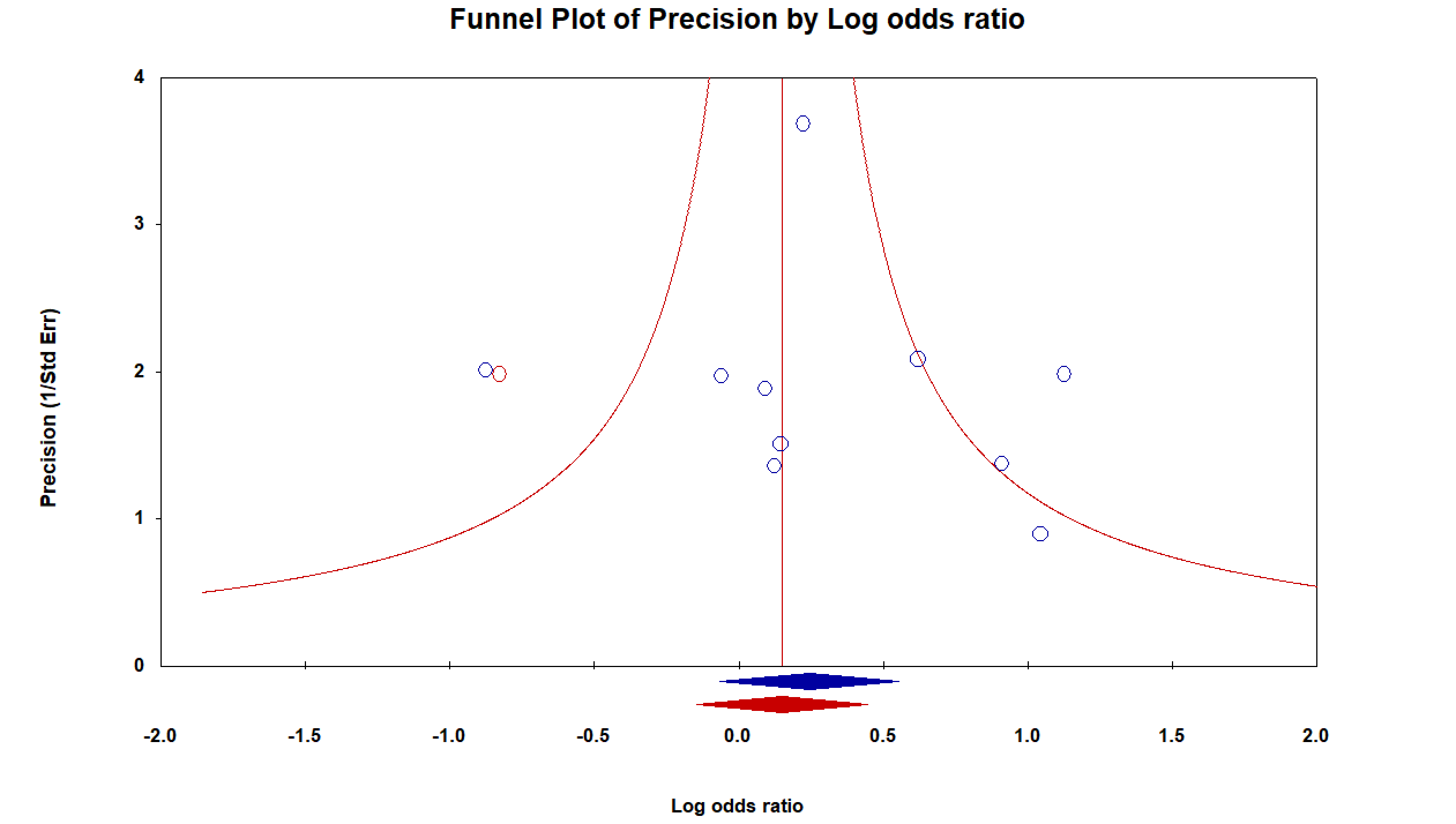


eFigure 44 Funnel plot of precision against standardised mean difference for Tobacco use

eFigure 45 Funnel plot of standard error against log odds ratio for Employment

eFigure 46 Funnel plot of precision against log odds ratio for Employment


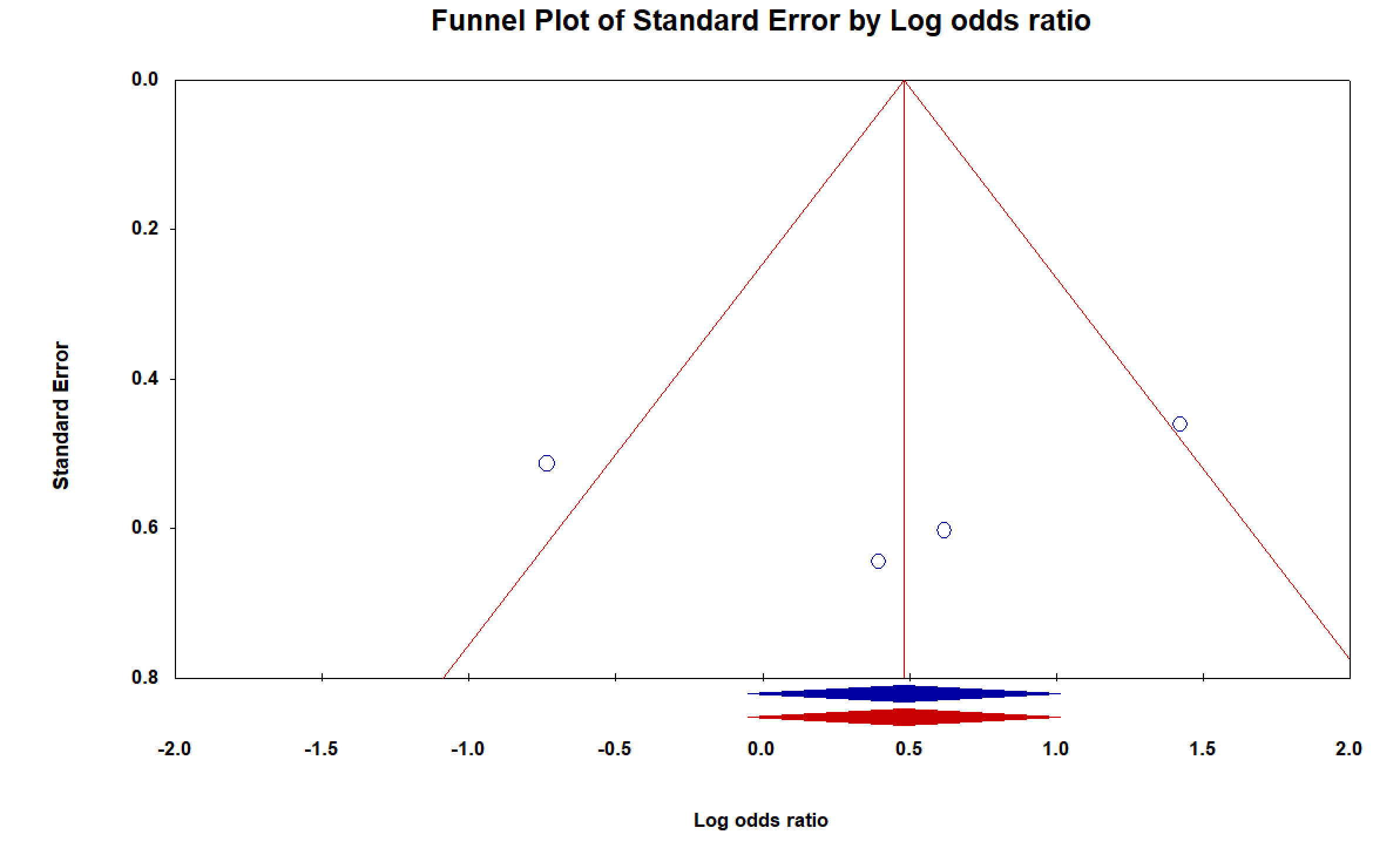


eFigure 47 Funnel plot of standard error against log odds ratio for Urbanicity


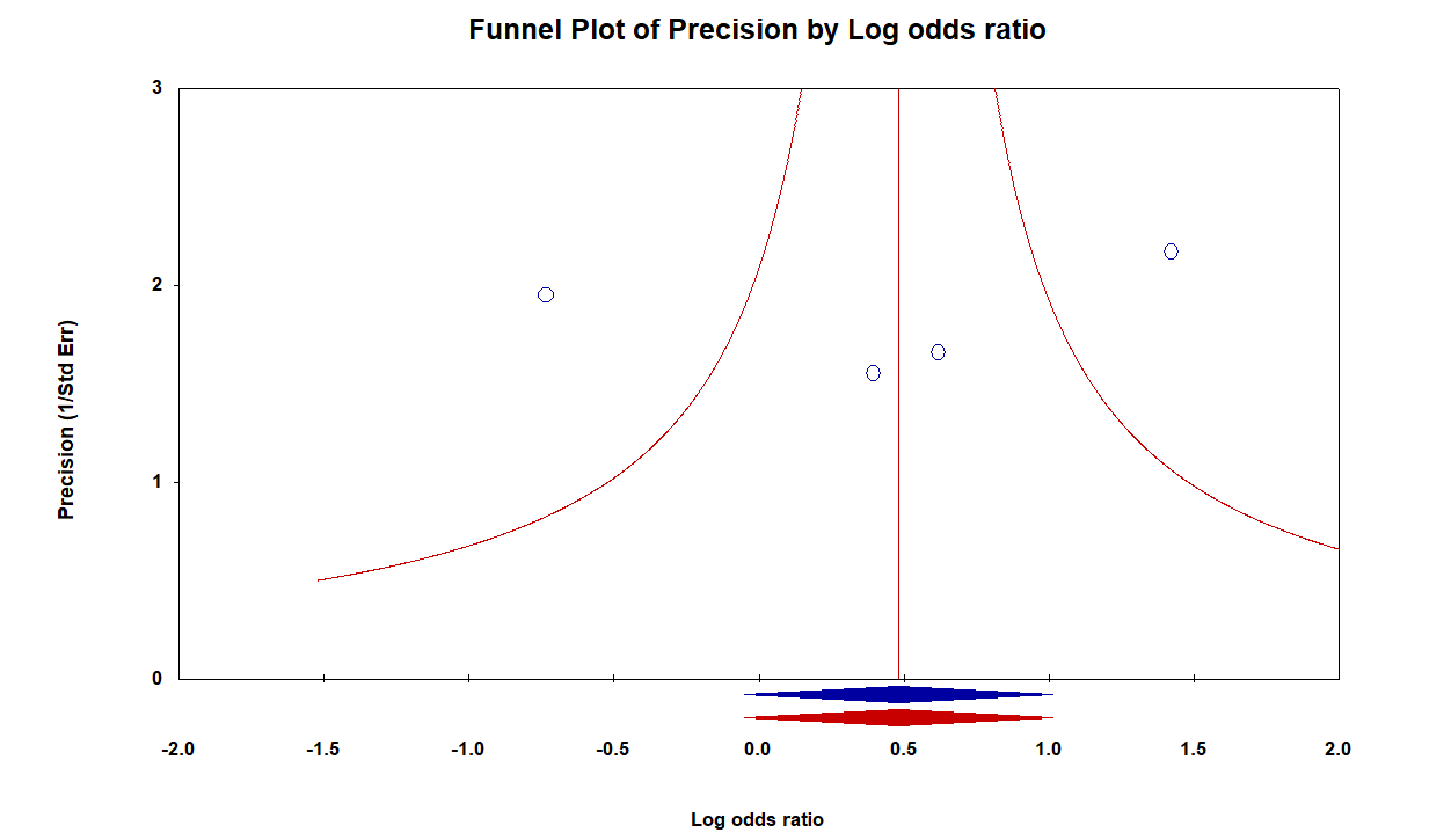


eFigure 48 Funnel plot of precision against log odds ratio for Urbanicity

**eReferences**

1. Addington J, Liu L, Perkins DO, Carrion RE, Keefe RSE, Woods SW. The role of cognition and social functioning as predictors in the transition to psychosis for youth with attenuated psychotic symptoms. *Schizophr. Bull.* 2017;43(1):57-63. doi:10.1093/schbul/sbw152.

2. Addington J, Piskulic D, Liu L, et al. Comorbid diagnoses for youth at clinical high risk of psychosis. *Schizophr. Res.* 2017;190:90-95. doi:10.1016/j.schres.2017.03.043.

3. Alderman T, Addington J, Bearden C, et al. Negative symptoms and impaired social functioning predict later psychosis in Latino youth at clinical high risk in the North American prodromal longitudinal studies consortium. *Early Interv. Psychiatry* 2015;9(6):467-475. doi:10.1111/eip.12128.

4. Allott KA, Schäfer MR, Thompson A, et al. Emotion recognition as a predictor of transition to a psychotic disorder in ultra-high risk participants. *Schizophr. Res.* 2014;153(1-3):25-31. doi:10.1016/j.schres.2014.01.037.

5. Armando M, Pontillo M, De Crescenzo F, et al. Twelve-month psychosis-predictive value of the ultra-high risk criteria in children and adolescents. *Schizophr. Res.* 2015;169(1-3):186-192. doi:10.1016/j.schres.2015.10.033.

6. Atkinson RJ, Fulham WR, Michie PT, et al. Electrophysiological, cognitive and clinical profiles of at-risk mental state: The longitudinal Minds in Transition (MinT) study. *PLoS One* 2017;12(2):e0171657. doi:10.1371/journal.pone.0171657.

7. Auther AM, McLaughlin D, Carrión RE, Nagachandran P, Correll CU, Cornblatt BA. Prospective study of cannabis use in adolescents at clinical high risk for psychosis: impact on conversion to psychosis and functional outcome. *Psychol. Med.* 2012;42(12):2485-2497. doi:10.1017/S0033291712000803.

8. Auther AM, Cadenhead KS, Carrión RE, et al. Alcohol confounds relationship between cannabis misuse and psychosis conversion in a high-risk sample. *Acta Psychiatr. Scand.* 2015;132(1):60-68. doi:10.1111/acps.12382.

9. Bang M, Park JY, Kim KR, et al. Psychotic conversion of individuals at ultra-high risk for psychosis: The potential roles of schizotypy and basic symptoms. *Early Interv. Psychiatry* 2017. doi:10.1111/eip.12518.

10. Bearden CE, Wu KN, Caplan R, Cannon TD. Thought disorder and communication deviance as predictors of outcome in youth at clinical high risk for psychosis. *J. Am. Acad. Child Adolesc. Psychiatry* 2011;50(7):669-680. doi:10.1016/j.jaac.2011.03.021.

11. Bechdolf A, Thompson A, Nelson B, et al. Experience of trauma and conversion to psychosis in an ultra-high-risk (prodromal) group. *Acta Psychiatr. Scand.* 2010;121(5):377-384. doi:10.1111/j.1600-0447.2010.01542.x.

12. Bedi G, Carrillo F, Cecchi GA, et al. Automated analysis of free speech predicts psychosis onset in high-risk youths. *NPJ Schizophr.* 2015;1:15030. doi:10.1038/npjschz.2015.30.

13. Berger GE, Smesny S, Schäfer MR, et al. Niacin Skin Sensitivity Is Increased in Adolescents at Ultra-High Risk for Psychosis. *PLoS One* 2016;11(2):e0148429. doi:10.1371/journal.pone.0148429.

14. Bloemen OJN, de Koning MB, Schmitz N, et al. White-matter markers for psychosis in a prospective ultra-high-risk cohort. *Psychol. Med.* 2010;40(8):1297-1304. doi:10.1017/S0033291709991711.

15. Bodatsch M, Ruhrmann S, Wagner M, et al. Prediction of psychosis by mismatch negativity. *Biol. Psychiatry.* 2011;69(10):959-966. doi:10.1016/j.biopsych.2010.09.057.

16. Bousman CA, Yung AR, Pantelis C, et al. Effects of NRG1 and DAOA genetic variation on transition to psychosis in individuals at ultra-high risk for psychosis. *Transl. Psychiatry* 2013;3:e251. doi:10.1038/tp.2013.23.

17. Brewer WJ, Wood SJ, McGorry PD, et al. Impairment of olfactory identification ability in individuals at ultra-high risk for psychosis who later develop schizophrenia. *Am. J. Psychiatry* 2003;160(10):1790-1794. doi:10.1176/appi.ajp.160.10.1790.

18. Buchy L, Perkins D, Woods SW, Liu L, Addington J. Impact of substance use on conversion to psychosis in youth at clinical high risk of psychosis. *Schizophr. Res.* 2014;156(2-3):277-280. doi:10.1016/j.schres.2014.04.021.

19. Buchy L, Seidman LJ, Cadenhead KS, et al. Evaluating the relationship between cannabis use and IQ in youth and young adults at clinical high risk of psychosis. *Psychiatry Res.* 2015;230(3):878-884. doi:10.1016/j.psychres.2015.11.033.

20. Cannon TD, Cadenhead K, Cornblatt B, et al. Prediction of psychosis in youth at high clinical risk: a multisite longitudinal study in North America. *Arch. Gen. Psychiatry* 2008;65(1):28-37. doi:10.1001/archgenpsychiatry.2007.3.

21. Cannon TD, Yu C, Addington J, et al. An individualized risk calculator for research in prodromal psychosis. *Am. J. Psychiatry* 2016;173(10):980-988. doi:10.1176/appi.ajp.2016.15070890.

22. Cotter J, Lin A, Drake RJ, et al. Long-term employment among people at ultra-high risk for psychosis. *Schizophr. Res.* 2017;184:26-31. doi:10.1016/j.schres.2016.11.033.

23. Chung Y, Jacobson A, He G, et al. Prodromal symptom severity predicts accelerated gray matter reduction and third ventricle expansion among clinically high risk youth developing psychotic disorders. *Mol. Neuropsychiatry* 2015;1(1):13-22. doi:10.1159/000371887.

24. Corcoran CM, Keilp JG, Kayser J, et al. Emotion recognition deficits as predictors of transition in individuals at clinical high risk for schizophrenia: a neurodevelopmental perspective. *Psychol. Med.* 2015;45(14):2959-2973. doi:10.1017/S0033291715000902.

25. Cornblatt BA, Carrión RE, Auther A, et al. Psychosis prevention: A modified clinical high risk perspective from the recognition and prevention (RAP) program. *Am. J. Psychiatry* 2015;172(10):986-994. doi:10.1176/appi.ajp.2015.13121686.

26. Crump FM, Arndt L, Grivel M, et al. Attenuated first-rank symptoms and conversion to psychosis in a clinical high-risk cohort. *Early Interv. Psychiatry* 2017. doi:10.1111/eip.12529.

27. Dazzan P, Soulsby B, Mechelli A, et al. Volumetric abnormalities predating the onset of schizophrenia and affective psychoses: an MRI study in subjects at ultrahigh risk of psychosis. *Schizophr. Bull.* 2012;38(5):1083-1091. doi:10.1093/schbul/sbr035.

28. Deighton S, Buchy L, Cadenhead KS, et al. Traumatic brain injury in individuals at clinical high risk for psychosis. *Schizophr. Res.* 2016;174(1-3):77-81. doi:10.1016/j.schres.2016.04.041.

29. Demjaha A, Valmaggia L, Stahl D, Byrne M, McGuire P. Disorganization/cognitive and negative symptom dimensions in the at-risk mental state predict subsequent transition to psychosis. *Schizophr. Bull.* 2012;38(2):351-359. doi:10.1093/schbul/sbq088.

30. DeVylder JE, Muchomba FM, Gill KE, et al. Symptom trajectories and psychosis onset in a clinical high-risk cohort: the relevance of subthreshold thought disorder. *Schizophr. Res.* 2014;159(2-3):278-283. doi:10.1016/j.schres.2014.08.008.

31. Dragt S, Nieman DH, Veltman D, et al. Environmental factors and social adjustment as predictors of a first psychosis in subjects at ultra high risk. *Schizophr. Res.* 2011;125(1):69-76. doi:10.1016/j.schres.2010.09.007.

32. Föcking M, Dicker P, Lopez LM, et al. Differential expression of the inflammation marker IL12p40 in the at-risk mental state for psychosis: a predictor of transition to psychotic disorder? *BMC Psychiatry* 2016;16(1):326. doi:10.1186/s12888-016-1039-7.

33. Francey SM, Jackson HJ, Phillips LJ, Wood SJ, Yung AR, McGorry PD. Sustained attention in young people at high risk of psychosis does not predict transition to psychosis. *Schizophr. Res.* 2005;79(1):127-136. doi:10.1016/j.schres.2005.06.023.

34. Fusar-Poli P, Byrne M, Valmaggia L, et al. Social dysfunction predicts two years clinical outcome in people at ultra high risk for psychosis. *J. Psychiatr. Res.* 2010;44(5):294-301. doi:10.1016/j.jpsychires.2009.08.016.

35. Garner B, Pariante CM, Wood SJ, et al. Pituitary volume predicts future transition to psychosis in individuals at ultra-high risk of developing psychosis. *Biol. Psychiatry.* 2005;58(5):417-423. doi:10.1016/j.biopsych.2005.04.018.

36. Grivel MM, Leong W, Masucci MD, et al. Impact of lifetime traumatic experiences on suicidality and likelihood of conversion in a cohort of individuals at clinical high-risk for psychosis. *Schizophr. Res.* 2018;195:549-553. doi:10.1016/j.schres.2017.09.006.

37. Gschwandtner U, Pflueger MO, Semenin V, Gaggiotti M, Riecher-Rössler A, Fuhr P. EEG: a helpful tool in the prediction of psychosis. *Eur Arch Psychiatry Clin Neurosci* 2009;259(5):257-262. doi:10.1007/s00406-008-0854-3.

38. Haroun N, Dunn L, Haroun A, Cadenhead KS. Risk and protection in prodromal schizophrenia: ethical implications for clinical practice and future research. *Schizophr. Bull.* 2006;32(1):166-178. doi:10.1093/schbul/sbj007.

39. Healey KM, Penn DL, Perkins D, Woods SW, Addington J. Theory of mind and social judgments in people at clinical high risk of psychosis. *Schizophr. Res.* 2013;150(2-3):498-504. doi:10.1016/j.schres.2013.08.038.

40. Heinze K, Reniers RLEP, Nelson B, et al. Discrete alterations of brain network structural covariance in individuals at ultra-high risk for psychosis. *Biol. Psychiatry.* 2015;77(11):989-996. doi:10.1016/j.biopsych.2014.10.023.

41. Hengartner MP, Heekeren K, Dvorsky D, Walitza S, Rössler W, Theodoridou A. Course of psychotic symptoms, depression and global functioning in persons at clinical high risk of psychosis: Results of a longitudinal observation study over three years focusing on both converters and non-converters. *Schizophr. Res.* 2017;189:19-26. doi:10.1016/j.schres.2017.01.040.

42. Higuchi Y, Sumiyoshi T, Seo T, Miyanishi T, Kawasaki Y, Suzuki M. Mismatch negativity and cognitive performance for the prediction of psychosis in subjects with at-risk mental state. *PLoS One* 2013;8(1):e54080. doi:10.1371/journal.pone.0054080.

43. Hur JW, Choi S-H, Yun J-Y, Chon M-W, Kwon JS. Parental socioeconomic status and prognosis in individuals with ultra-high risk for psychosis: A 2-year follow-up study. *Schizophr. Res.* 2015;168(1-2):56-61. doi:10.1016/j.schres.2015.07.020.

44. Ising HK, Ruhrmann S, Burger NAFM, et al. Development of a stage-dependent prognostic model to predict psychosis in ultra-high-risk patients seeking treatment for co-morbid psychiatric disorders. *Psychol. Med.* 2016;46(9):1839-1851. doi:10.1017/S0033291716000325.

45. Keefe RSE, Poe M, Walker TM, Kang JW, Harvey PD. The Schizophrenia Cognition Rating Scale: an interview-based assessment and its relationship to cognition, real-world functioning, and functional capacity. *Am. J. Psychiatry* 2006;163(3):426-432. doi:10.1176/appi.ajp.163.3.426.

46. Kim HS, Shin NY, Jang JH, et al. Social cognition and neurocognition as predictors of conversion to psychosis in individuals at ultra-high risk. *Schizophr. Res.* 2011;130(1-3):170-175. doi:10.1016/j.schres.2011.04.023.

47. Korkeila J, Salokangas RKR, Heinimaaa M, et al. Physical illnesses, developmental risk factors and psychiatric diagnoses among subjects at risk of psychosis. *Eur. Psychiatry* 2013;28(3):135-140. doi:10.1016/j.eurpsy.2011.06.005.

48. Kotlicka-Antczak M, Pawełczyk A, Pawełczyk T, Strzelecki D, Żurner N, Karbownik MS. A history of obstetric complications is associated with the risk of progression from an at risk mental state to psychosis. *Schizophr. Res.* 2017. doi:10.1016/j.schres.2017.10.039.

49. Koutsouleris N, Meisenzahl EM, Davatzikos C, et al. Use of neuroanatomical pattern classification to identify subjects in at-risk mental states of psychosis and predict disease transition. *Arch. Gen. Psychiatry* 2009;66(7):700-712. doi:10.1001/archgenpsychiatry.2009.62.

50. Koutsouleris N, Davatzikos C, Bottlender R, et al. Early recognition and disease prediction in the at-risk mental states for psychosis using neurocognitive pattern classification. *Schizophr. Bull.* 2012;38(6):1200-1215. doi:10.1093/schbul/sbr037.

51. Koutsouleris N, Borgwardt S, Meisenzahl EM, Bottlender R, Möller H-J, Riecher-Rössler A. Disease prediction in the at-risk mental state for psychosis using neuroanatomical biomarkers: results from the FePsy study. *Schizophr. Bull.* 2012;38(6):1234-1246. doi:10.1093/schbul/sbr145.

52. Kraan T, van Dam DS, Velthorst E, et al. Childhood trauma and clinical outcome in patients at ultra-high risk of transition to psychosis. *Schizophr. Res.* 2015;169(1-3):193-198. doi:10.1016/j.schres.2015.10.030.

53. Kraan TC, Ising HK, Fokkema M, et al. The effect of childhood adversity on 4-year outcome in individuals at ultra high risk for psychosis in the Dutch Early Detection Intervention Evaluation (EDIE-NL) Trial. *Psychiatry Res.* 2017;247:55-62. doi:10.1016/j.psychres.2016.11.014.

54. Kraan TC, Velthorst E, Themmen M, et al. Child Maltreatment and Clinical Outcome in Individuals at Ultra-High Risk for Psychosis in the EU-GEI High Risk Study. *Schizophr. Bull.* 2017;44(3):584-592. doi:10.1093/schbul/sbw162.

55. Kristensen K, Cadenhead KS. Cannabis abuse and risk for psychosis in a prodromal sample. *Psychiatry Res.* 2007;151(1-2):151-154. doi:10.1016/j.psychres.2006.10.001.

56. Kwon JS, Byun MS, Lee TY, An SK. Early intervention in psychosis: Insights from Korea. *Asian J. Psychiatr.* 2012;5(1):98-105. doi:10.1016/j.ajp.2012.02.007.

57. Labad J, Stojanovic-Pérez A, Montalvo I, et al. Stress biomarkers as predictors of transition to psychosis in at-risk mental states: roles for cortisol, prolactin and albumin. *J. Psychiatr. Res.* 2015;60:163-169. doi:10.1016/j.jpsychires.2014.10.011.

58. Lavoie S, Schäfer MR, Whitford TJ, et al. Frontal delta power associated with negative symptoms in ultra-high risk individuals who transitioned to psychosis. *Schizophr. Res.* 2012;138(2-3):206-211. doi:10.1016/j.schres.2012.03.033.

59. Lavoie S, Bartholomeuz CF, Nelson B, et al. Sulcogyral pattern and sulcal count of the orbitofrontal cortex in individuals at ultra high risk for psychosis. *Schizophr. Res.* 2014;154(1-3):93-99. doi:10.1016/j.schres.2014.02.008.

60. Lee TY, Shin YS, Shin NY, et al. Neurocognitive function as a possible marker for remission from clinical high risk for psychosis. *Schizophr. Res.* 2014;153(1-3):48-53. doi:10.1016/j.schres.2014.01.018.

61. Lehembre-Shiah E, Leong W, Brucato G, et al. Distinct Relationships Between Visual and Auditory Perceptual Abnormalities and Conversion to Psychosis in a Clinical High-Risk Population. *JAMA Psychiatry* 2017;74(1):104-106. doi:10.1001/jamapsychiatry.2016.3055.

62. Lemos-Giráldez S, Vallina-Fernández O, Fernández-Iglesias P, et al. Symptomatic and functional outcome in youth at ultra-high risk for psychosis: a longitudinal study. *Schizophr. Res.* 2009;115(2-3):121-129. doi:10.1016/j.schres.2009.09.011.

63. Li H, Zhang T, Xu L, et al. A comparison of conversion rates, clinical profiles and predictors of outcomes in two independent samples of individuals at clinical high risk for psychosis in China. *Schizophr. Res.* 2017. doi:10.1016/j.schres.2017.11.029.

64. Lim J, Rekhi G, Rapisarda A, et al. Impact of psychiatric comorbidity in individuals at Ultra High Risk of psychosis - Findings from the Longitudinal Youth at Risk Study (LYRIKS). *Schizophr. Res.* 2015;164(1-3):8-14. doi:10.1016/j.schres.2015.03.007.

65. Lim K-O, Lee TY, Kim M, et al. Early referral and comorbidity as possible causes of the declining transition rate in subjects at clinical high risk for psychosis. *Early Interv. Psychiatry* 2016. doi:10.1111/eip.12363.

66. Lin A, Yung AR, Nelson B, et al. Neurocognitive predictors of transition to psychosis: medium- to long-term findings from a sample at ultra-high risk for psychosis. *Psychol. Med.* 2013;43(11):2349-2360. doi:10.1017/S0033291713000123.

67. Lyngberg K, Buchy L, Liu L, Perkins D, Woods S, Addington J. Patterns of premorbid functioning in individuals at clinical high risk of psychosis. *Schizophr. Res.* 2015;169(1-3):209-213. doi:10.1016/j.schres.2015.11.004.

68. Mason O, Startup M, Halpin S, Schall U, Conrad A, Carr V. Risk factors for transition to first episode psychosis among individuals with “at-risk mental states”. *Schizophr. Res.* 2004;71(2-3):227-237. doi:10.1016/j.schres.2004.04.006.

69. McHugh MJ, McGorry PD, Yung AR, et al. Cannabis-induced attenuated psychotic symptoms: implications for prognosis in young people at ultra-high risk for psychosis. *Psychol. Med.* 2017;47(4):616-626. doi:10.1017/S0033291716002671.

70. McLaughlin D, Carrión RE, Auther AM, et al. Functional Capacity Assessed by the Map Task in Individuals at Clinical High-Risk for Psychosis. *Schizophr. Bull.* 2016;42(5):1234-1242. doi:10.1093/schbul/sbw039.

71. Mechelli A, Lin A, Wood S, et al. Using clinical information to make individualized prognostic predictions in people at ultra high risk for psychosis. *Schizophr. Res.* 2017;184:32-38. doi:10.1016/j.schres.2016.11.047.

72. Metzler S, Dvorsky D, Wyss C, et al. Neurocognition in help-seeking individuals at risk for psychosis: Prediction of outcome after 24 months. *Psychiatry Res.* 2016;246:188-194. doi:10.1016/j.psychres.2016.08.065.

73. Michel C, Ruhrmann S, Schimmelmann BG, Klosterkötter J, Schultze-Lutter F. A stratified model for psychosis prediction in clinical practice. *Schizophr. Bull.* 2014;40(6):1533-1542. doi:10.1093/schbul/sbu025.

74. Mittal VA, Walker EF. Movement abnormalities predict conversion to Axis I psychosis among prodromal adolescents. *J. Abnorm. Psychol.* 2007;116(4):796-803. doi:10.1037/0021-843X.116.4.796.

75. Mittal VA, Willhite R, Daley M, et al. Obstetric complications and risk for conversion to psychosis among individuals at high clinical risk. *Early Interv. Psychiatry* 2009;3(3):226-230. doi:10.1111/j.1751-7893.2009.00137.x.

76. Mittal VA, Walker EF, Bearden CE, et al. Markers of basal ganglia dysfunction and conversion to psychosis: neurocognitive deficits and dyskinesias in the prodromal period. *Biol. Psychiatry.* 2010;68(1):93-99. doi:10.1016/j.biopsych.2010.01.021.

77. Mittal VA, Daley M, Shiode MF, Bearden CE, O’Neill J, Cannon TD. Striatal volumes and dyskinetic movements in youth at high-risk for psychosis. *Schizophr. Res.* 2010;123(1):68-70. doi:10.1016/j.schres.2010.08.002.

78. Mourik K, Decrescenzo P, Brucato G, et al. Various neurocognitive deficits and conversion risk in individuals at clinical high risk for psychosis. *Early Interv. Psychiatry* 2017;11(3):250-254. doi:10.1111/eip.12296.

79. Nelson B, Thompson A, Yung AR. Basic self-disturbance predicts psychosis onset in the ultra high risk for psychosis “prodromal” population. *Schizophr. Bull.* 2012;38(6):1277-1287. doi:10.1093/schbul/sbs007.

80. Nieman DH, Rike WH, Becker HE, et al. Prescription of antipsychotic medication to patients at ultra high risk of developing psychosis. *Int Clin Psychopharmacol* 2009;24(4):223-228. doi:10.1097/YIC.0b013e32832e0a76.

81. Nieman DH, Velthorst E, Becker HE, et al. The Strauss and Carpenter Prognostic Scale in subjects clinically at high risk of psychosis. *Acta Psychiatr. Scand.* 2013;127(1):53-61. doi:10.1111/j.1600-0447.2012.01899.x.

82. Nieman DH, Ruhrmann S, Dragt S, et al. Psychosis prediction: stratification of risk estimation with information-processing and premorbid functioning variables. *Schizophr. Bull.* 2014;40(6):1482-1490. doi:10.1093/schbul/sbt145.

83. O’Donoghue B, Nelson B, Yuen HP, et al. Social environmental risk factors for transition to psychosis in an Ultra-High Risk population. *Schizophr. Res.* 2015;161(2-3):150-155. doi:10.1016/j.schres.2014.10.050.

84. Papmeyer M, Würsch I, Studerus E, Stieglitz R-D, Riecher-Rössler A. The role of vulnerability factors in individuals with an at-risk mental state of psychosis. *Neuropsychiatr* 2016;30(1):18-26. doi:10.1007/s40211-016-0179-9.

85. Pawełczyk T, Trafalska E, Kotlicka-Antczak M, Pawełczyk A. The association between polyunsaturated fatty acid consumption and the transition to psychosis in ultra-high risk individuals. *Prostaglandins Leukot Essent Fatty Acids* 2016;108:30-37. doi:10.1016/j.plefa.2016.03.010.

86. Perez VB, Woods SW, Roach BJ, et al. Automatic auditory processing deficits in schizophrenia and clinical high-risk patients: forecasting psychosis risk with mismatch negativity. *Biol. Psychiatry.* 2014;75(6):459-469. doi:10.1016/j.biopsych.2013.07.038.

87. Phillips LJ, Curry C, Yung AR, Yuen HP, Adlard S, McGorry PD. Cannabis use is not associated with the development of psychosis in an “ultra” high-risk group. *Aust. N. Z. J. Psychiatry* 2002;36(6):800-806. doi:10.1046/j.1440-1614.2002.01089.x.

88. Phillips LJ, Velakoulis D, Pantelis C, et al. Non-reduction in hippocampal volume is associated with higher risk of psychosis. *Schizophr. Res.* 2002;58(2-3):145-158.

89. Piskulic D, Addington J, Cadenhead KS, et al. Negative symptoms in individuals at clinical high risk of psychosis. *Psychiatry Res.* 2012;196(2-3):220-224. doi:10.1016/j.psychres.2012.02.018.

90. Power L, Polari AR, Yung AR, McGorry PD, Nelson B. Distress in relation to attenuated psychotic symptoms in the ultra-high-risk population is not associated with increased risk of psychotic disorder. *Early Interv. Psychiatry* 2016;10(3):258-262. doi:10.1111/eip.12233.

91. Ramyead A, Studerus E, Kometer M, et al. Prediction of psychosis using neural oscillations and machine learning in neuroleptic-naïve at-risk patients. *World J Biol Psychiatry* 2016;17(4):285-295. doi:10.3109/15622975.2015.1083614.

92. Rekhi G, Rapisarda A, Lee J. Impact of distress related to attenuated psychotic symptoms in individuals at ultra high risk of psychosis: Findings from the Longitudinal Youth at Risk Study. *Early Interv. Psychiatry* 2017. doi:10.1111/eip.12451.

93. Riecher-Rössler A, Pflueger MO, Aston J, et al. Efficacy of using cognitive status in predicting psychosis: a 7-year follow-up. *Biol. Psychiatry.* 2009;66(11):1023-1030. doi:10.1016/j.biopsych.2009.07.020.

94. Ruhrmann S, Schultze-Lutter F, Bechdolf A, Klosterkötter J. Intervention in at-risk states for developing psychosis. *Eur Arch Psychiatry Clin Neurosci* 2010;260 Suppl 2:S90-4. doi:10.1007/s00406-010-0139-5.

95. Rüsch N, Heekeren K, Theodoridou A, et al. Stigma as a stressor and transition to schizophrenia after one year among young people at risk of psychosis. *Schizophr. Res.* 2015;166(1-3):43-48. doi:10.1016/j.schres.2015.05.027.

96. Sabb FW, van Erp TGM, Hardt ME, et al. Language network dysfunction as a predictor of outcome in youth at clinical high risk for psychosis. *Schizophr. Res.* 2010;116(2-3):173-183. doi:10.1016/j.schres.2009.09.042.

97. Salokangas RKR, Ruhrmann S, von Reventlow HG, et al. Axis I diagnoses and transition to psychosis in clinical high-risk patients EPOS project: prospective follow-up of 245 clinical high-risk outpatients in four countries. *Schizophr. Res.* 2012;138(2-3):192-197. doi:10.1016/j.schres.2012.03.008.

98. Salokangas RKR, Dingemans P, Heinimaa M, et al. Prediction of psychosis in clinical high-risk patients by the Schizotypal Personality Questionnaire. Results of the EPOS project. *Eur. Psychiatry* 2013;28(8):469-475. doi:10.1016/j.eurpsy.2013.01.001.

99. Schultze-Lutter F, Klosterkötter J, Ruhrmann S. Improving the clinical prediction of psychosis by combining ultra-high risk criteria and cognitive basic symptoms. *Schizophr. Res.* 2014;154(1-3):100-106. doi:10.1016/j.schres.2014.02.010.

100. Seidman LJ, Giuliano AJ, Meyer EC, et al. Neuropsychology of the prodrome to psychosis in the NAPLS consortium: relationship to family history and conversion to psychosis. *Arch. Gen. Psychiatry* 2010;67(6):578-588. doi:10.1001/archgenpsychiatry.2010.66.

101. Seidman LJ, Shapiro DI, Stone WS, et al. Association of neurocognition with transition to psychosis: baseline functioning in the second phase of the north american prodrome longitudinal study. *JAMA Psychiatry* 2016;73(12):1239-1248. doi:10.1001/jamapsychiatry.2016.2479.

102. Simeonova DI, Attalla A, Trotman H, Esterberg M, Walker EF. Does a parent-report measure of behavioral problems enhance prediction of conversion to psychosis in clinical high-risk adolescents? *Schizophr. Res.* 2011;130(1-3):157-163. doi:10.1016/j.schres.2011.03.034.

103. Stowkowy J, Liu L, Cadenhead KS, et al. Early traumatic experiences, perceived discrimination and conversion to psychosis in those at clinical high risk for psychosis. *Soc. Psychiatry Psychiatr. Epidemiol.* 2016;51(4):497-503. doi:10.1007/s00127-016-1182-y.

104. Takahashi T, Wood SJ, Yung AR, et al. Superior temporal gyrus volume in antipsychotic-naive people at risk of psychosis. *Br. J. Psychiatry* 2010;196(3):206-211. doi:10.1192/bjp.bp.109.069732.

105. Takahashi T, Wood SJ, Yung AR, et al. Altered depth of the olfactory sulcus in ultra high-risk individuals and patients with psychotic disorders. *Schizophr. Res.* 2014;153(1-3):18-24. doi:10.1016/j.schres.2014.01.041.

106. Tamagni C, Studerus E, Gschwandtner U, Aston J, Borgwardt S, Riecher-Rössler A. Are neurological soft signs pre-existing markers in individuals with an at-risk mental state for psychosis? *Psychiatry Res.* 2013;210(2):427-431. doi:10.1016/j.psychres.2013.06.016.

107. Tarbox SI, Addington J, Cadenhead KS, et al. Premorbid functional development and conversion to psychosis in clinical high-risk youths. *Dev. Psychopathol.* 2013;25(4 Pt 1):1171-1186. doi:10.1017/S0954579413000448.

108. Thompson A, Nelson B, Yung A. Predictive validity of clinical variables in the “at risk” for psychosis population: international comparison with results from the North American Prodrome Longitudinal Study. *Schizophr. Res.* 2011;126(1-3):51-57. doi:10.1016/j.schres.2010.09.024.

109. Thompson AD, Nelson B, Yuen HP, et al. Sexual trauma increases the risk of developing psychosis in an ultra high-risk “prodromal” population. *Schizophr. Bull.* 2014;40(3):697-706. doi:10.1093/schbul/sbt032.

110. Valmaggia LR, Day FL, Jones C, et al. Cannabis use and transition to psychosis in people at ultra-high risk. *Psychol. Med.* 2014;44(12):2503-2512. doi:10.1017/S0033291714000117.

111. van Tricht MJ, Nieman DH, Koelman JHTM, et al. Reduced parietal P300 amplitude is associated with an increased risk for a first psychotic episode. *Biol. Psychiatry.* 2010;68(7):642-648. doi:10.1016/j.biopsych.2010.04.022.

112. van Tricht MJ, Ruhrmann S, Arns M, et al. Can quantitative EEG measures predict clinical outcome in subjects at Clinical High Risk for psychosis? A prospective multicenter study. *Schizophr. Res.* 2014;153(1-3):42-47. doi:10.1016/j.schres.2014.01.019.

113. Velthorst E, Nieman DH, Becker HE, et al. Baseline differences in clinical symptomatology between ultra high risk subjects with and without a transition to psychosis. *Schizophr. Res.* 2009;109(1-3):60-65. doi:10.1016/j.schres.2009.02.002.

114. Velthorst E, Nieman DH, Linszen D, et al. Disability in people clinically at high risk of psychosis. *Br. J. Psychiatry* 2010;197(4):278-284. doi:10.1192/bjp.bp.109.075036.

115. Velthorst E, Nelson B, Wiltink S, et al. Transition to first episode psychosis in ultra high risk populations: does baseline functioning hold the key? *Schizophr. Res.* 2013;143(1):132-137. doi:10.1016/j.schres.2012.10.025.

116. Walder DJ, Holtzman CW, Addington J, et al. Sexual dimorphisms and prediction of conversion in the NAPLS psychosis prodrome. *Schizophr. Res.* 2013;144(1-3):43-50. doi:10.1016/j.schres.2012.11.039.

117. Walker EF, Brennan PA, Esterberg M, Brasfield J, Pearce B, Compton MT. Longitudinal changes in cortisol secretion and conversion to psychosis in at-risk youth. *J. Abnorm. Psychol.* 2010;119(2):401-408. doi:10.1037/a0018399.

118. Walterfang M, Yung A, Wood AG, et al. Corpus callosum shape alterations in individuals prior to the onset of psychosis. *Schizophr. Res.* 2008;103(1-3):1-10. doi:10.1016/j.schres.2008.04.042.

119. Yun Y, Phillips LJ, Cotton S, et al. Obstetric complications and transition to psychosis in an “ultra” high risk sample. *Aust. N. Z. J. Psychiatry* 2005;39(6):460-466. doi:10.1080/j.1440-1614.2005.01604.x.

120. Yung AR, Phillips LJ, McGorry PD, et al. Prediction of psychosis. A step towards indicated prevention of schizophrenia. *Br. J. Psychiatry. Suppl.* 1998;172(33):14-20.

121. Yung AR, Phillips LJ, Yuen HP, McGorry PD. Risk factors for psychosis in an ultra high-risk group: psychopathology and clinical features. *Schizophr. Res.* 2004;67(2-3):131-142. doi:10.1016/S0920-9964(03)00192-0.

122. Yung AR, Yung AR, Pan Yuen H, et al. Mapping the onset of psychosis: the Comprehensive Assessment of At-Risk Mental States. *Aust NZ J Psychiatry* 2005;39(11-12):964-971. doi:10.1080/j.1440-1614.2005.01714.x.

123. Zhang T, Li H, Stone WS, et al. Neuropsychological Impairment in Prodromal, First-Episode, and Chronic Psychosis: Assessing RBANS Performance. *PLoS One* 2015;10(5):e0125784. doi:10.1371/journal.pone.0125784.

124. Zhang TH, Li HJ, Woodberry KA, et al. Two-year follow-up of a Chinese sample at clinical high risk for psychosis: timeline of symptoms, help-seeking and conversion. *Epidemiol Psychiatr Sci* 2017;26(3):287-298. doi:10.1017/S2045796016000184.

125. Ziermans TB, Schothorst PF, Sprong M, van Engeland H. Transition and remission in adolescents at ultra-high risk for psychosis. *Schizophr. Res.* 2011;126(1-3):58-64. doi:10.1016/j.schres.2010.10.022.

126. Ziermans TB, Schothorst PF, Schnack HG, et al. Progressive structural brain changes during development of psychosis. *Schizophr. Bull.* 2012;38(3):519-530. doi:10.1093/schbul/sbq113.

127. Ziermans T, de Wit S, Schothorst P, et al. Neurocognitive and clinical predictors of long-term outcome in adolescents at ultra-high risk for psychosis: a 6-year follow-up. *PLoS One* 2014;9(4):e93994. doi:10.1371/journal.pone.0093994.

128. Brucato G, Masucci MD, Arndt LY, et al. Baseline demographics, clinical features and predictors of conversion among 200 individuals in a longitudinal prospective psychosis-risk cohort. *Psychol. Med.* 2017;47(11):1923-1935. doi:10.1017/S0033291717000319.
